# A concept analysis of the term midwifery intervention: A scoping review

**DOI:** 10.18332/ejm/216185

**Published:** 2026-03-31

**Authors:** Marianne J. Nieuwenhuijze, Helga Gottfreðsdóttir

**Affiliations:** 1 Research Centre for Midwifery Science, Zuyd University, Maastricht, The Netherlands; 2 Care and Public Health Research Institute, Maastricht University, Maastricht, The Netherlands; 3 Faculty of Nursing and Midwifery, School of Health Sciences, University of Iceland, Reykjavik, Iceland

**Keywords:** midwifery intervention, midwife intervention, concept analysis

## Abstract

**INTRODUCTION:**

The term midwifery intervention or midwife intervention regularly occurs in scientific literature, without consistent understanding of what it implies. In modern maternity care where new technological interventions are constantly applied in practice, it is important to identify how interventions can be built on midwifery knowledge and its philosophy of care. The aim of this study was to explore the concept of midwifery intervention to gain a deeper understanding of what the concept implies and how it is used.

**METHODS:**

We performed a scoping review for a concept analysis using Morse’s approach. We searched five databases for scientific publications where ‘midwifery’/‘midwife’ and ‘intervention’ are linked as one joint concept. Selected publications were evaluated using criteria for concept analysis: presence of definitions, defining attributes, boundaries, antecedents and consequences. Subsequently, we performed a critical analysis using ICM Philosophy of Midwifery Care.

**RESULTS:**

Our analysis of 83 selected papers show that the concept of midwifery intervention is not well defined and lacks a harmonious understanding. We see a mix of terms and the midwifery component of the intervention is poorly defined and mostly refers to midwives as merely performing the intervention. Critical analysis shows that six of the midwifery interventions strongly relate to the components of the ICM Philosophy of Midwifery Care.

**CONCLUSIONS:**

The way the term midwifery intervention is often used undermines a wider understanding of what midwifery is about, the added value midwives have for women and newborns, and the positing of midwives in healthcare. The terms should be used for intervention closely linked to the midwifery philosophy care.

## INTRODUCTION

Most of the work of midwives is providing care to women and their newborns to achieve optimal health outcomes^1^. In the past decades, interventions have become a common part of the care for women and newborns before, during and after birth^2^.

Generally, interventions are defined as actions of becoming intentionally involved in a difficult situation, to improve it or prevent it from getting worse^3^. In this study, we consider interventions in maternity care as measurable actions that are offered or taken up by one or more participants as a potentially legitimate strategy for solving a problem or promoting wellbeing, such as relieving pain, smoking cessation or group pregnancy care^4^. Interventions during pregnancy or childbirth are often labelled as medical interventions, obstetric interventions^5^, or as psychological or educational interventions^6^, suggesting that a specific body of knowledge is behind these interventions.

High quality maternity care implies appropriate use of interventions, which entails that interventions are evidence based, disseminated, implemented systematically and scientifically evaluated^1,7^. These interventions should promote a wide range of positive outcomes of childbirth for both women and newborns, including physical, mental or social wellbeing. However, there is a debate if interventions in maternity care are always used in the right way, at the right time, for the right person as argued in the publication of Miller et al.^2^. It seems that interventions are regularly used too much, too soon in certain groups, while too little, too late in others.

Midwifery looks critically at intervening in the natural process of pregnancy and birth, because a physiological pregnancy and birth have short- and long-term benefits for the mother, child, family and society^8^. However, midwives also intervene, for example when they encourage women to walk around during labor to stimulate and support the physiological process of childbirth. This is sometimes indicated as a midwifery intervention. Additionally, it is also acknowledged that interventions may be indicated when the natural birthing process no longer proceeds the physiological course and pathology becomes apparent or is expected.

The term midwifery intervention or midwife intervention regularly occurs in the literature. Sometimes it seems to indicate that it is a midwife who performs the intervention^9^, other times it seems to imply that a philosophy or specific knowledge base is behind it^10^. Still, it is not clear with what meaning these labels are applied. What does the concept of midwifery intervention mean, what makes it appropriate to add the label midwifery to an intervention? A clear definition and description of midwifery intervention as a concept seems to be missing. The International Confederation of Midwives (ICM) in one of their core documents defines midwifery as an approach to care for women and their newborns whereby midwives optimize the normal biological, psychological, social and cultural processes of childbirth and early life of the newborn; where they work in partnership with women; where they promote women’s personal capabilities to care for themselves and their families, and where they collaborate with other health professionals as necessary to provide holistic care that meets each woman’s individual needs^11^. This approach to care is further elaborated in their document on the Philosophy and Model of Midwifery Care^12^. The Lancet series on midwifery care also describes the scope of midwifery emphasizing the significance of preventive and supportive care that works to strengthen women’s capabilities in the context of respectful relationships, that is tailored to women’s needs and focuses on promotion of normal reproductive processes^8^. However, none of the documents links it to the concept of midwifery intervention.

In modern maternity care where new technological interventions are constantly applied in practice, it is important to identify how certain interventions, named as midwifery interventions, are built on midwifery knowledge and its philosophy of care^13^. However, much is unclear in how these labels are conceptualized and used. In this study, we will explore the concept of midwifery intervention to gain a clearer picture of its meaning. Through a scoping review and evaluation of the literature, we aim to contribute to a deeper understanding of what the concept implies and how it is used. More clarity will be beneficial for midwifery research and education, as concepts form the basis of a discipline and the essence of the philosophical footings that direct a profession^14^. A clear understanding also contributes to the implementation of midwifery innovations and policies in maternity care.

## METHODS

We conducted a scoping review for a concept analysis of the concept of midwifery intervention. It quickly became clear from a first exploration of the literature that the term midwifery intervention is used in different contexts, attributing different meanings to the term midwifery. This suggests that the concept is still far from mature^15^. According to Morse et al.^15,16^, a concept needs to be mature to use it unambiguously in research or policy making to prevent misunderstandings. A mature concept is well-defined and has clearly described defining attributes (characteristics), boundaries, antecedents (preconditions) and consequences (outcomes).

Therefore, we decided to use Morse’s approach to a concept evaluation, where the presentation of a concept in relevant literature is evaluated using evaluation criteria, including assessment of its definition, its defining attributes, boundaries, antecedents and consequences.

### Search and selection

Up to March 2024, we searched for scientific publications where ‘midwifery’/‘midwife’ and ‘intervention’ are linked as one joint concept (e.g. midwifery intervention, midwife-led intervention or midwifery counselling intervention). We used the databases PubMed, Embase, CINAHL, PsychINFO and SocialINDEX and applied no publication date or language limitations (Supplementary file Part 1). We hand searched the reference lists of included publications. Additionally, we searched for grey literature limiting this searching the website of the ICM and professional midwifery organizations in the UK, Ireland, Canada, USA, New Zealand and Australia.

The two authors separately did a first selection based on title and abstract. All publications that seemed relevant by at least one author were include in the full-text screening. Subsequently, we obtained the full text of the selected publications and reviewed these by both authors separately reading the full article. Inclusion criteria were publications that mentioned midwife(ry) and intervention as a joint concept, offered some description of this concept and were written in English. If disagreeing on inclusion, the publication was discussed until consensus was reached. No assessment of the methodological quality of the individual publications was done as we are not analyzing the outcomes of the studies as such, but the way the concept of midwifery intervention is described in the texts.

While exploring the publications that arose in our search, we identified other terms that indicated interventions, such as ‘services’ (e.g. midwife-led postnatal debriefing services). We decided not to include these in our selection as our primary interest was the concept of midwifery intervention.

We followed the PRISMA (Preferred Recording Items for Systematic reviews and Meta-analysis) statement for conducting and recording the inclusion/exclusion process^17^.

### Concept analysis

Morse et al.^16^ describe a concept analysis as ‘the process of unfolding, exploring, and understanding concepts for the purposes of concept development, delineation, comparison, clarification, correction, identification, refinement, and validation.

In our concept analysis, we took a two-step approach. In step one, we looked for definitions, attributes, antecedents and consequences to evaluate how the concept is described in the included publications and to conclude on the present understanding of the concept. We used this to evaluate the maturity of the concept. In step two, we performed a critical analysis of the included publications, searching for those that could give a deeper understanding of what the concept of midwifery intervention entails. With this step, we aim to develop a further understanding and validation of the concept midwifery intervention.

*Evaluation of the concept as used in scientific literature (step one)*

We extracted and analyzed the included publications to generate defining attributes, boundaries, antecedents and consequences. We created two data extraction tables. One in which we collected information on the characteristics of the included publications, such as author(s), title, publication year, country, type of article, aim, context and setting, population and way of data collection (Supplementary file Part 2). In the other table, we collected information from the publications on the evaluation criteria for the concept midwifery intervention, such as the exact naming of the intervention, the definition, defining attributes, boundaries, antecedents and consequences (Supplementary file Part 3). Subsequently, we synthesized these findings and evaluated the maturity of the concept as Morse et al.15 suggested.

*Critical analysis of the concept midwifery intervention (step two)*

We explored all included publications using several analytical questions that would help in a further understanding of the concept:

- What is the theoretical background of the intervention?
- How was the intervention developed?
- What does the intervention look like and how is it used?
- How is the intervention specifically connected to midwifery?
- How does the intervention relate to the ICM midwifery philosophy of care?

To answer the last question, we explored how the description of the concept midwifery intervention in the included publications related to the Philosophy of Midwifery Care as documented by the ICM to determine what makes an intervention a midwifery intervention^12^ (Supplementary file Part 4). The decision to use the ICM philosophy was made considering that the ICM work is known and respected worldwide, and that it describes a philosophy that refers to all aspects of midwifery.

## RESULTS

**Figure 1:**
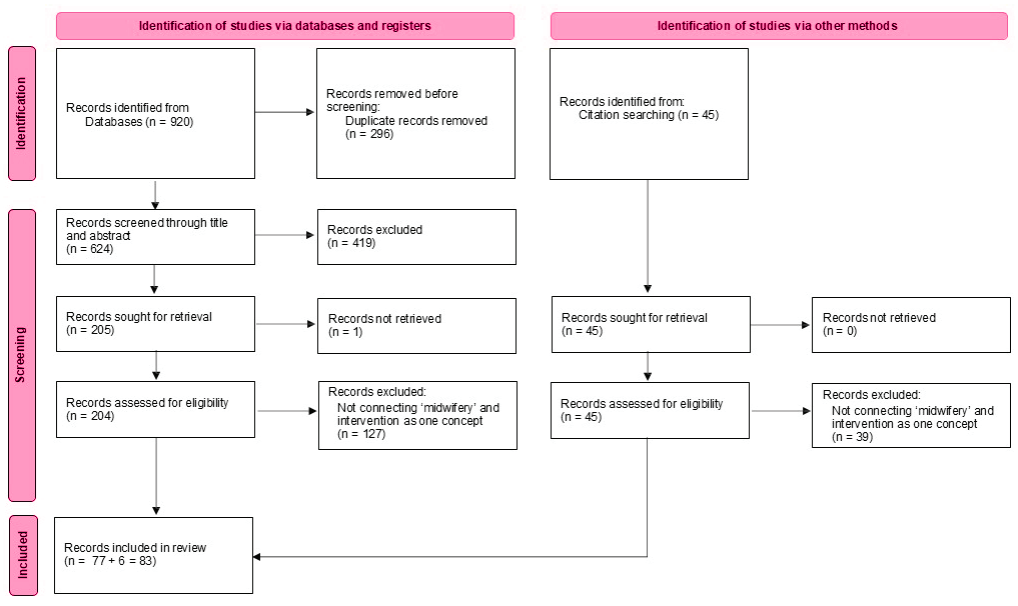
Prisma of the search and selection process

From our search, yielding 624 unique hits, we excluded 419 publications based on title and abstract as they did not address the topic of our scoping review. Subsequently, we excluded one publication because we could not retrieve the full text and 127 publications based on full text because ‘midwifery’ and ‘intervention’ were not connected as one concept. In our scoping review, we included 83 full publications, which gave further information about the concept^9,10,18-97^. No documentations of the concept were found on the website of the ICM and professional midwifery organizations in the UK, Ireland, Canada, USA, New Zealand and Australia. Figure 1 shows the selection process of the 83 selected publications.

Table 1 presents a summary of the characteristics and findings of these publications. More details can be found in Supplementary file Part 2.

**Table 1:** Summary of the most significant characteristics and findings of the selected publications

*Authors Year*	*Aim*	*Exact naming of the intervention*	*Content orientation of intervention*	*Definition*
Abhari et al.^18^ 2020	To investigate the effects of counseling based on Gamble’s approach on psychological birth trauma.	Midwifery-led counseling intervention	Maternal mental health	A counseling intervention to prevent and mitigate psychological birth trauma.
Abou Malham et al.^19^ 2015	To identify barriers that could potentially hinder an action plan to strengthen the midwifery professional role.	Midwifery intervention	Professional role	An intervention (action plan) aiming to strengthen the midwifery professional role.
Adams et al.^20^ 2017	To determine if women receiving an educational intervention Centering-Pregnancy Oral Health Promotion had clinically improved oral health.	Nurse-midwife-led oral health intervention	Public health	An intervention embedded in Centering-Pregnancy to promote oral health.
Ajuebor et al.^21^ 2019	To assess the progress of the Global Strategic Directions for Strengthening Nursing and Midwifery 2016–2020 (SDNM) implementation at country level.	Nursing and midwifery policy intervention Nursing and midwifery intervention	Professional role	Intervention/program to address the specific obstacles to optimized contributions of nurses and midwives to universal health coverage and sustainable development goals.
Alderdice et al.^22^ 2013	To identify non-invasive interventions in the perinatal period that enable midwives to offer support to women on maternal mental health and well-being.	A midwifery-led intervention Midwifery intervention	Maternal mental health	Non-invasive interventions in the perinatal period that enable midwives to offer effective care to women within the area of maternal mental health and well-being.
Allen et al.^23^ 2016	To identify possible mechanisms by which caseload midwifery reduces preterm birth for young childbearing women.	Complex intervention of caseload midwifery	Organization of care	The intervention provided caseload midwifery.
Allen et al.^24^ 2017	To explore whether women allocated to caseload care characterize their midwife differently to those allocated to standard care.	Intervention of case-load midwifery	Organization of care	Caseload midwifery with relational continuity in antenatal, intrapartum and postnatal care from a primary midwife and back-up midwives.
Altiner et al.^25^ 2019	To develop and test a method for describing intervention content of nurse/midwife multitasked interventions using the Omaha System and Time Capture Tool.	Multitasking midwifery/nurse intervention	Professional role	Any task done by midwives in the center are regarded as midwifery intervention.
Asadzadeh et al.^26^ 2020	To investigate the effectiveness of a brief midwife-led counseling based on Gamble’s approach in decreasing post-traumatic stress disorder, depression, and anxiety symptoms among women with a traumatic childbirth.	A midwife-led brief counseling intervention	Maternal mental health	Intervention for postpartum women experiencing PTSD symptoms. The intervention approach was based on Gamble and colleagues’ protocol.
Bick et al.^27^ 2022	To assess the feasibility a future trial of a midwifery-led antenatal intervention to support women to perform PFME in pregnancy and reduce UI postnatally.	A midwife-led antenatal intervention	Public health	An intervention to support women to perform pelvic floor muscle exercises in pregnancy.
Blomgren et al.^28^ 2023	To describe the co-creation process and needs and determinants of a midwife-led quality improvement targeting evidence-based midwifery practices.	A midwife-led quality improvement intervention	Professional role	The intervention targets to bridge the evidence-to-practice gap within midwifery and effectively apply this across different settings.
Borg Cunen et al.^29^ 2014	To systematically identify interventions that midwives could introduce to address post-traumatic stress in women following childbirth.	Midwife-led intervention	Maternal mental health	Interventions that could be implemented by a midwife to support women with post traumatic stress after childbirth.
Borges^30^ 2021	To examine whether to invest in caseload midwifery for women with cystic fibrosis, in addition to care from obstetric and specialist teams.	Caseload midwifery as a legitimate intervention	Organization of care	An intervention by midwives that improves health outcomes in pregnant women with cystic fibrosis.
Borneskog et al.^31^ 2023	To examine how educators perceived using OSCA as an assessment device in midwifery education for performance in life-saving midwifery interventions.	Life-saving midwifery intervention	Professional role	No definition
Bryce et al.^32^ 2009	To assess a supportive midwifery intervention, Community Action on Tobacco for Children’s Health, to help young pregnant smokers quit.	A home-based midwifery intervention A supportive midwifery intervention	Public health	A responsive smoking cessation service that would meet the particular needs of pregnant women aged 25 years and under.
Caelli et al.^33^ 2002	To explore the impact of the Special Delivery Service (SDS), a midwife-managed intervention, as an addition to routine care to support and educate high-risk pregnant women and their partners subsequent to the death of a baby in a previous pregnancy.	Midwife-managed intervention	High risk pregnancy	Intervention additional to routine care to support and educate high-risk pregnant women and their partners subsequent to the death of a baby in a previous pregnancy.
Coates and Foureur^34^ 2019	To explore the role and competence of midwives in delivering mental healthcare.	Midwife-led (counseling/perinatal emotional support) intervention	Maternal mental health	Intervention aiming at women at risk for perinatal mental health issues embedded in midwifery care and performed by midwives.
Dai et al.^35^ 2024	To explore women’s and health professionals’ views on the development of a midwifery-led mHealth app intervention in antenatal care.	Midwifery-led mobile health app intervention	Public health	The (telemonitoring) intervention to enhance maternal health management by offering pregnant women midwifery care.
Dawson et al.^36^ 1999	To compare enhanced domiciliary care with conventional care among high- risk pregnant women.	Midwifery intervention	High risk pregnancy	Domiciliary midwifery support delivered by midwives in high-risk pregnancy.
de Wolff et al.^37^ 2021	To evaluate the effects of a midwife-coordinated maternity care intervention (ChroPreg) in pregnant women with CMC.	Midwife-coordinated maternity care intervention	High risk pregnancy	An intervention delivered to pregnant women with pre-existing chronic medical conditions by midwives.
Endqvist et al.^10^ 2017	To evaluate a multifaceted midwifery intervention designed to reduce second-degree tears among primiparous women.	A multifaceted midwifery intervention	Birth	Intervention to reduce second-degree tears in primiparous women.
Evans et al.^38^ 2020	To report on an intervention development utilizing the MRC framework for complex interventions.	A midwife facilitated intervention	Maternal mental health	Intervention specifically designed to support pregnant women with mild to moderate anxiety.
Evans et at.^39^ 2022	To report the development of a training program to prepare midwives and maternity support workers to facilitate the intervention.	A midwife-led intervention	Maternal mental health	Intervention specifically designed to support pregnant women with mild to moderate anxiety.
Evans et al.^40^ 2022b	The RAPID intervention to provide suitable support for women with mild-moderate anxiety during pregnancy	A midwife facilitated intervention	Maternal mental health	Intervention specifically designed to support pregnant women with mild to moderate anxiety.
Fenwick et al.^41^ 2011	To evaluate of the effectiveness of a midwifery-led counselling intervention.	A midwife-led counselling intervention	Maternal mental health	An intervention promoting resilience in mothers’ emotions (PRIME), on anxiety and depression in childbearing women.
Fenwick et al.^42^ 2013	To test the efficacy of a psycho-education counselling intervention offered by midwives to address women’s fear of childbirth.	A midwife-led psycho-education intervention	Maternal mental health	BELIEF: an intervention for reducing women’s fear during pregnancy.
Fenwick et al.^43^ 2015	To review women’s current expectations and feelings around fear of childbirth, and provide a framework for women to identify and work through distressing elements of childbirth.	A midwife psycho-education intervention	Maternal mental health	BELIEF: an intervention for reducing women’s fear during pregnancy.
Fernandez et al.^44^ 2019	To test if a model of care combining continuity of midwife care with rapid referral to an obstetric clinic improves experience and outcomes for women at risk of preterm birth.	Midwifery continuity of care intervention	Organization of care	Continuity of care is defined as delivering care that acknowledges that a woman’s health needs are related to events and should be managed over time.
Firouzan et al.^45^ 2020	To investigate the effectiveness of a psycho-educational intervention by midwives on decreasing childbirth fear and self-efficacy among first-time pregnant women who were afraid of giving birth.	Midwife-led psycho-educational intervention	Maternal mental health	An intervention delivered by midwives to reduce fear of childbirth.
Gamble et al.^46^ 2005	To assess a midwife-led brief counseling intervention post-partum.	A midwife-led brief counseling intervention	Maternal mental health	An intervention where midwives counsel postpartum women at risk of psychological trauma symptom.
Gamble et al.^47^ 2017	To explore organizational factors that may impact on the successful application of the midwife psycho-education intervention.	Midwife psycho-education intervention	Maternal mental health	An intervention delivered by midwives to reduce fear of childbirth.
George et al.^9^ 2018	To access effectiveness of midwifery-initiated oral health dental service program and if it improves the uptake of dental service, etc.	Midwifery intervention Midwifery initiated oral health-dental service	Public health	An intervention initiated by midwives to promote oral hygiene during pregnancy.
Gonzalez-Plaza et al.^48^ 2021	To evaluate the effectiveness of a complex digital health intervention, using a smartband and app with midwife counseling, on gestational weight gain (GWG) and physical activity (PA) in women who are pregnant and have obesity.	Midwife counselling intervention	Public health	A complex digital health intervention, using a smartband and app with midwife counseling, on GWG and physical activity (PA) in women who are pregnant and have obesity.
Gu et al.^49^ 2021	To develop and validate a midwifery-led task list in the task-shifting context.	Midwifery-led task shifting interventions	Professional role	Midwife-led services for Chinese pregnant women by shifting tasks in maternity care towards midwives.
Heins et al.^50^ 1990	To test if women at increased risk for LBW had lower rate of LBW in a nurse-midwife intervention group than in a standard care group, offered by an obstetrician.	Nurse-midwifery intervention	High risk pregnancy	Care given by a nurse-midwife (rarely a nurse) to women with a high risk for low birth weight.
Hodnett et al.^51^ 2008	To assess if a complex nursing and midwifery intervention increases the likelihood of spontaneous vaginal birth and improves maternal and neonatal outcomes.	Complex nursing and midwifery intervention to support normality in birth	Birth	Nursing or midwifery care or a minimum of one hour of care by a nurse or midwife trained in structured care when entering the hospital intrapartum.
Homer et al.^52^ 2013	To determine whether midwifery continuity of care for women with a previous CS increases the proportion of women who attempt vaginal birth in their current pregnancy.	The intervention: midwifery continuity of care	Organization of care	Women have a midwife caring for them during labor and birth whom they have met before and feel that they know, and this trusting relationship increases their confidence.
Huang et al.^53^ 2023	To explore the effectiveness of a midwife-led Internet + continuous midwifery service model for women with high-risk pregnancy.	Midwifery service intervention Midwifery intervention	Organization of care	The intervention offers maternal management from prenatal to postpartum, in-hospital to out-of-hospital, and offline to online.
Hulst et al.^54^ 2004	To examine the impact of women’s intended place of birth (home or hospital) and the course of pregnancy and labor when attended by midwives.	Midwife technological interventions; Midwife management interventions	Professional role	Explained by examples: 1) midwife technological interventions, e.g. sweeping of membranes; and 2) midwife management interventions, e.g. referral to obstetrician.
Jimenez et al.^55^ 2023	The intervention aims to improve mental state of pregnant women through breathing, mindfulness and muscle relaxation techniques.	A midwife-led e-health intervention	Maternal mental health	An intervention to reduce anxiety during pregnancy.
Khademioore et al.^56^ 2023	To evaluate the effectiveness of an interactive mobile health application, emphasizing continuous care and education, on FOC, self-efficacy, and childbirth mode in primiparous women.	Tele-midwifery intervention for primiparous women	Maternal mental health	The intervention is an interactive mHealth application based on education and continuous support provided by midwives on FOC, childbirth self-efficacy, and birth mode.
Khan et al.^57^ 2023	To identify and evaluate the current evidence related to targeted health and social care service interventions in high-income countries.	Midwifery models of care intervention	Organization of care	Midwifery models of care were defined as interventions with midwives or those similarly qualified as the central care providers or coordinators of care.
Kwegyir et al.^58^ 2018	The proposed liftless intervention aims to decrease lifting exposure during pregnancy among Ghanaian women.	Midwife-led 3-component liftless intervention	Public health	The intervention aims to decrease lifting exposure during pregnancy among Ghanaian women.
Lugina et al.^59^ 2001	To describe postpartum concerns of primiparas.	Nursing/midwifery intervention (to be developed)	Maternal mental health	First step towards an intervention decreasing worry and increasing confidence of mothers postpartum.
Lundgren et al.^60^ 2020	To explore if a midwifery model of woman-centered care (MiMo) was useful from the viewpoint of various health professionals.	MiMO (midwifery model of woman-centered care) intervention	Organization of care	A theoretical midwifery model of woman-centered care (MiMo) developed in a Nordic context.
Maga et al.^61^ 2023	To define a Midwifery Interventions Classification, an evidence-based, standardized taxonomy and classification of midwifery interventions.	Midwifery intervention	Professional role	Midwifery interventions were defined as elements of maternity care provided by midwives to improve and optimize the health outcomes of women, newborns, and public health of society at large.
Mannocci et al.^62^ 2022	To assess if a midwifery intervention is able to increase the maternal self-efficacy and reduce the stress level in the first months after birth.	Midwifery intervention	Maternal mental health	Intervention to increase maternal self-efficacy and reduce stress.
Maslin^63^ 2004	The aim is to highlight the importance and dilemma of definition and effectiveness of nursing and midwifery interventions.	Nursing and midwifery interventions	Professional role	Interventions that demonstrate the contribution of nursing and midwifery to the provision of cost-effective, quality health care.
McGiveron et al.^64^ 2015	To determine whether one-to-one antenatal guidance from midwives and healthy lifestyle advisors resulted in a lower gestational weight gain and prevalence of the common complications of pregnancy and labor associated with severe obesity.	A midwife-led intervention	Public health	An antenatal weight management intervention comprising a one-to-one program involving pregnant women with specialist midwives or healthy lifestyle advisors.
McNeill et al.^65^ 2012	To identify evidence of effective public health interventions from good quality systematic reviews that could be conducted by midwives.	Midwifery intervention Midwifery public health interventions	Public health	Public health interventions during pregnancy and postnatal that can be implemented by midwives. But also by care providers that have similar roles.
Meedya et al.^66^ 2010	To explore what modifiable factors positively influence breastfeeding duration to 6 months post-partum.	Midwifery intervention A midwife-provided educational intervention	Public health	First step towards an intervention that strengthens modifying factors for breastfeeding duration up to 6 months postpartum.
Meedya et al.^67^ 2014	To evaluate the effectiveness of a multiphase midwifery intervention called the ‘Milky Way’ on any breastfeeding rates until six months.	A multiphase midwifery intervention called the Milky Way	Public health	An intervention to support women who are breastfeeding.
Morlans et al.^68^ 2022	To improve the quality of continuity of care and emotional well-being in women with high-risk pregnancies.	Midwife-led continuity of care interventions	Organization of care	The main intervention was the establishment of a midwifery consultation including four antenatal visits and one in the postpartum period.
Morrell et al.^69^ 2016	To evaluate the clinical effectiveness, cost-effectiveness, acceptability and safety of ante- and postnatal interventions for pregnant and postnatal women to prevent PND.	Midwifery-led interventions	Maternal mental health	Interventions to prevent post-natal depression.
Nkowane and Ferguson^70^ 2021	To strengthen nursing and midwifery and the health workforce in general.	Midwifery interventions	Professional role	Interventions that strengthen the workforce of midwifery and improve the impact of the work of midwives.
Ogrodniczuk and Piper^71^ 2003	To offer an overview on selective and indicated measures directed at preventing postnatal depression.	Midwife(-type) intervention	Maternal mental health	Interventions provided by midwives to prevent postnatal depression.
Panda and Begley^72^ 2014	To ascertain the outcomes of labor and describe the interventions by midwives for women admitted to the antenatal ward with labor-related symptoms.	Midwifery interventions in early labor	Birth	Interventions performed by midwives when women present in the antenatal ward at the start of labor.
Perez-Martinez et al.^73^ 2019	To evaluate the frequency of visits to the hospital emergency department due to puerperal complications attended by midwives instead of obstetricians.	Midwives’ intervention	Professional role	Puerperal health education provided to women in the hospital by midwives individually on a daily basis during the clinical rounds and at discharge.
Petersen et al.^74^ 2011	To describe the timing and frequency of interventions during labor, and in addition to compare the timings of the interventions against the partogram action lines.	Interventions applying midwifery care techniques	Birth	The presence of the midwife attending the woman in labor was considered an intervention and using midwifery care techniques, e.g. vertical positioning.
Polanska et al.^75^ 2004	To evaluate the effectiveness of anti-smoking counseling in the population of pregnant women in central Poland.	The midwife-assisted smoking cessation intervention	Public health	An intervention where midwives helped pregnant women to quit smoking.
Ray and Salihu^76^ 2004	To examine maternal mortality interventions that improve management of labor and delivery through training midwives and traditional birth attendants.	Midwife and traditional birth attendant-based interventions.	Birth	Interventions performed by midwife and traditional birth attendant to improve maternal mortality focus on birth.
Rodriguez- Gallego et al.^77^ 2024	To assess the effectiveness of a midwife-led breastfeeding support group intervention on breastfeeding, postpartum depression and general self-efficacy.	Midwife-led breastfeeding support group intervention	Public health	Group interventions during the postpartum period to prevent postpartum depression.
Sigurðardóttir et al.^78^ 2023	To review birth experiences, when limited knowledge exists about appropriate interventions and feasibility of this care for women with high-risk pregnancies.	A postpartum midwifery counselling intervention	Maternal mental health	Intervention offered by midwives to assist women to process a negative birth experience.
Simpson and Catling^79^ 2016	To gain a better understanding of factors contributing to birth trauma, and the efficacy of existing interventions.	Midwifery-led intervention	Maternal mental health	Interventions that offer emotional support from midwives during the antenatal and postpartum period.
Smoke and Grace^80^ 1988	To evaluate the change in pregnancy-related knowledge by pre- and post-education and to compare the pregnancy outcomes in two adolescent groups.	Nurse-midwifery intervention	Organization of care	Care coordinated by a USA-certified nurse-midwife.
Souto et al.^81^ 2020	To map and analyze midwife interventions to reduce fear of childbirth in pregnant women.	Midwife intervention	Maternal mental health	Intervention to reduce fear of childbirth in pregnant women. Originally, it was called midwifery intervention. In an erratum (2021) this was changed to midwife intervention.
Souto et al.^82^ 2022	To identify midwives’ interventions for reducing fear of childbirth in pregnant women and to examine their characteristics.	Midwives’ intervention	Maternal mental health	All interventions that included a midwife or team of midwives to reduce fear of childbirth.
Spindler et al.^83^ 2018	To assess changes in skill and knowledge on the use of evidence-based practices associated with quality of maternal and neonatal care during a nurse midwife mentoring intervention	A nurse midwife mentoring intervention	Professional role	No clear definition → debriefing after births to improve skills and knowledge of midwives.
Swann and Davies^84^ 2012	-	Midwifery intervention	Birth	No explicit definition.
Taylor Miller et al.^85^ 2021	To investigate the effectiveness of early psychological interventions in reducing or preventing post-traumatic stress symptoms and post-traumatic stress disorder in post-partum women with a traumatic birth.	Midwifery-led intervention Midwifery-led brief counselling intervention	Maternal mental health	Intervention targeting post-traumatic stress disorder performed by midwives.
Toohill et al.^86^ 2017	To determine the cost-effectiveness of a midwife-led psycho-education intervention for women fearful of birth.	A midwife-led psycho-education intervention	Maternal mental health	No definition. See: Fenwick et al.^42^.
Toohill et al.^87^ 2019	To determine whether healthcare use and access to continuity models are equal across different indicators of socioeconomic status for women who are fearful of birth.	A midwife-led psycho-education intervention	Maternal mental health	No definition. See: Fenwick et al.^42^.
Truva et al.^88^ 2021	To investigate the effect of a lactation educational intervention by a midwife on increasing breastfeeding rates in women.	Midwifery intervention program	Public health	No definition. A personalized intervention program on increasing breastfeeding rates provided by midwives.
Türkmen and Oran^89^ 2021	To determine the effects of sacral massage and heat application on labor pain and comfort level in pregnant women.	Midwifery intervention	Birth	No definition. The intervention used thermoforming and massage given by midwives.
Türkmen et al.^90^ 2023	To determine the effects of ice massage applied to the SP6 acupressure point during labor pain, labor comfort, labor duration, and anxiety.	Midwifery intervention	Birth	No definition. The intervention used rotational ice massage on pregnant women given by midwives
Turkstra et al.^91^ 2017	To test an antenatal psycho-education intervention by midwives in reducing women’s childbirth fear.	A brief antenatal midwifery psycho-education intervention	Maternal mental health	The intervention addressing women’s expectations and feelings around fear of childbirth.
Wallace et al.^92^ 2006	To determine whether postnatal ‘hands off’ care by midwives on positioning and attachment of the newborn baby improves breast-feeding duration.	Midwifery intervention	Public health	No definition. The protocol required that the intervention a ‘hands off’ approach to care at first feed was delivered by midwives.
Wang et al.^93^ 2021	To evaluate the effectiveness of nurses and midwives-led psychological interventions on the perinatal depressive symptoms.	Midwives-led psychological intervention	Maternal mental health	Interventions provided by midwives on depressive symptoms in perinatal women.
Wang et al.^94^ 2023	To examine the effects of a midwife-led weight management program on improving appropriate gestational weight gain, health literacy, experience of antenatal care, and maternal and neonatal outcomes.	A midwife-led weight management program	Public health	A midwife-led weight management program that facilitates appropriate gestational weight gain for pregnant women, enhances health literacy, and promotes experience of antenatal care.
Warren et al.^95^ 2017	To assess the feasibility and acceptability of the ‘Eat Well Keep Active’ intervention program designed to promote healthy eating and physical activity in pregnancy.	A brief midwife led intervention	Public health	An ‘Eat Well Keep Active’ intervention program to facilitate healthful dietary and physical activity behaviors in pregnant women.
Wei et al.^96^ 2021	To investigate the effect of midwife intervention coupled with acupressure on the vaginal delivery rate and negative emotion in women with scarred uterus re-pregnancy.	Midwife intervention nursing mode during birth (combined with acupressure).	Birth	A mode dominated by midwives is more humanized and targeted to promote vaginal birth versus routine obstetric care.
Wilkinson et al.^97^ 2016	To develop a brief intervention for antenatal anxiety, with a focus on embedding the delivery of the treatment within routine antenatal care.	Midwife-led group intervention	Maternal mental health	An intervention tailored specifically for use with pregnant women experiencing antenatal anxiety, based on the principles of Cognitive Behavioral Therapy (CBT), led by midwives.

Further details in regard to the references are given in the Supplementary file.

For step two, the critical analysis, we included six publications where midwives were involved in the development and delivery of the intervention and that described at least 7 of the 8 components of the ICM Philosophy of Midwifery Care^10,30,42,46,52,60^. We excluded systematic reviews (n=15) as they did not offer a description of each separate intervention (Supplementary file Parts 4 and 6).

The actual term of midwifery intervention was used in 14 publications^9,19,22,36,62,63,66,67,70,84,88-90,92^ and another 31 times the term was used in combination with other words indicating the content of the intervention, such as caseload, public health, or as midwifery-led^9,10,18,21-25,31,32,35,44,49-52,56,57,59,60,63,65,67,69,72,74,78-80,85,91^. Similarly, six publications used the term midwife (midwives) intervention^71,73,81,82,96^, also combining this term 36 times with other words, such as counselling, psychoeducation, or as midwife-led^20,26-30,33,34,37-43,45-48,53-55,58,64,66,68,75-77,83,86,87,93-95,97^. Some authors used both midwifery and midwife in combinations with intervention in their publication. The content orientation of the interventions in the 83 publications was on: Maternal mental health (n=31)^18,22,26,29,34,38-43,45-47,56,57,59,62,69,71,78,79,81,82,85-87,91,93,97^, Public health (n=17)^9,20,27,32,35,48,58,64-67,75,77,88,92,94,95^, Birth (n=9)^10,51,72,74,76,84,89,90,96^, High risk pregnancy (n=4)^33,36,37,50^, Midwives’ professional role (n=12)^19,21,25,28,31,49,54,61,63,70,73,83^, and Organization of care (n=10)^23,24,30,44,52,53,57,60,68,80^. Overall, the publications presented 48 separate interventions (Supplementary file Parts 3 and 5).

### Evaluation of the concept as used in scientific literature (step one)

The concept was evaluated using the criteria for the evaluation of a concept analysis^16^, including the definition, defining attributes, boundaries, antecedents and consequences.

*Definitions*

Definitions of midwifery/midwife intervention given in the publications primarily focused on the content orientation of the intervention. If midwife was mentioned in the definition, it was almost always linked to the task midwives have in performing the intervention^19,21,22,25,29,30,34,36,37,45-52,54,56-58,61,63-64,70-76,78-80,82,83,85,88-90,93,94,96,97^ or to midwifery in general^23,28,60,68,70^. Rarely, the definition included a further explanation of midwife or midwifery. Only in the publications by Souto et al.^81,82^ did the authors offer a further explanation of what they implied with a midwife ‘*as a responsible and accountable professional who works in partnership with women to give the necessary support, care and advice …’* ^81^ and the extra value midwifery can bring to an intervention (Supplementary file Part 2).

Concluding, the midwifery component in the concept of midwifery/midwife intervention is not clearly defined and open to interpretation.

*Defining attributes*

For all 48 interventions, midwives were the intended performers of the intervention; for some also nurses^21,25,51,62,63^ or other (midwife-like) health professionals, such as midwife support workers, lifestyle advisors^38,62,64,65^, were suggested as possible performers. In 20 out of 48 interventions (42%), it was clear that midwives had some role in the development^10,20,24,28,32,33,35,38,44,46,60,64,67,73,78,80,89,92,94,95^; midwives as initiator of interventions or a balanced participation of midwife members in the development team seemed even more limited. Twenty-five of the 83 publications mentioned aspects distinctive for midwifery care as a distinguished part of the intervention, such as: relationship between midwife and woman^18,26,40,44,52,60,79^, non-invasive^22^, continuity of care^24,30,40,44,57,72^, known midwife^23,37,44,52,78,82^, holistic approach^32,57,81^, physiology of birth as premise^10,35,49,96^, recognizing women’s human rights^81^ and empowerment of women^55,88,92,96^. These interventions most often had midwifery rather than midwife in the naming of the intervention, although this was not conclusive (Supplementary file Part 2).

Concluding, the only shared defining attribute for the concept of midwifery/midwife intervention that was found in our literature exploration was: Intervention performed by a midwife.

*Boundaries*

Few publications described the boundaries of midwifery interventions compared to other interventions. Some authors contrasted their midwifery intervention with other interventions, named as invasive interventions^22^, obstetric interventions^49,54^, medical interventions^74,84^ or as interventions without a midwife being involved^29,34^. Others contrasted it with care not including a midwifery orientation of the intervention, for example care without continuity^30,44,52^. However, explanations about how a midwifery intervention differed from those other interventions was limited (Supplementary file Part 2).

These descriptions of boundaries indicate that a midwife should be involved in performing the intervention to make it a midwifery intervention but does not offer further insight into what features make it specifically midwifery, distinguished from other interventions in maternity care.

*Antecedents*

The antecedent for the concept of midwifery intervention mentioned in all publications was the availability of a practicing midwife with midwifery education. Often complemented by a short additional training in the specific orientation of the intervention^9,10,20,27-29,31,32,36-38,46,48,58,60,62,66,67,77,78,92,94,97^. Occasionally, the intervention required organizational changes on practice level, such as midwives working in a small team^24,30^, midwives working in a family health center or doing visits at home^25,75^, midwives seeing the woman throughout care (Supplementary file Part 2, ref: 28,35), creating clarity about midwifery tasks/role (Supplementary file Part 2, ref. 34,36,40,53), or even broader system changes including legal and governance aspects^19,21,34^ (Supplementary file Part 2).

The general requirement for performing a midwifery intervention was a midwifery education.

*Consequences*

Most interventions aimed for outcomes related to the orientation of the intervention, for example interventions oriented on psychological wellbeing of women aimed for better mental health, less fear of childbirth or less birth trauma. Some publications mentioned more ambitious goals, such as universal health coverage, realizing the Sustainable Developments Goals, improved healthcare quality or population health^21,28,30,70^. Only a few mentioned consequences related to the midwifery philosophy of care^12^, including women’s positive experience of childbirth, being well-informed, sense of control during childbirth, empowerment, or physiological childbirth^24,33,49,51,52,57,73,84,86^ (Supplementary file Part 2).

### Maturity of the concept

Morse^16^ argues that ‘Concepts do not dichotomously just exist or not exist. Rather concepts emerge and are tentatively introduced to the scientific community’. Our evaluation of the concept midwifery intervention shows that the concept has clearly found its way to the scientific community of maternity care with 83 scientific publications mentioning the concept. However, it is also clear that it still lacks maturity on what midwifery implies in the context of interventions while aiming to improve specific or general health of mothers and newborns. The concept lacks clarity and consensus about the definition across all publications, shows limitations in the identified attributes, antecedents and consequences, and has no known boundaries.

Based on our concept evaluation of midwifery intervention, the concept is currently immature.

### Critical analysis of the concept midwifery intervention (step two)

Many publications used the term midwifery intervention or related terms to specify that the intervention was performed by a midwife without further indications of what this implied or aimed for and without midwives always being involved in the development of the intervention^9,19,21,25,27,31,48,50,54,56,58,62-64,72,83^.

For our next step, the critical analysis of the included publications, we used several questions (listed earlier), including a question on how the description of the intervention related to the ICM Philosophy of Midwifery Care (Supplementary file Part 4). Based on these questions, we identified several publications that gave the midwifery component in the concept of midwifery intervention a deeper consideration. We found six articles that describe an intervention that strongly relates to the components of the ICM Philosophy of Midwifery Care and where midwives appear to have had a distinguished role in the development of the intervention^10,30,42,46,52,60^. These descriptions can be seen as exemplary for further development of the concept of midwifery intervention. They offered a deeper insight in what a midwifery intervention should imply, contributing to a growing maturity of the concept (Supplementary file Part 6).

Based on this analysis, we propose to use the term midwifery intervention for interventions that aim at promoting physiology of childbirth in a safe context and recognizing that pregnancy and childbearing are a profound experience for women. The intervention should strengthen women’s and newborns’ health with a salutogenic approach, contributing to women’s self-confidence and respecting her right of self-determination. The intervention should be culturally sensitive and informed by evidence.

## DISCUSSION

Our search and analysis show that the concept of midwifery intervention is not well defined and lacks a harmonious understanding. We see a mix of terms, such as midwifery intervention, midwife intervention, nurse-midwife intervention, midwife-led intervention. The actual term midwifery intervention is used in only 16 of 83 publications, and midwife intervention in another six publications. More importantly, the midwifery component of the intervention is poorly defined and mostly refers to the instrumental role midwives can play in performing the intervention. Critical analysis of the publications shows that the descriptions of only six out of 48 interventions strongly relate to the components of the ICM Philosophy of Midwifery Care. This undermines a wider understanding of what midwifery is about and the added value midwives have for women and newborns, as well as the positing of midwives in healthcare.

Often the publications do not describe if and how midwives were involved in the development of the intervention. Only 20 out of the 48 interventions (42%) describe that a midwife had some role in the development. Midwives as initiator of interventions or a balanced participation of midwife members in the development team seems even more limited. Similarly, the participation of women in the development of interventions was also limited. If midwives are not involved in the development of interventions they have to perform in daily practice, the incorporation of the ICM midwifery philosophy in the intervention will not succeed^12^. This might limit woman-centered care promoting physiological birth, a holistic approach and partnership with women. To enhance successful implementation an active alignment with providers’ daily practice throughout the development of an intervention is crucial and recommended. This requires active involvement of all stakeholders from the initiation to implementation of an intervention^98,99^.

The content orientation of the interventions in the selected publications varies with interventions for maternal mental health receiving most attention. This includes interventions to address pre- or postpartum psychological disorders, fear of childbirth or negative/traumatic birth experiences^18,22,26,29,34,38-43,45-47,55,56,59,62,69,71,78,79,81,82,85-87,91,93,97^. Mental health is a relatively new research area for midwives, and one could speculate that development of midwifery interventions in this context is caused by increased risk culture where the rate of normal births has decreased in the past decades^100^. We note that few of the midwifery interventions in our study related to birth itself, such as preparing parents for birth or aiming to promote physiological childbirth, while this is the first component of the ICM philosophy of midwifery care^12^. Some publications discuss the organization of care, for example continuity of care, case load midwifery or care coordination by a midwife^23,24,30,44,52,53,57,60,68,80^. The care that is offered in these interventions can be seen as a way to increase positive childbirth experiences or the probability of spontaneous vaginal birth. An example is the study by Homer et al.^52^ where the midwifery intervention consists of offering continuity of care by a midwife to women with a previous cesarean section to promote women’s choice for a vaginal birth. Similarly, the midwifery model for woman-centered care of Lundgren et al.^60^ highlights an intervention where the midwife is with the woman, forms a reciprocal relationship, and creates a birthing atmosphere.

Very few of the studies have the aim to support and promote normality of the perinatal period. Most are designed to address risk or prevent the occurrence of certain negative physical or mental outcomes. This aligns with the overall tendency in maternity care to approach childbirth as a risky event, physically and increasingly also mentally^101^. However, the fundament of the midwifery philosophy is to recognize and support women’s strengths, and work in partnership with women from a salutogenic perspective, not regarding pregnancy and birth as a disease^102^. Salutogenesis as a concept, articulated by Antonovsky^103^, brings a scholarly focus to studying the origins of health, instead of the origins of disease. The focus of a salutogenic approach to care is on supporting individuals and populations to increase control over and improve their health and resilience^104^, strengthening what makes them healthy and not merely preventing what makes them sick. This is a crucial component of midwifery interventions.

In line with the ICM philosophy of care, midwives are reluctant about interventions, but still do intervene. Midwives may hesitate about interventions; however, many recognize their value when clinically indicated and aligned with women’s choices. Intervening in a natural process can be disruptive and may cause a cascade of interventions^105^. Still, nature is not always kind and using interventions can save lives. As Miller et al.^2^ argue in the ground breaking publication ‘Beyond too much, too soon and too little, too late’, ‘the right amount of care needs to be offered at the right time, and delivered in a manner that respects, protects, and promotes human rights’. This contributes to effective care that prevents under- and over- medicalization. True midwifery interventions, such as ‘watchful attendance’ where a midwife is present at birth and gives support tailored to the woman’s needs without disrupting the process^106^, meet these requirements and can promote the physiology of childbirth while limiting medical interventions.

### Complexity of concepts

The robust development of concepts is a vital component in advancing the knowledge base of midwifery theory and practice^14^. One problem with our findings is the mix of terminology without a clear meaning. A definition of midwifery intervention fitting the midwifery philosophy of care is significant to make midwifery care visible in research and in the documentation of procedures performed by midwives in the clinical context, in record keeping both regarding communication between professionals, improved standards of care; audits and clinical reviews; and research and education.

### Strengths and limitations

Strengths of this study are the thorough exploration of the literature and the use of the ICM philosophy of midwifery care as a reference for the exploration of what is actually meant by midwifery intervention. However, this is limited by the fact that we had to work with the descriptions of the development, content and provision of the interventions in the selected articles. These were not always very detailed. Therefore, our study must be regarded as a first explorative step to gain more clarity on what midwifery intervention actually means and what contribution it can make to the health of women and their infants during the perinatal period.

Next steps should involve further exploration and validation of the proposed definition of midwifery intervention involving both midwives and women, e.g. through a Delphi study. With this, we can start evaluating the effectiveness of midwifery interventions that actually meet the proposed description.

## CONCLUSIONS

This study shows that there is a gap in knowledge and understanding about the midwifery profession when it comes to what midwives contribute in clinical practice. Too often midwives are considered as merely performers of certain tasks or interventions. The term midwifery intervention should be used for interventions where midwives (and women) are involved in designing the actual intervention. Midwifery interventions should focus on an approach that links closely to a philosophy of midwifery care aiming at promote physiology of childbirth in a safe context and recognizing that pregnancy and childbearing are a profound experience for women. The intervention should strengthen women’s and newborns’ health with a salutogenic approach, contributing to women’s self-confidence and respecting her right of self-determination. The intervention should be culturally sensitive and informed by evidence. Clarity in establishing this concept will be useful for clinical practice, research and education.

## Supplementary Material



## Data Availability

The data supporting this research can be found within the article and in the Supplementary file.

## References

[1] Kennedy HP, Lowe NK. Science and midwifery: paradigms and paradoxes. J Midwifery Womens Health. 2001;46(2):91-97. doi:10.1016/s1526-9523(01)00101-5 11370696

[2] Miller S, Abalos E, Chamillard M, et al. Beyond too little, too late and too much, too soon: a pathway towards evidence-based, respectful maternity care worldwide. Lancet. 2016;388(10056):2176-2192. doi:10.1016/S0140-6736(16)31472-6 27642019

[3] Meaning of intervention in English. Cambridge Dictionary. Accessed January 4, 2026. https://dictionary.cambridge.org/dictionary/english/intervention

[4] McKenzie PJ, Oliphant T. Informing evidence: claimsmaking in midwives’ and clients’ talk about interventions. Qual Health Res. 2010;20(1):29-41. doi:10.1177/1049732309355591 19940086

[5] Souter V, Nethery E, Kopas ML, Wurz H, Sitcov K, Caughey AB. Comparison of midwifery and obstetric care in low-risk hospital births. Obstet Gynecol. 2019;134(5):1056-1065. doi:10.1097/AOG.0000000000003521 31599830

[6] Toohill J, Fenwick J, Gamble J, et al. A randomized controlled trial of a psycho-education intervention by midwives in reducing childbirth fear in pregnant women. Birth. 2014;41(4):384-394. doi:10.1111/birt.12136 25303111 PMC4257571

[7] Brownson RC, Shelton RC, Geng EH, Glasgow RE. Revisiting concepts of evidence in implementation science. Implement Sci. 2022;17(1):26. doi:10.1186/s13012-022-01201-y 35413917 PMC9004065

[8] Renfrew MJ, McFadden A, Bastos MH, et al. Midwifery and quality care: findings from a new evidence-informed framework for maternal and newborn care. Lancet. 2014;384(9948):1129-1145. doi:10.1016/S0140-6736(14)60789-3 24965816

[9] George A, Dahlen HG, Blinkhorn A, et al. Evaluation of a midwifery initiated oral health-dental service program to improve oral health and birth outcomes for pregnant women: a multi-centre randomised controlled trial. Int J Nurs Stud. 2018;82:49-57. doi:10.1016/j.ijnurstu.2018.03.006 29605753

[10] Edqvist M, Hildingsson I, Mollberg M, Lundgren I, Lindgren H. Midwives’ management during the second stage of labor in relation to second-degree tears-an experimental study. Birth. 2017;44(1):86-94. doi:10.1111/birt.12267 27859542 PMC5324579

[11] International Confederation of Midwives. Definition of Midwifery. Updated July 9, 2025. Accessed January 4, 2026. https://www.internationalmidwives.org/assets/files/definitions-files/2018/06/eng-definition_midwifery.pdf

[12] International Confederation of Midwives. Philosophy and Model of Midwifery Care. Updated July 8, 2025. Accessed January 4, 2026. https://www.internationalmidwives.org/assets/files/definitions-files/2018/06/eng-philosophy-and-model-of-midwifery-care.pdf

[13] Maimburg RD, Blix E. Perspectives on childbearing. In: Lundgren I, Blix E, Gottfredsdóttir H, Wikberg A, Aagaard Nohr E, eds. Theories and Perspectives for Midwifery: A nordic view. Studentlitteratur; 2022:chap 7.

[14] Beecher C, Devane D, White M, Greene R, Dowling M. Concept development in nursing and midwifery: an overview of methodological approaches. Int J Nurs Pract. 2019;25(1):e12702. doi:10.1111/ijn.12702 30338594

[15] Morse JM, Hupcey JE, Mitcham C, Lenz ER. Concept analysis in nursing research: a critical appraisal. Sch Inq Nurs Pract. 1996;10(3):253-277. 9009821

[16] Morse JM, Mitcham C, Hupcey JE, Tasón MC. Criteria for concept evaluation. J Adv Nurs. 1996;24(2):385-390. doi:10.1046/j.1365-2648.1996.18022.x 8858445

[17] Page MJ, McKenzie JE, Bossuyt PM, et al. The PRISMA 2020 statement: an updated guideline for reporting systematic reviews. Syst Rev. 2021;10(1):89. doi:10.1186/s13643-021-01626-4 33781348 PMC8008539

[18] Hajarian Abhari Z, Karimi FZ, Taghizdeh Z, Mazloum SR, Asghari Nekah SM. Effects of counseling based on Gamble’s approach on psychological birth trauma in primiparous women: a randomized clinical trial. J Matern Fetal Neonatal Med. 2022;35(4):668-676. doi:10.1080/14767058.2020.1730799 32089025

[19] Abou Malham S, Hatem M, Leduc N. A case study evaluation of an intervention aiming to strengthen the midwifery professional role in Morocco: anticipated barriers to reaching outcomes. J Multidiscip Healthc. 2015;8:419-432. doi:10.2147/JMDH.S86920 26445547 PMC4590574

[20] Adams SH, Gregorich SE, Rising SS, Hutchison M, Chung LH. Integrating a nurse-midwife-led oral health intervention into centeringpregnancy prenatal care: results of a pilot study. J Midwifery Womens Health. 2017;62(4):463-469. doi:10.1111/jmwh.12613 28686808 PMC5909701

[21] Ajuebor O, McCarthy C, Li Y, Al-Blooshi SM, Makhanya N, Cometto G. Are the Global Strategic Directions for Strengthening Nursing and Midwifery 2016-2020 being implemented in countries? Findings from a cross-sectional analysis. Hum Resour Health. 2019;17(1):54. doi:10.1186/s12960-019-0392-2 31300058 PMC6626395

[22] Alderdice F, McNeill J, Lynn F. A systematic review of systematic reviews of interventions to improve maternal mental health and well-being. Midwifery. 2013;29(4):389-399. doi:10.1016/j.midw.2012.05.010 22882967

[23] Allen J, Kildea S, Stapleton H. How optimal caseload midwifery can modify predictors for preterm birth in young women: Integrated findings from a mixed methods study. Midwifery. 2016;41:30-38. doi:10.1016/j.midw.2016.07.012 27498186

[24] Allen J, Kildea S, Hartz DL, Tracy M, Tracy S. The motivation and capacity to go ‘above and beyond’: qualitative analysis of free-text survey responses in the M@NGO randomised controlled trial of caseload midwifery. Midwifery. 2017;50:148-156. doi:10.1016/j.midw.2017.03.012 28458123

[25] Altiner M, Secginli S, Mathiason MA, Monsen KA. Method development for describing content of multitasked interventions using the Omaha System. Res Theory Nurs Pract. 2019;33(2):147-168. doi:10.1891/1541-6577.33.2.147 31123160

[26] Asadzadeh L, Jafari E, Kharaghani R, Taremian F. Effectiveness of midwife-led brief counseling intervention on post-traumatic stress disorder, depression, and anxiety symptoms of women experiencing a traumatic childbirth: a randomized controlled trial. BMC Pregnancy Childbirth. 2020;20(1):142. doi:10.1186/s12884-020-2826-1 32138707 PMC7059371

[27] Bick D, Bishop J, Coleman T, et al. Antenatal preventative pelvic floor muscle exercise intervention led by midwives to reduce postnatal urinary incontinence (APPEAL): protocol for a feasibility and pilot cluster randomised controlled trial. Pilot Feasibility Stud. 2022;8(1):231. doi:10.1186/s40814-022-01185-y 36273227 PMC9588215

[28] Blomgren J, Wells MB, Erlandsson K, Amongin D, Kabiri L, Lindgren H. Putting co-creation into practice: lessons learned from developing a midwife-led quality improvement intervention. Glob Health Action. 2023;16(1):2275866. doi:10.1080/16549716.2023.2275866 37930253 PMC10629418

[29] Borg Cunen N, McNeill J, Murray K. A systematic review of midwife-led interventions to address post partum post-traumatic stress. Midwifery. 2014;30(2):170-184. doi:10.1016/j.midw.2013.09.003 24238899

[30] Borges, S. Cystic fibrosis and caseload Midwifery. Br J Midwifery. 2021;29(12):712-717. doi:10.12968/bjom.2021.29.12.712

[31] Borneskog C, Engström G, Islam N, et al. Midwife Educators’ perceptions of the efficacy of the Objective Structured clinical assessment of life-saving interventions - a qualitative interview study in Bangladesh. Sex Reprod Healthc. 2023;37:100861. doi:10.1016/j.srhc.2023.100861 37267736

[32] Bryce A, Butler C, Gnich W, Sheehy C, Tappin DM. CATCH: development of a home-based midwifery intervention to support young pregnant smokers to quit. Midwifery. 2009;25(5):473-482. doi:10.1016/j.midw.2007.10.006 18280015

[33] Caelli K, Downie J, Letendre A. Parents’ experiences of midwife-managed care following the loss of a baby in a previous pregnancy. J Adv Nurs. 2002;39(2):127-136. doi:10.1046/j.1365-2648.2002.02252.x 12100656

[34] Coates D, Foureur M. The role and competence of midwives in supporting women with mental health concerns during the perinatal period: a scoping review. Health Soc Care Community. 2019;27(4):e389-e405. doi:10.1111/hsc.12740 30900371

[35] Dai Y, Min H, Sun L, Wang X, Zhu C, Gu C. Assessing women’s and health professionals’ views on developing a midwifery-led mobile health app intervention in pregnancy: a descriptive qualitative study. J Adv Nurs. 2024;80(10):4259-4271. doi:10.1111/jan.16086 38332497

[36] Dawson A, Cohen D, Candelier C, et al. Domiciliary midwifery support in high-risk pregnancy incorporating telephonic fetal heart rate monitoring: a health technology randomized assessment. J Telemed Telecare. 1999;5(4):220-230. doi:10.1258/1357633991933756 10829372

[37] de Wolff MG, Midtgaard J, Johansen M, et al. Effects of a Midwife-Coordinated Maternity Care Intervention (ChroPreg) vs. standard care in pregnant women with chronic medical conditions: results from a randomized controlled trial. Int J Environ Res Public Health. 2021;18(15):7875. doi:10.3390/ijerph18157875 34360168 PMC8345548

[38] Evans K, Spiby H, Morrell CJ. Developing a complex intervention to support pregnant women with mild to moderate anxiety: application of the Medical Research Council framework. BMC Pregnancy Childbirth. 2020;20(1):777. doi:10.1186/s12884-020-03469-8 33317463 PMC7734709

[39] Evans K, Moya H, Lambert M, Spiby H. Developing a training programme for midwives and maternity support workers facilitating a novel intervention to support women with anxiety in pregnancy. BMC Pregnancy Childbirth. 2022;22(1):662. doi:10.1186/s12884-022-04996-2 36008799 PMC9403963

[40] Evans K, Spiby H, Slade M, Jomeen J, Beckhelling J. RAPID-2 study protocol: a cluster randomised feasibility trial of a midwife facilitated intervention for pregnant women with symptoms of mild to moderate anxiety. BMJ Open. 2022;12(10):e064659. doi:10.1136/bmjopen-2022-064659 PMC9615978 36288833

[41] Fenwick J, Gamble J, Creedy D, Barclay L. Women´s experiences of the PRIME midwifery counselling intervention: promoting resilience in mothers emotions. Women and Birth. 2011;24(suppl 1):S11-S12. doi:10.1016/j.wombi.2011.07.051

[42] Fenwick J, Gamble J, Creedy DK, et al. Study protocol for reducing childbirth fear: a midwife-led psycho-education intervention. BMC Pregnancy Childbirth. 2013;13:190. doi:10.1186/1471-2393-13-190 24139191 PMC3854500

[43] Fenwick J, Toohill J, Gamble J, et al. Effects of a midwife psycho-education intervention to reduce childbirth fear on women’s birth outcomes and postpartum psychological wellbeing. BMC Pregnancy Childbirth. 2015;15:284. doi:10.1186/s12884-015-0721-y 26518597 PMC4628230

[44] Fernandez Turienzo C, Bick D, Bollard M, et al. POPPIE: protocol for a randomised controlled pilot trial of continuity of midwifery care for women at increased risk of preterm birth. Trials. 2019;20(1):271. doi:10.1186/s13063-019-3352-1 31088505 PMC6518651

[45] Firouzan L, Kharaghani R, Zenoozian S, Moloodi R, Jafari E. The effect of midwifery led counseling based on Gamble’s approach on childbirth fear and self-efficacy in nulligravida women. BMC Pregnancy Childbirth. 2020;20(1):522. doi:10.1186/s12884-020-03230-1 32907547 PMC7488155

[46] Gamble J, Creedy D, Moyle W, Webster J, McAllister M, Dickson P. Effectiveness of a counseling intervention after a traumatic childbirth: a randomized controlled trial. Birth. 2005;32(1):11-19. doi:10.1111/j.0730-7659.2005.00340.x 15725200

[47] Gamble J, Toohill J, Slavin V, Creedy DK, Fenwick J. Identifying barriers and enablers as a first step in the implementation of a midwife-led psychoeducation counseling framework for women fearful of birth. Int J Childbirth. 2017;7(3):152-168. doi:10.1891/2156-5287.7.3.152

[48] Gonzalez-Plaza E, Bellart J, Arranz Á, Luján-Barroso L, Crespo Mirasol E, Seguranyes G. Effectiveness of a step counter smartband and midwife counseling intervention on gestational weight gain and physical activity in pregnant women with obesity (pas and pes study): randomized controlled trial. JMIR Mhealth Uhealth. 2022;10(2):e28886. doi:10.2196/28886 35166684 PMC8889480

[49] Gu C, Lindgren H, Wang X, et al. Developing a midwifery service task list for Chinese midwives in the task-shifting context: a Delphi study. BMJ Open. 2021;11(7):e044792. doi:10.1136/bmjopen-2020-044792 PMC8286777 34266838

[50] Heins HC Jr, Nance NW, McCarthy BJ, Efird CM. A randomized trial of nurse-midwifery prenatal care to reduce low birth weight. Obstet Gynecol. 1990;75(3):341-345. 2406656

[51] Hodnett ED, Stremler R, Willan AR, et al. Effect on birth outcomes of a formalised approach to care in hospital labour assessment units: international, randomised controlled trial. BMJ. 2008;337:a1021. doi:10.1136/bmj.a1021 18755762 PMC2526182

[52] Homer CS, Besley K, Bell J, et al. Does continuity of care impact decision making in the next birth after a caesarean section (VBAC)? A randomised controlled trial. BMC Pregnancy Childbirth. 2013;13:140. doi:10.1186/1471-2393-13-140 23819882 PMC3717054

[53] Huang CJ, Han W, Huang CQ. Effect of Internet + continuous midwifery service model on psychological mood and pregnancy outcomes for women with high-risk pregnancies. World J Psychiatry. 2023;13(11):862-871. doi:10.5498/wjp.v13.i11.862 38073899 PMC10701212

[54] van der Hulst LA, van Teijlingen ER, Bonsel GJ, Eskes M, Bleker OP. Does a pregnant woman’s intended place of birth influence her attitudes toward and occurrence of obstetric interventions? Birth. 2004;31(1):28-33. doi:10.1111/j.0730-7659.2004.0271.x 15015990

[55] Jimenez-Barragan M, Del Pino Gutierrez A, Garcia JC, et al. Study protocol for improving mental health during pregnancy: a randomized controlled low-intensity m-health intervention by midwives at primary care centers. BMC Nurs. 2023;22(1):309. doi:10.1186/s12912-023-01440-4 37674184 PMC10483870

[56] Khademioore S, Ebrahimi E, Khosravi A, Movahedi S. The effect of an mHealth application based on continuous support and education on fear of childbirth, self-efficacy, and birth mode in primiparous women: a randomized controlled trial. PLoS One. 2023;18(11):e0293815. doi:10.1371/journal.pone.0293815 37910495 PMC10619799

[57] Khan Z, Vowles Z, Fernandez Turienzo C, et al. Targeted health and social care interventions for women and infants who are disproportionately impacted by health inequalities in high-income countries: a systematic review. Int J Equity Health. 2023;22(1):131. doi:10.1186/s12939-023-01948-w 37434187 PMC10334506

[58] Kwegyir-Afful E, Verbeek J, Aziato L, Seffah JD, Räsänen K. A Liftless intervention to prevent preterm birth and low birthweight among pregnant ghanaian women: protocol of a stepped-wedge cluster randomized controlled trial. JMIR Res Protoc. 2018;7(8):e10095. doi:10.2196/10095 30139723 PMC6127499

[59] Lugina HI, Christensson K, Massawe S, Nystrom L, Lindmark G. Change in maternal concerns during the 6 weeks postpartum period: a study of primaparous mothers in Dar es Salaam, Tanzania. J Midwifery Womens Health. 2001;46(4):248-257. doi:10.1016/s1526-9523(01)00133-7 11603640

[60] Lundgren I, Berg M, Nilsson C, Olafsdottir OA. Health professionals’ perceptions of a midwifery model of woman-centred care implemented on a hospital labour ward. Women Birth. 2020;33(1):60-69. doi:10.1016/j.wombi.2019.01.004 30686654

[61] Maga G, Arrigoni C, Brigante L, et al. Developmental strategy and validation of the Midwifery Interventions Classification (MIC): a Delphi Study Protocol and results from the Developmental Phase. Healthcare (Basel). 2023;11(6):919. doi:10.3390/healthcare11060919 36981576 PMC10048446

[62] Mannocci A, Ciavardini S, Mattioli F, et al. HAPPY MAMA Project (Part 2)-Maternal Distress and Self-Efficacy: a pilot randomized controlled field trial. Int J Environ Res Public Health. 2022;19(3):1461. doi:10.3390/ijerph19031461 35162482 PMC8835492

[63] Maslin A. Nursing care counts - the hard evidence. J Adv Nurs. 2004;46(2):117. doi:10.1111/j.1365-2648.2003.02971.x 15056322

[64] McGiveron A, Foster S, Pearce J, Taylor MA, McMullen S, Langley-Evans SC. Limiting antenatal weight gain improves maternal health outcomes in severely obese pregnant women: findings of a pragmatic evaluation of a midwife-led intervention. J Hum Nutr Diet. 2015;28(suppl 1):29-37. doi:10.1111/jhn.12240 24809211

[65] McNeill J, Lynn F, Alderdice F. Public health interventions in midwifery: a systematic review of systematic reviews. BMC Public Health. 2012;12:955. doi:10.1186/1471-2458-12-955 23134701 PMC3544621

[66] Meedya S, Fahy K, Kable A. Factors that positively influence breastfeeding duration to 6 months: a literature review. Women Birth. 2010;23(4):135-145. doi:10.1016/j.wombi.2010.02.002 20299299

[67] Meedya S, Fahy K, Yoxall J, Parratt J. Increasing breastfeeding rates to six months among nulliparous women: a quasi-experimental study. Midwifery. 2014;30(3):e137-e144. doi:10.1016/j.midw.2013.12.010 24485838

[68] Morlans-Lanau M, González-Vives ML, Rodríguez-Quiroga A, Casbas MM, Klugarová J, Klugar M. Establishing midwife-led continuity of care interventions in perinatal mental health in high-risk pregnancies: a best practice implementation project. JBI Evid Implement. 2022;20(S1):S49-S58. doi:10.1097/XEB.0000000000000324 36372793

[69] Morrell CJ, Sutcliffe P, Booth A, et al. A systematic review, evidence synthesis and meta-analysis of quantitative and qualitative studies evaluating the clinical effectiveness, the cost-effectiveness, safety and acceptability of interventions to prevent postnatal depression. Health Technol Assess. 2016;20(37):1-414. doi:10.3310/hta20370 PMC4885009 27184772

[70] Nkowane AM, Ferguson SL. The World Health Organization launches the 2016-2020 Global Strategic Directions for strengthening nursing and midwifery. Nurs Econ. 2016;34(4):206-207. 29975030

[71] Ogrodniczuk JS, Piper WE. Preventing postnatal depression: a review of research findings. Harv Rev Psychiatry. 2003;11(6):291-307. doi:10.1080/10673220390264249. 14713566

[72] Panda S, Begley C. ‘Not in established labour’: Outcomes for women cared for in an Irish antenatal ward. Br J Midwifery. 2014;22(4):264-268. doi:10.12968/bjom.2014.22.4.264

[73] Pérez-Martínez E, Sebastián-Viana T, Velasco-Vázquez D, Del Gallego-Lastra R. Postpartum complications in women attended by midwives instead of obstetricians. Midwifery. 2019;75:80-88. doi:10.1016/j.midw.2019.04.009. 31051412

[74] Petersen A, Ayerle GM, Frömke C, Hecker H, Gross MM; ProGeb Study Team. The timing of interventions during labour: descriptive results of a longitudinal study. Midwifery. 2011;27(6):e267-e273. doi:10.1016/j.midw.2010.10.017 21146906

[75] Polańska K, Hanke W, Sobala W, Lowe JB. Efficacy and effectiveness of the smoking cessation program for pregnant women. Int J Occup Med Environ Health. 2004;17(3):369-377. 15683158

[76] Ray AM, Salihu HM. The impact of maternal mortality interventions using traditional birth attendants and village midwives. J Obstet Gynaecol. 2004;24(1):5-11. doi:10.1080/01443610310001620206 14675972

[77] Rodríguez-Gallego I, Vila-Candel R, Corrales-Gutierrez I, Gomez-Baya D, Leon-Larios F. Evaluation of the impact of a midwife-led breastfeeding group intervention on prevention of postpartum depression: a multicentre randomised clinical trial. Nutrients. 2024;16(2):227. doi:10.3390/nu16020227 38257120 PMC10821517

[78] Sigurðardóttir VL, Gamble J, Guðmundsdóttir B, Sveinsdóttir H, Gottfreðsdóttir H. Reviewing birth experience following a high-risk pregnancy: a feasibility study. Midwifery. 2023;116:103508. doi:10.1016/j.midw.2022.103508 36223663

[79] Simpson M, Catling C. Understanding psychological traumatic birth experiences: a literature review. Women Birth. 2016;29(3):203-207. doi:10.1016/j.wombi.2015.10.009 26563636

[80] Smoke J, Grace MC. Effectiveness of prenatal care and education for pregnant adolescents: nurse-midwifery intervention and team approach. J Nurse Midwifery. 1988;33(4):178-184. doi:10.1016/0091-2182(88)90189-9 3404281

[81] Souto SPAD, de Albuquerque RS, Silva RCGD, Guerra MJ, Prata AP. Midwifery interventions to reduce fear of childbirth in pregnant women: a scoping review protocol. JBI Evid Synth. 2020;18(9):2045-2057. doi:10.11124/JBISRIR-D-19-00198 32813435

[82] Souto SPAD, Silva RCGD, Prata AP, Guerra MJ, Couto C, Albuquerque RS. Midwives’ interventions for reducing fear of childbirth in pregnant women: a scoping review. JBI Evid Synth. 2022;20(12):2867-2935. doi:10.11124/JBIES-21-00382 35976033

[83] Spindler H, Dyer J, Bagchi K, et al. Tracking and debriefing birth data at scale: a mobile phone application to improve obstetric and neonatal care in Bihar, India. Nurs Open. 2018;5(3):267-274. doi:10.1002/nop2.134 30062019 PMC6056450

[84] Swann L, Davies S. The role of the midwife in improving normal birth rates in obese women. Br J Midwifery. 2012:20(1):7-12. doi:10.12968/bjom.2012.20.1.7

[85] Taylor Miller PG, Sinclair M, Gillen P, et al. Early psychological interventions for prevention and treatment of post-traumatic stress disorder (PTSD) and post-traumatic stress symptoms in post-partum women: a systematic review and meta-analysis. PLoS One. 2021;16(11):e0258170. doi:10.1371/journal.pone.0258170 34818326 PMC8612536

[86] Toohill J, Callander E, Gamble J, Creedy DK, Fenwick J. A cost effectiveness analysis of midwife psycho-education for fearful pregnant women - a health system perspective for the antenatal period. BMC Pregnancy Childbirth. 2017;17(1):217. doi:10.1186/s12884-017-1404-7 28693447 PMC5504805

[87] Toohill J, Callander E, Fox H, et al. Socioeconomic differences in access to care in Australia for women fearful of birth. Aust Health Rev. 2019;43(6):639-643. doi:10.1071/AH17271 30248280

[88] Truva T, Valasoulis G, Pouliakis A, et al. The effect of a structured individualized educational intervention on breastfeeding rates in Greek women. Int J Environ Res Public Health. 2021;18(21):11359. doi:10.3390/ijerph182111359 34769876 PMC8582789

[89] Türkmen H, Oran NT. Massage and heat application on labor pain and comfort: a quasi-randomized controlled experimental study. Explore (NY). 2021;17(5):438-445. doi:10.1016/j.explore.2020.08.002 32828687

[90] Türkmen H, Çetinkaya S, Kiliç H, Tuna SD, Şirvanci M, Mutlu H. The effect of ice massage applied to the SP6 point on labor pain, labor comfort, labor duration, and anxiety: a randomized clinical trial. J Midwifery Womens Health. 2024;69(4):491-498. doi:10.1111/jmwh.13600 38223923

[91] Turkstra E, Mihala G, Scuffham PA, et al. An economic evaluation alongside a randomised controlled trial on psycho-education counselling intervention offered by midwives to address women’s fear of childbirth in Australia. Sex Reprod Healthc. 2017;11:1-6. doi:10.1016/j.srhc.2016.08.003 28159118

[92] Wallace LM, Dunn OM, Alder EM, Inch S, Hills RK, Law SM. A randomised-controlled trial in England of a postnatal midwifery intervention on breast-feeding duration. Midwifery. 2006;22(3):262-273. doi:10.1016/j.midw.2005.06.004 16380197

[93] Wang TH, Pai LW, Tzeng YL, Yeh TP, Teng YK. Effectiveness of nurses and midwives-led psychological interventions on reducing depression symptoms in the perinatal period: a systematic review and meta-analysis. Nurs Open. 2021;8(5):2117-2130. doi:10.1002/nop2.764 33452740 PMC8363390

[94] Wang X, Zhu C, Liu H, Sun L, Zhu W, Gu C. The effects of a midwife-led weight management program for pregnant women: A randomized controlled trial. Int J Nurs Stud. 2023;137:104387. Retraction In: Int J Nurs Stud. 2023;148:104558. doi:10.1016/j.ijnurstu.2022.104387 36435003

[95] Warren L, Rance J, Hunter B. Eat Well Keep Active: qualitative findings from a feasibility and acceptability study of a brief midwife led intervention to facilitate healthful dietary and physical activity behaviours in pregnant women. Midwifery. 2017;49:117-123. doi:10.1016/j.midw.2016.12.002 27964858

[96] Wei D, Qian X, Hong Y, Ye R, He D. Effect of midwife intervention coupled with acupressure on the vaginal delivery rate and negative emotion in parturients with scarred uterus re-pregnancy. Am J Transl Res. 2021;13(8):9429-9436. 34540062 PMC8430064

[97] Wilkinson EL, O’Mahen HA, Fearon P, et al. Adapting and testing a brief intervention to reduce maternal anxiety during pregnancy (ACORN): study protocol for a randomised controlled trial. Trials. 2016;17:156. doi:10.1186/s13063-016-1274-8 27006007 PMC4804571

[98] Craig P, Dieppe P, Macintyre S, et al. Developing and evaluating complex interventions: the new Medical Research Council guidance. BMJ. 2008;337:a1655. doi:10.1136/bmj.a1655 18824488 PMC2769032

[99] O’Cathain A, Croot L, Duncan E, et al. Guidance on how to develop complex interventions to improve health and healthcare. BMJ Open. 2019;9(8):e029954. doi:10.1136/bmjopen-2019-029954 PMC6701588 31420394

[100] MacKenzie Bryers H, van Teijlingen E. Risk, theory, social and medical models: a critical analysis of the concept of risk in maternity care. Midwifery. 2010;26(5):488-496. doi:10.1016/j.midw.2010.07.003 20719418

[101] Alipour Z, Kheirabadi GR, Kazemi A, Fooladi M. The most important risk factors affecting mental health during pregnancy: a systematic review. East Mediterr Health J. 2018;24(6):549-559. doi:10.26719/2018.24.6.549 30079950

[102] Lindström B, Berg M, Meier Magistretti C, Perez-Botella M, Downe S. The Salutogenic approach to maternity care: from theory to practice and research. In: Church S, Firth L, Balaam MC, et al, eds. New Thinking on Improving maternity care. Pinter & Martin Ltd; 2017:chap 2.

[103] Antonovsky A. The salutogenic model as a theory to guide health promotion. Health Promot Int. 1996;11(1):11-18. doi:10.1093/heapro/11.1.11

[104] Viken B, Balaam MC, Lyberg A. A salutogenic perspective on maternity care for migrant women. In: Church S, Firth L, Balaam MC, et al., eds. New Thinking on Improving maternity care. Pinter & Martin Ltd; 2017.

[105] Lothian JA. Healthy birth practice #4: avoid interventions unless they are medically necessary. J Perinat Educ. 2014;23(4):198-206. doi:10.1891/1058-1243.23.4.198 25411540 PMC4235054

[106] de Jonge A, Dahlen H, Downe S. ‘Watchful attendance’ during labour and birth. Sex Reprod Healthc. 2021;28:100617. doi:10.1016/j.srhc.2021.100617 33774268

[107] Nieuwenhuijze MJ, Gottfreðsdóttir H. Midwifery interventions, a concept analysis to gain further understanding of the concept. Eur J Midwifery. 2023;7(suppl 1). doi:10.18332/ejm/172139

