# Supplementary material for: A concept analysis of the term midwifery intervention: A scoping review
Source: Eur J Midwifery. 2026 Mar 31;10:10.18332/ejm/216185. doi: 10.18332/ejm/216185 (PMC13178702; doi:10.18332/ejm/216185)
Supplement: Supplementary file 1 [file EJM-10-11-s1.pdf]

Supplement 1: Search result per database

Database	Search terms	Hits	Double	Excluded based on tiab	Excluded based on full article	Not obtained	Included
PubMed	"midwifery intervention" OR "midwife intervention"	22		0	5		17
Embase	"midwifery intervention" OR "midwife intervention"	22	22	0	0		0
Psychinfo	midwifery intervention OR midwife intervention	106	12	32	55		7
Psycharticles	midwifery intervention OR midwife intervention	18	0	17	1		0
Socindex	midwifery intervention OR midwife intervention	17	1	4	11		1
Cinahl	midwifery intervention OR midwife intervention	496	120	279	55	1	41
PubMed	proximity search with max. 3 words between midwifery and intervention	239	141	87	0		11
Reference lists		45			39		6
Total included		965	296	419	166	1	83

Supplement 2: Characteristics of selected publications on Midwifery Intervention

# in ref. list	Full citation, country	Type of paper	Aim	Context and setting	Population	Data collection
18	Abhari ZH, Karimi FZ, Taghizdeh Z, Mazloum SR, Asghari Nekah SM. Effects of counseling based on Gamble's approach on psychological birth trauma in primiparous women: a randomized clinical trial. J Matern Fetal Neonatal Med. 2022;35(4):668-676. Iran	Research paper, RCT	To investigate the effects of counseling based on Gamble's approach on psychological birth trauma in primiparous women.	Perinatal Health centers in Mashhad, Iran.	60 primiparous women age of 18–35 years.	Data were collected in 2018. Demographic and obstetrics characteristics; psychological birth trauma questionnaire.
19	*Abou Malham S, Hatem M, Leduc N. A case study evaluation of an intervention aiming to strengthen the midwifery professional role in Morocco: anticipated barriers to reaching outcomes. J Multidiscip Healthc. 2015;8:419-32. Morocco	Research paper, case study evaluation	To identify barriers that could potentially hinder an action plan to strengthen the midwifery professional role from achieving desired outcomes.	Professionals Two regions (Rabat and Tétouan) in Morocco participated in the implementation process.	Stakeholders pertaining to three positions and disciplines (medical administrative officers, academic directors, midwifery educators and students, nurses, midwives, obstetricians, physicians, medical directors, senior nurse and midwife managers).	Data were collected during June and July 2010 through training sessions, field observations, documents, focus groups (n=20), and semi-structured interviews (n=11) with stakeholders.
20	Adams SH, Gregorich SE, Rising SS,	Research paper, nonrandomized	To determine if women	Antenatal	101 women aged 18 years and older.	Data were collected in 2010-2011.

# in ref. list	Full citation, country	Type of paper	Aim	Context and setting	Population	Data collection
	Hutchison M, Chung LH. Integrating a nurse-midwife-led oral health intervention into centering pregnancy prenatal care: results of a pilot study. J Midwifery Womens Health. 2017;62(4):463-469. USA	controlled pilot study	receiving a brief, low-cost, and sustainable educational intervention entitled Centering-Pregnancy Oral Health Promotion had clinically improved oral health compared to women receiving standard Centering-Pregnancy care.	Women attending Centering-Pregnancy, a group prenatal care model, at 4 health centers in the San Francisco Bay Area, USA.		Dental examinations and questionnaires were administered prior to and approximately 9 weeks post intervention. Primary outcomes included the Plaque Index, percent bleeding on probing, and percent of gingival pocket depths 4 mm or greater. Secondary outcomes were self-reported oral health knowledge, attitudes (importance and self-efficacy), and behaviors (tooth brushing and flossing). Regression models tested whether pre to post changes in outcomes differed between the intervention versus the control arms.
21	Ajuebor O, McCarthy C, Li Y, Al-Blooshi SM, Makhanya N, Cometto G. Are the global	Research paper, a cross-sectional mixed methods study	To assess the progress of the Global Strategic	Professionals All six WHO regions were represented in	Most respondents (n = 30; 86%) held the position of Chief Nursing or Chief	Data were collected in 2018. A 15-question, self-reported online

# in ref. list	Full citation, country	Type of paper	Aim	Context and setting	Population	Data collection
	strategic directions for strengthening nursing and midwifery 2016-2020 being implemented in countries? Findings from a cross-sectional analysis. Hum Resour Health. 2019;17(1):54. Global		Directions for Strengthening Nursing and Midwifery 2016–2020 (SDNM) implementation at country level to ensuring that countries stay on track towards achieving universal health coverage (UHC) and the sustainable development goals (SDGs).	the respondents with from the European (n = 11; 31%) and African (n = 10; 29%) regions, one respondent was from the Southeast Asian region.	Midwifery Officer; five respondents (14%) reported holding other positions, such as ministerial adviser (n = 1), coordinating (n = 1) or educational positions (n = 3).	questionnaire was developed, including respondents' demographics (country, position and gender), use of the SDNM and use of nursing and midwifery policy frameworks. Indication of the implementation status on the 22 interventions in the SDNM using a three-level scale ("not started", "in progress" and "completed"). Open-ended questions asked about areas of need/support in implementing the SDNM.
22	Alderdice F, McNeill J, Lynn F. A systematic review of systematic reviews of interventions to improve maternal mental health and well-being. Midwifery. 2013;29(4):389-99.	Research paper, systematic review of systematic reviews	To identify non-invasive interventions in the perinatal period that could enable midwives to offer effective support to	Perinatal Interventions that could be used or coordinated by a midwife. Exclude were reviews related specifically to	Pregnant and postnatal women in economically developed countries (OECD).	Data were collected from 1999 up to October 2010. 9 databases: MEDLINE, PubMed, EBSCO (CINAHL/ British Nursing Index), MIDIRS Online Database, Web of Science, The

# in ref. list	Full citation, country	Type of paper	Aim	Context and setting	Population	Data collection
	Economically developed countries (OECD)		women within the area of maternal mental health and well-being.	obstetric interventions.		Cochrane library, CRD (NHSEED/DARE/HTA), Joanna Briggs Institute and EconLit.<br>Data were extracted using a data collection form.
23	Allen J, Kildea S, Stapleton H. How optimal caseload midwifery can modify predictors for preterm birth in young women: Integrated findings from a mixed methods study. Midwifery. 2016;41:30-38. Australia	Research paper, mixed method study	To identify possible mechanisms by which caseload midwifery reduces preterm birth for young childbearing women.	A tertiary maternity hospital in Australia providing care to approximately 500 pregnant young women (aged 21 years or less) each year. Three distinct models of care were offered: caseload midwifery, young women's clinic, and standard 'fragmented' care.	1971 young women and babies.	Data collected during 2008–2012.

# in ref. list	Full citation, country	Type of paper	Aim	Context and setting	Population	Data collection
24	Allen J, Kildea S, Hartz DL, Tracy M, Tracy S. The motivation and capacity to go 'above and beyond': Qualitative analysis of free-text survey responses in the M@NGO randomised controlled trial of caseload midwifery. Midwifery. 2017;50:148-156. Australia	Research paper, RCT	To explore whether women allocated to caseload care characterize their midwife differently to those allocated to standard care.	Perinatal The study was conducted at two metropolitan teaching hospitals in two Australian cities.	Women of all obstetric risk were eligible to participate. Inclusion criteria were: 18 years or older, less than 24 week's gestation with a singleton pregnancy.	Women were included between December 2008 – May 2011. Participants' responses to open-ended questions were collected through a 6-week postnatal survey and analysed thematically.
25	Altiner M, Secginli S, Mathiason MA, Monsen KA. Method development for describing content of multitasked interventions using the Omaha system. Res Theory Nurs Pract. 2019;33(2):147-168. Turkey	Research paper, qualitative study with observations and interviews	To develop and test a method for describing intervention content of nurse/midwife multitasked interventions using the Omaha System and Time Capture Tool (TimeCaT).	Professionals Family Health Centre in Turkey.	8 nurses/midwives.	Data were collected in 2016 through observation with TimeCaT (Time capture tool).
26	Asadzadeh L, Jafari E, Kharaghani R, Taremian F. Effectiveness of midwife-led brief	Research paper, RCT	To investigate the effectiveness of brief midwife-led counseling	Perinatal Pregnant women attending three governmental	From among 270 pregnant women screened to participate in the study, 90 women experienced a	Data were collected between September and November 2017 through a researcher-made questionnaire

# in ref. list	Full citation, country	Type of paper	Aim	Context and setting	Population	Data collection
	counseling intervention on post-traumatic stress disorder, depression, and anxiety symptoms of women experiencing a traumatic childbirth: a randomized controlled trial. BMC Pregnancy Childbirth. 2020;20(1):142. Iran		based on Gamble and colleagues' approach in decreasing post-traumatic stress disorder, depression, and anxiety symptoms among a group of women who had experienced a traumatic childbirth.	antenatal clinics of healthcare centers of Zanjan city, Iran.	traumatic childbirth. They were randomly assigned into two groups: intervention (n = 45) and control group (n = 45).	with demographic characteristics, pregnancy history, and Edinburgh postnatal depression scale before giving birth. Both groups answered Edinburgh postnatal depression scale, Hamilton's anxiety rating scale, and PCL- 5 within 72 h, 4-6 weeks, and 3 months after giving birth.
27	*Bick D, Bishop J, Coleman T, Dean S, Edwards E, Frawley H, Gkini E, Hay-Smith J, Hemming K, Jones E, Oborn E, Pearson M, Salmon V, Webb S, MacArthur C. Antenatal preventative pelvic floor muscle exercise intervention led by midwives to reduce postnatal urinary incontinence (APPEAL): protocol for a feasibility and pilot	Research paper, RCT protocol	To provide a potential assessment of the feasibility of undertaking a future definitive trial of a midwifery-led antenatal intervention to support women to perform PFME in pregnancy and reduce UI postnatally.	Perinatal Community midwifery.	Community midwifery teams and all pregnant women who receive antenatal care from participating community midwifery teams.	Primary outcome Secondary outcome Questionnaires (14-1500 women) ICIQ-UI scale and Faecal incontinence scale. Process evaluation data - Interviews with midwives and women.

# in ref. list	Full citation, country	Type of paper	Aim	Context and setting	Population	Data collection
	cluster randomised controlled trial. Pilot Feasibility Stud. 2022;8(1):231. UK					
28	Blomgren J, Wells MB, Erlandsson K, Amongin D, Kabiri L, Lindgren H. Putting co-creation into practice: lessons learned from developing a midwife-led quality improvement intervention. Glob Health Action 2023;16(1):2275866. Sweden.	Research paper, qualitative study	The objective of the study is to describe the co-creation process and explore the needs and determinants of a midwife-led quality improvement targeting evidence-based midwifery practices.	Professionals The study took place at a public national referral hospital in Uganda.	Participants for semi-structured interviews were: one leader within the policy sector, one midwife from The National Midwives Association of Uganda, two academic professionals working in midwife education programmes and three stakeholders at the clinical level, two obstetricians and one midwife manager. Also, new mothers (n = 15) and birth companions (n = 4) and 26 midwives working at the hospital.	No period of data collection was mentioned. Consisted of four stages: 1. The essential and indispensable elements of an intervention; lessons learned from providing an online capacity building programme called Midwize, which focuses on QI in midwifery, 2. interviews, FGDs and environmental observations, 3. co-creation workshops with the same participants and researchers, 4. finalising the intervention design with four of the authors and a group of seven midwives

# in ref. list	Full citation, country	Type of paper	Aim	Context and setting	Population	Data collection
						working at the hospital.
29	Borg Cunen N, McNeill J, Murray K. A systematic review of midwife-led interventions to address post partum post-traumatic stress. Midwifery 2014;30(2):170-84. Global	Research paper, systematic review	To systematically identify interventions that midwives could introduce to address post-traumatic stress in women following childbirth.	Perinatal Interventions, which could be implemented by midwives for the prevention and/or management of PTSD. Exclusion of mothers who had adverse experiences including miscarriages, stillbirths, premature births or infant's admitted to special care, etc.	Women post-partum.	Data were collected from papers published from 2002–2012 in English, databases including: Cinahl, Cochrane Library, EMBASE, Maternity and Infant Care, MEDLINE, PsycINFO, and Web of Science. Levels of evidence framework developed by Scottish Intercollegiate Guidelines Network (SIGN) 2008 was utilized. Cochrane Collaborations tool to evaluate bias. Methodological quality was supported by Smith et al, 2011.
30	Borges, S. Cystic fibrosis and caseload Midwifery. British Journal of Midwifery 2021;29(12):712-7.	Discussion paper	To examine whether there is an argument to invest in protected	Antenatal Pregnant women with cystic fibrosis	-	-

# in ref. list	Full citation, country	Type of paper	Aim	Context and setting	Population	Data collection
	UK		caseload midwifery contacts for women with cystic fibrosis, in addition to the care they receive from the obstetric and specialist teams.			
31	Borneskog C, Engström G, Islam N, Byrskog U, Pedersen C, Stromsöe A, Erlandsson K; MSc student group. Midwife educators' perceptions of the efficacy of the Objective Structured clinical assessment of life-saving interventions - a qualitative interview study in Bangladesh. Sex Reprod Healthc. 2023;37:100861. Bangladesh	Research paper, qualitative study	To examine how midwifery educators in Bangladesh perceived using OSCA as an assessment device in midwifery education for student performance in life-saving midwifery interventions.	Professionals Purposive sampling at 38 education institutions in Bangladesh during.	47 academic midwives and clinical midwives.	Data were collected in June and July 2022. Individual interviews were conducted using a pre-developed interview guide.
32	Bryce A, Butler C, Gnich W, Sheehy C, Tappin DM. CATCH: development of a	Research paper, action research	To develop, implement and evaluate a supportive	Antenatal Single maternity hospital Unit	79 pregnant smokers, age 25 and under.	Data were collected November 2002 to February 2004. Quantitative survey

# in ref. list	Full citation, country	Type of paper	Aim	Context and setting	Population	Data collection
	home-based midwifery intervention to support young pregnant smokers to quit. Midwifery 2009;25(5):473-82. UK		midwifery intervention, Community Action on Tobacco for Children's Health (CATCH), to help young pregnant smokers to quit.	West of Scotland.		and qualitative reports.
33	Caelli K, Downie J, Letendre A. Parents' experiences of midwife-managed care following the loss of a baby in a previous pregnancy. J Adv Nurs. 2002;39(2):127-36. Canada	Research study, qualitative study	To explore the impact of the Special Delivery Service (SDS), a midwife-managed intervention, developed as an addition to routine care to support and educate high-risk pregnant women and their partners subsequent to the death of a baby in a previous pregnancy.	Antenatal Women and their partners in Canada and Australia who had followed the SDS program.	Fourteen couples have completed the SDS program to date. In all, 13 participants were interviewed for this research – eight women and five men. Participants could choose if they were interviewed individually or as a couple and three women asked to be interviewed with their partners present to support them.	No indication of data collection period. All interviews were conducted in participants' homes. Participants were asked to describe how they became involved with the SDS, what it was like to be included in such a group, and what the experience of the program was like. The study was also informed by observations carried out during several months of participant observation of the SDS programme.

# in ref. list	Full citation, country	Type of paper	Aim	Context and setting	Population	Data collection
34	Coates D, Foureur M. The role and competence of midwives in supporting women with mental health concerns during the perinatal period: A scoping review. Health Soc Care Community 2019 ;27(4):e389-e405.<br>Global	Research paper, scoping review	To explore the role and competence of midwives in delivering mental healthcare.	Professionals<br>Studies relevant to the role of midwives in the management and treatment of perinatal mental health issues were included; studies focused on screening and referral were excluded.	Thirty papers met inclusion criteria, including studies about the knowledge, skills, and attitudes of midwives and student midwives; the effectiveness of educational interventions in improving knowledge and skills; the delivery of counselling or psychosocial interventions by midwives; and barriers and enablers to embedding midwife-led mental healthcare in practice.	The databases PubMed, Maternity and Infant Care, Science Citation Index, Social Sciences Citation Index, Medline, Science Direct and CINAHL were searched for the period from 2011 to 2018. Information relevant to the research question was extracted from each paper. Thematic analysis of the extracted information was conducted.
35	Dai Y, Min H, Sun L, Wang X, Zhu C, Gu C. Assessing women's and health professionals' views on developing a midwifery-led mobile health app intervention in pregnancy: A descriptive qualitative	Research paper, descriptive qualitative study	To explore women's and health professionals' views on the development of a midwifery-led mHealth app intervention in antenatal care and their	Antenatal<br>A tertiary maternity hospital in Shanghai.	15 pregnant and postpartum women and 10 healthcare professionals including obstetricians, midwives and obstetric nurses.	Data were collected from June to August 2022.<br>A semi-structured interview guide (Box 1) was used.<br>Interview questions for health professionals<br>1. Demographics<br>2. What aspects do you think should be

# in ref. list	Full citation, country	Type of paper	Aim	Context and setting	Population	Data collection
	study. J Adv Nurs. 2024.<br>China		demands for app functionality.			optimized in current maternal healthcare services?<br>3. How do you usually provide antenatal care to pregnant women in your workplace, and are there any challenges or problems you face?<br>4. What are your views on the use of mHealth applications to deliver maternal health services?<br>5. If a midwifery-led app for maternal health services is to be developed, What functionalities or information do you think should be included and what specific issues would you expect to address?<br>Interview questions for women<br>1. Demographics<br>2. How is your present pregnancy planned? What do you expect

# in ref. list	Full citation, country	Type of paper	Aim	Context and setting	Population	Data collection
						from maternal health care services?<br>3. From conception to the present, how do you seek information on antenatal care? (e.g., obstetrician-led antenatal clinic, midwife clinic, community health services, books, television, Internet, etc.)<br>4. What do you think of the information or health advice that you have received about pregnancy and antenatal care? What aspects do you think are inadequate or can be further improved?<br>5. What do you think of using a midwifery-led<br>mobile health app to provide antenatal care? What kind of information or support would be helpful to you, or what other

# in ref. list	Full citation, country	Type of paper	Aim	Context and setting	Population	Data collection
						aspects would you like to address?
36	Dawson A, Cohen D, Candelier C, Jones G, Sanders J, Thompson A, Arnall C, Coles E. Domiciliary midwifery support in high-risk pregnancy incorporating telephonic fetal heart rate monitoring: a health technology randomized assessment. J Telemed Telecare 1999;5(4):220-30. UK	Research paper, RCT	To compare enhanced domiciliary care with conventional care among high- risk pregnant women.	Antenatal South Wales – tertiary hospital setting and outpatient care.	80 women in South Wales valley.	No indication of data collection period. Several psychological measures were used: State-Trait Anxiety Inventory, Edinburg Postnatal Depression Scale, Satisfaction evaluated by several questionnaires, Economics Risk assessment, Patient born costs, non hospital costs, hospital costs. Reduction in use of hospital beds and lower maternal anxiety.
37	de Wolff MG, Midtgaard J, Johansen M, Rom AL, Rosthøj S, Tabor A, Hegaard HK. Effects of a Midwife-Coordinated Maternity Care Intervention (ChroPreg) vs. Standard Care in Pregnant Women with	Research paper, RCT	To evaluate the effects of a midwife-coordinated maternity care intervention (ChroPreg) in pregnant women with CMC.	Antenatal The trial was conducted at the Department of Obstetrics, Rigshospitalet, Copenhagen University Hospital.	262 pregnant women with a single fetus, one or more CMCs before pregnancy, aged ≥18 years, and who understood written and spoken Danish were eligible to participate.	Data were collected from October 2018–August 2020, with follow-up completion by October 2020. The primary outcome was the total length of hospital stay (LOS). Secondary outcomes were patient-reported

# in ref. list	Full citation, country	Type of paper	Aim	Context and setting	Population	Data collection
	Chronic Medical Conditions: Results from a Randomized Controlled Trial. Int J Environ Res Public Health 2021;18(15):7875. Denmark			The Department is a tertiary referral center with ≥5000 births annually.	130 intervention group and 128 control group.	outcomes measuring psychological well-being and satisfaction with maternity care, health utilization, and maternal and infant outcomes.
10	Edqvist M, Hildingsson I, Mollberg M, Lundgren I, Lindgren H. Midwives' management during the second stage of labor in relation to second-degree tears-an experimental study. Birth 2017;44(1):86-94. Sweden	Research paper, experimental cohort study (prospective)	To evaluate a multifaceted midwifery intervention designed to reduce second-degree tears among primiparous women.	Natal<br>Two maternity wards in Stockholm.	Nulliparous Swedish-speaking women (597), gestational age ≥37+0 weeks with spontaneous onset of labor or induction of labor.	Data were collected from November, 2013 to<br>June, 2014 in maternity ward 1, and from April,<br>2014 to February, 2015 in maternity ward 2. Intention-to treat analysis and descriptive statistics used to present the data. Crude and adjusted odds ratios with a 95% confidence interval calculated between women who received the intervention and those who received standard care.

# in ref. list	Full citation, country	Type of paper	Aim	Context and setting	Population	Data collection
38	Evans K, Spiby H, Morrell CJ. Developing a complex intervention to support pregnant women with mild to moderate anxiety: application of the Medical Research Council framework. BMC Pregnancy Childbirth 2020;20(1):777. UK	Research paper, development of an intervention	To report the stages of an intervention development utilising the MRC framework for developing complex interventions [24]	Professionals University of Nottingham.	Midwives and midwife support workers in UK.	No indication of data collection period. The development followed the MRC framework for complex interventions, utilising psychological theory, review level evidence and professional and public involvement. Two systematic reviews were completed which helped identify potentially beneficial intervention components.
39	Evans K, Moya H, Lambert M, Spiby H. Developing a training programme for midwives and maternity support workers facilitating a novel intervention to support women with anxiety in pregnancy. BMC Pregnancy Childbirth 2022;22(1):662. UK	Research paper, development of an intervention	To report the development of a training programme to prepare midwives and maternity support workers to facilitate the intervention.	Professionals University of Nottingham.	Midwives and midwife support workers in UK.	No indication of data collection period. Kern's six-step approach for curriculum development was used to identify midwives and maternity support workers training needs to help support pregnant women with anxiety and facilitate a supportive intervention. The

# in ref. list	Full citation, country	Type of paper	Aim	Context and setting	Population	Data collection
						stages of development included feedback from a preliminary study, stakeholder engagement, a review of the literature surrounding midwives' learning and support needs and identifying and supporting the essential process and functions of the RAPID intervention.
40	Evans K, Spiby H, Slade M, Jomeen J, Beckhelling J. RAPID-2 study protocol: a cluster randomised feasibility trial of a midwife facilitated intervention for pregnant women with symptoms of mild to moderate anxiety. BMJ Open 2022;12(10):e064659. UK.	Research paper, protocol of a cluster randomised feasibility study.	The RAPID intervention aims to provide suitable, timely support for women with mild-moderate anxiety during pregnancy	Antenatal Four National Health Service Trusts in England that provide maternity care will be cluster randomised to the RAPID intervention	Eligibility includes nulliparous women at 16–20 weeks of pregnancy (n=50) and two midwives from each research site.	Data will be collected to assess: (1) the number of women accessing the site and completing eligibility screening, (2) the number of eligible women and (3) the number of women who consent to participate. A qualitative evaluation will be added focusing on women's and facilitators' views on participating in the intervention

# in ref. list	Full citation, country	Type of paper	Aim	Context and setting	Population	Data collection
41	Fenwick J, Gamble J, Creedy D, Barclay L. Women´s experiences of the PRIME midwifery counselling intervention: Promoting Resilience In Mothers Emotions. Women & Birth 2011;24:S11-2. Australia	Oral presentation of a paper (part of a larger study in two phases)	To evaluate of the effectiveness of a midwifery-led counselling intervention.	Antenatal Antenatal care setting Australia.	Forty women who had been allocated to either intervention or control group.	No indication of data collection period.
42	Fenwick J, Gamble J, Creedy DK, Buist A, Turkstra E, Sneddon A, Scuffham PA, Ryding EL, Jarrett V, Toohill J. Study protocol for reducing childbirth fear: a midwife-led psycho-education intervention. BMC Pregnancy Childbirth 2013;13:190. Australia	Research paper, protocol for RCT	To test the efficacy of a psycho-education counselling intervention offered by midwives to address women's fear of childbirth.	Perinatal Antenatal clinics of three metropolitan teaching hospitals in south-east Queensland, Australia.	Pregnant women in their second trimester of pregnancy will be recruited and screened. Women reporting high childbirth fear will be randomly allocated to the intervention or control group.	Data will be collected at recruitment during the second trimester, 36 weeks of pregnancy, and 4–6 weeks after birth. The outcomes of the RCT on obstetric outcomes, maternal psychological well-being, parenting confidence, birth satisfaction, and future birth preference were analysed by intention to treat and reported here.
43	Fenwick J, Toohill J, Gamble J, Creedy DK, Buist A, Turkstra E, Sneddon A, Scuffham	Research paper, RCT	To review women's current expectations	Perinatal Antenatal clinics of three metropolitan	1410 women were screened for high childbirth fear (W-DEQ ≥66), women were	Recruitment took place between May 2012 and June 2013. A two armed non-

# in ref. list	Full citation, country	Type of paper	Aim	Context and setting	Population	Data collection
	PA, Ryding EL. Effects of a midwife psycho-education intervention to reduce childbirth fear on women's birth outcomes and postpartum psychological wellbeing. BMC Pregnancy Childbirth 2015;15:284. Australia		and feelings around fear of childbirth, support the expression of feelings, and provide a framework for women to identify and work through distressing elements of childbirth [4, 5].	teaching hospitals in south-east Queensland, Australia.	randomised (intervention n = 170; controls n = 169). 184 (54 %) returned data for final analysis at 6 weeks postpartum (intervention n = 91; controls n = 93).	blinded parallel (1:1) multi-site randomised controlled trial. The outcomes of the RCT on obstetric outcomes, maternal psychological well-being, parenting confidence, birth satisfaction, and future birth preference were analysed by intention to treat and reported here.
44	Fernandez Turienzo C, Bick D, Bollard M, Brigante L, Briley A, Coxon K, Cross P, Healey A, Mehta M, Melaugh A, Moulla J, Seed PT, Shennan AH, Singh C, Tribe RM, Sandall J. POPPIE: protocol for a randomised controlled pilot trial of continuity of midwifery care for women at increased risk of preterm birth. Trials 2019;20(1):271. UK	Research paper, protocol for RCT	To test whether a model of care combining continuity of midwife care with rapid referral to a specialist obstetric clinic throughout pregnancy, intrapartum and the postpartum period is feasible and improves experience and outcomes for	Perinatal Hospital and community settings in collaboration with specialist obstetric clinic care.	350 pregnant women at increased risk of preterm birth.	The composite primary outcome is the initiation of any of the following interventions appropriately provided for the prevention and/or management of preterm labour and birth (antibiotics for urinary tract infections, smoking cessation and domestic violence advocacy referrals, transvaginal scan assessments of the

# in ref. list	Full citation, country	Type of paper	Aim	Context and setting	Population	Data collection
			women at increased risk of preterm birth.			cervix, fetal fibronectin assessments, cerclage insertion, progesterone administration, corticosteroid administration, magnesium sulphate administration, admission for observation and in utero transfer). Demography, medical history and current pregnancy health information and psycho-social health data will be collected prior to randomisation at baseline visit (recruitment). Follow-up data will continue up to 6–8 weeks postpartum for mothers and until discharge from neonatal intensive care unit (NICU) for babies (up to 3months).

# in ref. list	Full citation, country	Type of paper	Aim	Context and setting	Population	Data collection
45	Firouzan L, Kharaghani R, Zenoozian S, Moloodi R, Jafari E. The effect of midwifery led counseling based on Gamble's approach on childbirth fear and self-efficacy in nulligravida women. BMC Pregnancy Childbirth 2020;20(1):522. Iran	Research paper, RCT	To investigate the effectiveness of a psycho-educational intervention by midwives on decreasing childbirth fear and self-efficacy among first-time pregnant women who were afraid of giving birth.	Antenatal The antenatal clinics of Zanjan city, Iran.	A number of 80 pregnant women participated in the study. They had received a score of ≥66 on the Wijma delivery expectancy/ experience questionnaire.	Data were collected from February to September 2019. All women answered the demographic information questionnaire, W-DEQ, and childbirth self-efficacy inventory at pretest and post-test.
46	Gamble J, Creedy D, Moyle W, Webster J, McAllister M, Dickson P. Effectiveness of a counselling intervention after a traumatic childbirth: a randomized controlled trial. Birth 2005;32:11-19. Australia	Research paper, RCT	To assess a midwife-led brief counseling intervention post-partum.	Postnatal Antenatal clinics of three maternity hospitals in Brisbane.	103 women who were over 18 years of age, in the last trimester of pregnancy, expected to give birth to a live infant, and able to complete questionnaires and interviews in English. Women experiencing stillbirth or neonatal death were excluded.	Participants were recruited between April 2001 and February 2002. EPDS, DASS-21 (Depression Anxiety and Stress Scale-21), MSSS (maternity social support scale). Outcome measure: posttraumatic stress symptoms, depression, self-blame, and confidence about a future pregnancy.

# in ref. list	Full citation, country	Type of paper	Aim	Context and setting	Population	Data collection
47	Gamble J, Toohill J, Slavin V, Creedy D, Fenwick J. Identifying barriers and enablers as a first step in the implementation of a midwife-led psychoeducation counseling framework for women fearful of birth. International Journal of Childbirth 2017;7(3):152-168. Australia	Research paper, mixed method study	To explore organizational factors, including barriers and possible solutions that may impact on the successful application of the midwife psychoeducation intervention in practice.	Antenatal<br>A publically funded maternity unit in South East Queensland. Continuity of midwifery care (caseload) was available to a limited number of women (approximately 10%). Other than general practitioner (GP)-shared care and obstetric high-risk care, pregnancy care was generally provided by midwives.	Survey among care providers. Seven focus groups were conducted over a 2-week period.<br>All 28 attendees were midwives. Five service leaders were interviewed and included two specialist doctors, the service director, and two midwife managers.	No indication of data collection period.<br>Descriptive statistics and frequencies were used to describe participant characteristic.<br>Qualitative data were generated from open-ended questions on the survey (n 5 55), focus groups, and interviews. Latent content analysis was used to elicit the underlying meaning of content focusing on rich description (Polit & Beck, 2012).
9	George A, Dahlen HG, Blinkhorn A, Ajwani S, Bhole S, Ellis S, Yeo A, Elcombe E, Johnson M. Evaluation of a midwifery initiated oral health-dental	Research paper, multi-centre randomized controlled trial.	To access effectiveness of midwifery-initiated oral health dental service program and if it	Antenatal<br>Three large metropolitan public hospitals in Sydney Australia.	638 women in three groups between 12 and 20 weeks of pregnancy.	Women recruited for trial November 2012–October 2015. Use of dental service<br>Women´s oral health knowledge<br>Quality of oral health

# in ref. list	Full citation, country	Type of paper	Aim	Context and setting	Population	Data collection
	service program to improve oral health and birth outcomes for pregnant women: A multi-centre randomised controlled trial. Int J Nurs Stud. 2018;82:49-57. Australia		improves the uptake of dental service, ect.			Oral health outcomes
48	Gonzalez-Plaza E, Bellart J, Arranz Á, Luján-Barroso L, Crespo Mirasol E, Seguranyes G. Effectiveness of a step counter smartband and midwife counseling intervention on gestational weight gain and physical activity in pregnant women with obesity (Pas and Pes Study): randomized controlled trial. JMIR Mhealth Uhealth 2022;10(2):e28886. Spain	Research paper, RCT	To evaluate the effectiveness of a complex digital health intervention, using a smartband and app with midwife counseling, on gestational weight gain (GWG) and physical activity (PA) in women who are pregnant and have obesity and analyze its impact on maternal and	Antenatal The maternal–fetal department of the Hospital Clinic of Barcelona.	150 women who were pregnant and had obesity.	Women recruited between June 2018 to October 2020. The validated Spanish versions of the International Physical Activity Questionnaire–Short Form and the System Usability Scale were used. Satisfaction was measured on a 1- to 5-point Likert scale.

# in ref. list	Full citation, country	Type of paper	Aim	Context and setting	Population	Data collection
			perinatal outcomes.			
49	*Gu C, Lindgren H, Wang X, Zhang Z, Liang S, Ding Y, Qian X. Developing a midwifery service task list for Chinese midwives in the task-shifting context: a Delphi study. BMJ Open 2021;11(7):e044792.<br>China	Research paper, Delphi survey	To develop and validate a midwifery-led task list in the task-shifting context.	Professionals<br>Twenty university hospitals, three non-university hospitals and four university colleges from nine provincial regions of China.	Purposive non-probability sampling of a national panel of experts in maternal healthcare, obstetrics, nursing and midwifery.	Data collected between 1 July and 30 September 2019. Experts in the panel were asked to rate each midwifery service item regarding importance, feasibility and advancement on a 5-point scale, in order to determine those best suited for midwifery-led practice in China.
50	Heins HC Jr, Nance NW, McCarthy BJ, Efird CM. A randomized trial of nurse-midwifery prenatal care to reduce low birth weight. Obstet Gynecol. 1990;75:341-5.<br>USA	Research paper, RCT	To test the hypothesis that if women at increased risk for LBW had lower rate of LBW in a nurse-midwife intervention group than in a standard care group, offered by an obstetrician.	Perinatal<br>South Carolina, US.	Women attending state-funded prenatal clinics if they had a score of 10 points or more on the first visit and women who had had a previous LBW infants (1346).	Data were collected between July 1983 and October 1987.<br>Demographics<br>Birth weight

# in ref. list	Full citation, country	Type of paper	Aim	Context and setting	Population	Data collection
51	Hodnett ED, Stremler R, Willan AR, Weston JA, Lowe NK, Simpson KR, Fraser WD, Gafni A; SELAN (Structured Early Labour Assessment and Care by Nurses) Trial Group. Effect on birth outcomes of a formalised approach to care in hospital labour assessment units: international, randomised controlled trial. BMJ. 2008;337:a1021. North America en UK	Research paper, RCT	To assess if a complex nursing and midwifery intervention (structured care) in hospital labour unit increases the likelihood of spontaneous vaginal birth and improves other maternal and neonatal outcomes.	Natal Multicenter, 20 North America and UK hospitals	4996 women	Women enrolled between 1 May 2003 and 6 March 2007. Rate of spontaneous vaginal birth. Women´s experience of helpfulness and amount of attention from staff.
52	Homer CS, Besley K, Bell J, Davis D, Adams J, Porteous A, Foureur M. Does continuity of care impact decision making in the next birth after a caesarean section (VBAC)? a randomised controlled trial. BMC Pregnancy Childbirth 2013;13:140. Australia	Research paper, RCT-protocol	To determine whether midwifery continuity of care for women with a previous CS increases the proportion of women who attempt vaginal birth in their current pregnancy.	Natal Two hospital sites in Australia.	Eligible pregnant women, whose most recent previous birth was by lower-segment CS, will be randomly allocated 1:1 to an intervention group or control group.	The majority of clinical data required for the study are routinely collected and available in hospital records. Two questionnaires at 36 weeks gestation (during pregnancy) and at 6–8 weeks post-partum (after the birth) will enable women to

# in ref. list	Full citation, country	Type of paper	Aim	Context and setting	Population	Data collection
						report on their experiences.
53	Huang CJ, Han W, Huang CQ. Effect of Internet + continuous midwifery service model on psychological mood and pregnancy outcomes for women with high-risk pregnancies. World J Psychiatry 2023;13(11):862-871. China	Research paper, retrospective analysis of data	To explore the effectiveness of a midwife-led Internet + continuous midwifery service model for women with high-risk pregnancy.	Perinatal Hospital in Shanghai	Clinical data of 439 women with HRP, among them, 239 pregnant women underwent routine obstetric management, and 200 pregnant women underwent Internet + continuous midwifery service mode management	Clinical data from April to December 2022.<br>The State-Trait Anxiety Inventory, Edinburgh Postnatal Depression Scale, and analysis of delivery outcomes to compare psychological mood and the incidence of adverse delivery outcomes between the two groups.
54	van der Hulst LA, van Teijlingen ER, Bonsel GJ, Eskes M, Bleker OP. Does a pregnant woman's intended place of birth influence her attitudes toward and occurrence of obstetric interventions? Birth 2004;31(1):28-33.<br>NL	Research paper, survey study	To examine the impact of women's intended place of birth (home or hospital) and the course of pregnancy and labor when attended by midwives.	Women were enrolled in 25 independently working midwifery practices in the Netherlands.	625 low-risk pregnant women, gestation 20 to 24 weeks.	In 1998–1999, midwives in these 25 practices were asked to approach 25 consecutive pregnant women of 20 to 24 weeks' gestation, who were fluent in Dutch.<br>A questionnaire was specifically designed for this study.
55	Jimenez-Barragan M, Del Pino Gutierrez A, Garcia JC, Monistrol-	Research paper, protocol for RCT	The intervention aims to improve mental state of	Antenatal Pregnant women ≥18	70 women will be recruited and	Data will be collected using the Two Whooley questions,

# in ref. list	Full citation, country	Type of paper	Aim	Context and setting	Population	Data collection
	Ruano O, Coll-Navarro E, Porta-Roda O, Falguera-Puig G. Study protocol for improving mental health during pregnancy: a randomized controlled low-intensity m-health intervention by midwives at primary care centers. BMC Nurs. 2023;22(1):309. Spain		pregnant women during pregnancy, work through breathing, mindfulness and muscle relaxation techniques and compare with women attending standard care.	years followed in the primary care centers of the Sexual and Reproductive Health Care of Mútua Terrassa, Barcelona, Spain.	screened during their pregnancy.	two Generalized Anxiety Disorder-2 [GAD-2] questions, self-administered Edinburg Postnatal Depression scale.
56	Khademioore S, Ebrahimi E, Khosravi A, Movahedi S. The effect of an mHealth application based on continuous support and education on fear of childbirth, self-efficacy, and birth mode in primiparous women: A randomized controlled trial. PLoS One 2023;18(11):e0293815. Iran	Research paper, RCT	To evaluate the effectiveness of an interactive mobile health application named Tele-midwifery with an emphasis on continuous care and education, on FOC, self-efficacy, and childbirth mode in primiparous women.	Perinatal The prenatal clinic of Baharlou Hospital in Tehran, Iran.	70 primiparous women having FOC (confirmed by the score of 38 and above on the Wijma delivery expectancy/experience questionnaire (W-DEQ)), being between 18 to 40 years of age, pregnant with a singleton fetus, 26–29 weeks of pregnancy.	Women were recruited from February to April 2020. The Wijma delivery expectancy/experience questionnaire and the Childbirth Self-Efficacy Inventory were used to measure the FOC and self-efficacy at baseline, eight weeks after the intervention and after birth.
57	Khan Z, Vowles Z, Fernandez Turienzo C, Barry Z, Brigante L,	Research paper, systematic review	To identify and evaluate the current evidence	Countries included Australia,	46 index studies with any methodological design.	Papers up to 8/11/2022.

# in ref. list	Full citation, country	Type of paper	Aim	Context and setting	Population	Data collection
	Downe S, Easter A, Harding S, McFadden A, Montgomery E, Page L, Rayment-Jones H, Renfrew M, Silverio SA, Spiby H, Villarroel-Williams N, Sandall J. Targeted health and social care interventions for women and infants who are disproportionately impacted by health inequalities in high-income countries: a systematic review. Int J Equity Health 2023;22(1):131. Global		related to targeted health and social care service interventions in high-income countries.	Canada, Chile, Hong Kong, UK and USA.		Twelve databases were searched, inclusion criteria included interventions that targeted disadvantaged populations which provided a component of clinical care that differed from standard maternity care.
58	Kwegyir-Afful E, Verbeek J, Aziato L, Seffah JD, Räsänen K. A Liftless Intervention to Prevent Preterm Birth and Low Birthweight Among Pregnant Ghanaian Women: Protocol of a Stepped-Wedge Cluster Randomized	Research paper, protocol for a unidirectional cross-over study	The proposed liftless intervention aims to decrease lifting exposure during pregnancy among Ghanaian women.	Antenatal The trial will be conducted at 10 public antenatal clinics in Ghana.	The clinics will recruit 1000 participants within a period of 60 weeks.	The primary outcome is pregancy length. Secondary outcomes will be; Birthweight, compliance with the intervention, Prevalence of lower back pain or pelvic pain, Premature uterine contractions, uterine contractions.

# in ref. list	Full citation, country	Type of paper	Aim	Context and setting	Population	Data collection
	Controlled Trial. JMIR Res Protoc. 2018;7(8):e10095. Ghana					
59	Lugina HI, Christensson K, Massawe S, Nystrom L, Lindmark G. Change in maternal concerns during the 6 weeks postpartum period: a study of primaparous mothers in Dar es Salaam, Tanzania. J Midwifery Womens Health 2001;46(4):248-57. Tanzania	Research paper, cohort study	To describe postpartum concerns of primiparas.	Postnatal Tanzania – district hospital.	79 with uncomplicated pregnancies - mothers in Dar es Salaam.	Data collection from September 1995 to January 1997. Questionnaire used for structured interview. Two follow up visits.
60	Lundgren I, Berg M, Nilsson C, Olafsdottir OA. Health professionals' perceptions of a midwifery model of woman-centred care implemented on a hospital labour ward. Women Birth 2020;33(1):60-69. Sweden/Iceland	Research paper, qualitative design with focus group interviews	To explore whether, when adopted by midwives on labour wards, a midwifery model of woman-centred care (MiMo) was useful in practice from the viewpoint of a variety of	Professionals A hospital-based labour ward in Sweden	43 participants: midwives (n = 16), obstetricians (n = 8), assistant nurses (n = 11) and managers (n = 8).	Nine focus group interviews met between January and March 2015. The text from interviews was analysed using content analysis.

# in ref. list	Full citation, country	Type of paper	Aim	Context and setting	Population	Data collection
			health professionals.			
61	Maga G, Arrigoni C, Brigante L, Cappadona R, Caruso R, Daniele MAS, Del Bo E, Ogliari C, Magon A. Developmental strategy and validation of the midwifery interventions classification (mic): a Delphi study protocol and results from the developmental phase. Healthcare (Basel) 2023;11(6):919. Global/Italy	Research paper, literature review and Delphi study protocol	To define a Midwifery Interventions Classification, an evidence-based, standardized taxonomy and classification of midwifery interventions.	Participants from Italy for Delphi.	Healthcare professionals (midwives), healthcare researchers (with expertise in Delphi methodology), and service users (who experienced a physiological pregnancy and birth within the last five years) will be eligible panelists for the Delphi surveys.	No date of search mentioned. Three primary databases: PubMed, CINAHL, and Scopus. Search using the exact keywords once, such as maternal and newborn health, midwifery, and quality indicators. Inclusion criteria: (a) papers in the English or Italian language with full-text availability without time limits; (b) all types of study design; (c) studies describing midwifery interventions during pregnancy, childbirth, and the postnatal period in any care setting; and (d) studies conceived within a physiological framework of maternity care.

# in ref. list	Full citation, country	Type of paper	Aim	Context and setting	Population	Data collection
62	Mannocci A, Ciavardini S, Mattioli F, Massimi A, D'Egidio V, Lia L, Scaglietta F, Giannini A, Antico R, Dorelli B, Svelato A, Orfeo L, Benedetti Panici P, Ragusa A, La Torre G, Group HM. HAPPY MAMA project (part 2)-maternal distress and self-efficacy: a pilot randomized controlled field trial. Int J Environ Res Public Health 2022;19(3):1461. Italy	Research paper, Pilot RCT	To assess if a midwifery intervention is able to increase the maternal self-efficacy and reduce the stress level during the first six months after birth.	Postnatal Two different hospitals in Rome.	Total 51 mothers.	The recruitment period was October–December 2019. An online questionnaire was used to obtain socio-demographic data including age, civil status (single or not), employment (student/worker/no worker), educational level (middle school/high school/university), ethnicity, the birth date, primiparous (yes/no), the number of children living at home, and age, vaginal birth (yes/no), and characteristics of breastfeeding practice.
63	Maslin A. Nursing care counts - the hard evidence. J Adv Nurs. 2004;46(2):117. Global	Editorial	The aim is to highlight the importance and dilemma of definition and effectiveness of nursing and	Not relevant	Not relevant	Not relevant

# in ref. list	Full citation, country	Type of paper	Aim	Context and setting	Population	Data collection
			midwifery interventions.			
64	McGiveron A, Foster S, Pearce J, Taylor MA, McMullen S, Langley-Evans SC. Limiting antenatal weight gain improves maternal health outcomes in severely obese pregnant women: findings of a pragmatic evaluation of a midwife-led intervention. J Hum Nutr Diet 2015;28 Suppl 1:29-37.<br>UK	Research paper, evaluation of intervention and non-intervention group	To determine whether one-to-one antenatal guidance from midwives and healthy lifestyle advisors resulted in a lower gestational weight gain and a prevalence of the common complications of pregnancy and labour that are associated with severe obesity.	Perinatal pregnant women attending first dating antenatal ultrasound clinics at Lincoln Hospital (UK).	89 pregnant women with a body mass index (BMI) >35 kg m^2.	Data were collected between April 2012 and February 2013. Weight in pregnancy, weight gain over pregnancy. Odds ratios for pregnancy and labour complications with adjustment for potential confounding factors (maternal age, parity, gravidae, socioeconomic status, marital status and ethnicity).
65	McNeill J, Lynn F, Alderdice F. Public health interventions in midwifery: a systematic review of systematic reviews. BMC Public Health 2012;12:955.<br>Economically developed countries	Research paper, systematic review of systematic reviews	To identify evidence of effective public health interventions from good quality systematic reviews that could be	Professionals<br>Various contexts and settings.	36 reviews.	MEDLINE, Pubmed, EBSCO, CRD, MIDIRS, Web of Science, The Cochrane Library and Econlit were searched in October 2010. Papers divided in three parts and within each part several head topics; antenatal (screening,

# in ref. list	Full citation, country	Type of paper	Aim	Context and setting	Population	Data collection
			conducted by midwives.			supplementation, support, education, mental health).
66	Meedya S, Fahy K, Kable A. Factors that positively influence breastfeeding duration to 6 months: a literature review. Women Birth 2010;23(4):135-45. Global	Research paper, review of literature	To explore what modifiable factors positively influence breastfeeding duration to 6 months post-partum.	Postnatal US, Canada, Singapore, India, England, Scotland.	Ten papers. Women breastfeeding, numbers identified in each paper.	MEDLINE, CINAL, Maternity and Infant Care, Cochrane Database of systematic reviews were searched for papers from years 2000—2009.
67	Meedya S, Fahy K, Yoxall J, Parratt J. Increasing breastfeeding rates to six months among nulliparous women: a quasi-experimental study. Midwifery. 2014;30(3):e137-44. Australia	Research paper, quasi-experimental study	To evaluate the effectiveness of a multiphased midwifery intervention called the 'Milky Way' on any breastfeeding rates until six months.	Postnatal Tertiary, metropolitan hospital in Sydney.	420 women, ended as 366.	Recruitment from 2009 up to 2011.Data collection based on antenatal baseline information, postnatal data collected via phone interviews x3.
68	Morlans-Lanau M, González-Vives ML, Rodríguez-Quiroga A, Casbas MM, Klugarová J, Klugar M. Establishing midwife-led continuity of care interventions in perinatal mental	Research paper, pre–post following the JBI Evidence Implementation framework was used.	To improve the quality of continuity of care and emotional well-being in women with high-risk pregnancies.	Antenatal Obstetric Service of the Infanta Leonor Hospital, belonging to the public network of	120 registered pregnant women whose antenatal care of high-risk pregnancy as well as delivery were in the hospital.	Baseline data in November 2019 using the software JBI PACES with the aim to assess compliance with the standards of best practice. Audit criteria were selected from two JBI Evidence

# in ref. list	Full citation, country	Type of paper	Aim	Context and setting	Population	Data collection
	health in high-risk pregnancies: a best practice implementation project. JBI Evid Implement 2022;20(S1):S49-S58. Spain			hospitals in Madrid, Spain.		Summaries: Antenatal Psychosocial Assessment and Postpartum Depression.
69	*Morrell CJ, Sutcliffe P, Booth A, Stevens J, Scope A, Stevenson M, Harvey R, Bessey A, Cantrell A, Dennis CL, Ren S, Ragonesi M, Barkham M, Churchill D, Henshaw C, Newstead J, Slade P, Spiby H, Stewart-Brown S. A systematic review, evidence synthesis and meta-analysis of quantitative and qualitative studies evaluating the clinical effectiveness, the cost-effectiveness, safety and acceptability of interventions to prevent postnatal depression. Health	Research paper, systematic review	To (1) evaluate the clinical effectiveness, cost-effectiveness, acceptability and safety of antenatal and postnatal interventions for pregnant and postnatal women to prevent PND; (2) apply rigorous methods of systematic reviewing of quantitative and qualitative studies, evidence synthesis and	Perinatal<br>All universal, selective and indicated preventive interventions for pregnant women and women in the first 6 postnatal weeks were included.	From 3072 records identified, 122 papers (86 trials) were included in the quantitative review. From 2152 records, 56 papers (44 studies) were included in the qualitative review.	We searched MEDLINE, EMBASE, Science Citation Index and other databases from inception to July 2013.<br>All outcomes were included, focusing on the Edinburgh Postnatal Depression Scale (EPDS), diagnostic instruments and infant outcomes.

# in ref. list	Full citation, country	Type of paper	Aim	Context and setting	Population	Data collection
	Technol Assess. 2016;20(37):1-414.<br>Global		decision-analytic modelling to evaluate the preventive impact on women, their infants and their families; and (3) estimate cost-effectiveness.			
70	*Nkowane AM, Ferguson SL. The World Health Organization Launches the 2016-2020 Global Strategic Directions for Strengthening Nursing and Midwifery. Nurs Econ. 2016;34(4):206-7.<br>Global	Policy paper	To strengthening nursing and midwifery and the health workforce in general.	The global strategic directions provide the framework for strengthening nursing and midwifery services to help countries achieve universal health coverage and the Sustainable Development Goals.	Midwives and nurses around the world.	-
71	Ogrodniczuk JS, Piper WE. Preventing postnatal depression: a review of research findings. Harv Rev	Research paper, systematic review of RCTs	To offer an overview on selective and indicated measures	Postnatal Primary prevention interventions that span the	The 8 included studies (1) were published in English language journals, (2) were focused on adults, (3)	Searches of MEDLINE and PsychINFO were performed to identify English-language papers of randomized,

# in ref. list	Full citation, country	Type of paper	Aim	Context and setting	Population	Data collection
	Psychiatry 2003;11(6):291-307. Global		directed at preventing postnatal depression.	entire biopsychosocial spectrum. In addition, it articulates the nature of the high-risk groups of women toward whom each indicated intervention is directed.	used standardized measures of depression, (4) reported on primary prevention interventions rather than treatments for postnatal depression, and (5) used a randomized, controlled design.	controlled trials published from 1990 to 2003 focusing on primary prevention of postnatal depression in both the general and high-risk populations.
72	Panda S, Begley C. 'Not in established labour': Outcomes for women cared for in an Irish antenatal ward. British Journal of Midwifery 2014 ;2(4):264-8. Ireland	Research paper, retrospective descriptive audit of charts	To ascertain the outcomes of labour and describe the interventions performed by midwives for women admitted to the antenatal ward at term gestation with labour-related symptoms.	Intrapartum Coombe Women and Infants University Hospital, Dublin, Ireland.	47 women in early labour.	A retrospective review of hospital records from women admitted to the hospital in November 2012. Data collected included characteristics, number and reasons of vaginal examinations performed in the antenatal ward, pain relief, reason for transfer to delivery suite, reason for augmentation of labour, mode of birth, and duration of labour.
73	Pérez-Martínez E, Sebastián-Viana T,	Research paper, a quasi-	To evaluate changes in the	Postnatal	Low risk postpartum women attended at	Data collected from clinical records

# in ref. list	Full citation, country	Type of paper	Aim	Context and setting	Population	Data collection
	Velasco-Vázquez D, Del Gallego-Lastra R. Postpartum complications in women attended by midwives instead of obstetricians. Midwifery 2019;75:80-88.<br>Spain	experimental retrospective study	frequency of visits to the hospital emergency department due to puerperal complications in low risk postpartum women attended by midwives instead of obstetricians.	University hospital in Madrid, Spain.	the maternity unit of the Fuenlabrada University Hospital. The intervention group comprised 1308 women, whereas there were 1313 women in the control group.	between March 2013 and February 2015. Visit to the emergency department of the Fuenlabrada Hospital due to complications in the 40 days after postpartum clinical discharge. Other variables included in the study were: age (years), sex of the newborn, area and health zone of the mothers' residence, promotion of maternal-newborn bonding, performance of antenatal monitoring, pregnancy course, destination of the newborn after the birth, early initiation of breastfeeding, primiparity, breastfeeding, use of urinary catheter, immediate postpartum, perineal tear, episiotomy, suture, evolution

# in ref. list	Full citation, country	Type of paper	Aim	Context and setting	Population	Data collection
						during the puerperium and early discharge.
74	Petersen A, Ayerle GM, Frömke C, Hecker H, Gross MM; ProGeb Study Team. The timing of interventions during labour: descriptive results of a longitudinal study. Midwifery 2011;27(6):e267-73. Germany	Research paper, longitudinal prospective and retrospective cohort study	To describe the timing and frequency of interventions during labour, and in addition to compare the timings of the interventions against the partogram action lines.	Natal<br>47 hospitals in Lower Saxony, Germany.	3963 births of nulliparae and multiparae with singletons in vertex presentation giving birth between April and October 2005. The participation rate for the prospectively recruited sample (n=1169) was 4.7%.	Data were collected between April and October 2005.<br>Time intervals until intrapartal interventions.<br>Outcome variables were duration of labour and mode of birth.
75	Polańska K, Hanke W, Sobala W, Lowe JB. Efficacy and effectiveness of the smoking cessation program for pregnant women. Int J Occup Med Environ Health. 2004;17(3):369-77. Poland	Research paper, RCT	To evaluate the effectiveness of anti-smoking counseling in the population of pregnant women from the maternity centers in Łódź, central Poland.	Prenatal<br>Public maternity centers in Łódź, Poland.	149 current smokers and 56 spontaneous quitters were randomized into the smoking cessation intervention and 144 current smokers and 37 spontaneous quitters were included in the control group.	A randomized trial was conducted between December 1, 2000, and December 31, 2001.<br>All subjects filled in a questionnaire at the beginning, after that midwives collected data on smoking habits using interviews on factors: education of women, number of cigarettes smoked at enrollment, smoking in previous pregnancies, smoking husband.

# in ref. list	Full citation, country	Type of paper	Aim	Context and setting	Population	Data collection
76	Ray AM, Salihu HM. The impact of maternal mortality interventions using traditional birth attendants and village midwives. J Obstet Gynaecol. 2004;24(1):5-11. Africa, Asia and Latin America	Research paper, systematic review	To examine maternal mortality interventions that focus on improved management of labour and delivery through training of midwives and traditional birth attendants in order to understand better the program components contributing to intervention successes and shortcomings.	Natal<br>Studies addressing maternal mortality in developing countries. The interventions focused on improved management of labour and delivery.	15 papers on 15 maternal mortality interventions. The interventions employed traditional birth attendants and/or village midwives at a community level; that is, outside health facilities.	A literature search was performed in the MEDLINE and PUBMED databases for 1966 through February 2003 using the following keywords: maternal mortality, developing countries, midwifery, traditional birth attendants and maternal death. Relevant papers, including review papers and commentaries, were collected for cross-referencing and further database searches were conducted with author names in order to access additional resources not readily retrieved in the keyword search.
77	Rodríguez-Gallego I, Vila-Candel R, Corrales-Gutierrez I, Gomez-Baya D, Leon-Larios F. Evaluation of	Reaasearch paper, a multicentre randomised clinical trial	To assess the effectiveness of a midwife-led breastfeeding support group	Postnatal<br>A multicentric cluster randomised	A total of 382 women participated.	Data were collected from October 2021 to May 2023. Sociodemographic and obstetric clinical data

# in ref. list	Full citation, country	Type of paper	Aim	Context and setting	Population	Data collection
	the Impact of a Midwife-Led Breastfeeding Group Intervention on Prevention of Postpartum Depression: A Multicentre Randomised Clinical Trial. Nutrients. 2024;16(2):227. Spain.		intervention on breastfeeding rates, postpartum depression and general self-efficacy.	controlled trial with control and intervention groups, not blinded, conducted in Andalusia (southern Spain)		were collected by a questionnaire designed for this purpose. The General Self-efficacy Scale (GSE) and the Edinburgh Postnatal Depression Scale (EPDS).<br>The type of breastfeeding was recorded at hospital discharge, as well as at three established follow-up time points.
78	Sigurðardóttir VL, Gamble J, Guðmundsdóttir B, Sveinsdóttir H, Gottfreðsdóttir H. Reviewing birth experience following a high-risk pregnancy: A feasibility study. Midwifery 2023;116:103508. Iceland	Research paper, a mixed-method study	To review their birth experiences, but limited knowledge exists about appropriate interventions and the feasibility of providing this care for women following high-risk pregnancies.	Natal<br>A high-risk antenatal outpatient clinic at Landspítali University Hospital in Reykjavík, Iceland.	30 women who experienced high-risk pregnancies were invited to write about and review their birth experience with a known midwife 4–6 weeks postpartum.	Data were collected from November 2018 to September 2019. Data including birth outcomes, birth experience and experience of the intervention were collected by questionnaires from women at two time points before and after the counselling intervention. Midwives providing the intervention

# in ref. list	Full citation, country	Type of paper	Aim	Context and setting	Population	Data collection
						completed diaries and participated in focus group interview to explore their experiences of the process.
79	Simpson M, Catling C. Understanding psychological traumatic birth experiences: A literature review. Women Birth 2016;29(3):203-7. Global	Research paper, systematic literature review	To gain a better understanding of factors contributing to birth trauma, and the efficacy of interventions that exist in the literature.	Limited to systematic reviews or original research of either high to moderate scientific quality.	A total of 21 papers were included in this literature review,	Databases were searched in April 2015 and included EBSCO Academic Search Complete, Science Direct, CINAHL, Maternity and Infant Care database, Wiley Science, Medline and the Cochrane Library. Search terms included: birth trauma; traumatic childbirth; childbirth; psychological aspects; and childbirth post-traumatic stress.
80	Smoke J, Grace MC. Effectiveness of prenatal care and education for pregnant adolescents: nurse-midwifery intervention and team approach. J	Research paper, controlled study	To evaluate the change in pregnancy-related knowledge by pre- and post-education testing and to	Antenatal<br>The experimental group consisted of patients receiving prenatal care from the	116 pregnant adolescents, meeting the following inclusion criteria:<br>1. Less than l8-years-old (no more than 17 years 11 months) at first prenatal visit.	All patients delivered in 1985.<br>Self-selection was a factor by the patient's choice of clinic to attend.<br>A pretest was given at first or second visit to

# in ref. list	Full citation, country	Type of paper	Aim	Context and setting	Population	Data collection
	Nurse Midwifery 1988;33(4):178-84. USA		compare the pregnancy outcomes in two adolescent groups.	Adolescent Obstetrical Services (AOS) of the University Hospital of Cincinnati. The professional staff of AOS was coordinated by a certified nurse-midwife. The control group received prenatal care from the Cincinnati Health Department Clinics (CHDC).	2. Gestation of 36 weeks or less at the first visit.<br>3. Expecting first full-term pregnancy.<br>4. Eligible for nurse-midwifery management.	either clinic. If the patient had three or more additional visits to either clinic she was then given a post test, which was a repeat of the pretest, to assess the increase in knowledge. The three visits allowed for the occurrence of a minimal teaching time. The majority (95%) of the posttests were given during postpartum hospitalization. The remainder were completed before two weeks postpartum.
81	Souto SPAD, de Albuquerque RS, Silva RCGD, Guerra MJ, Prata AP. Midwifery interventions to reduce fear of childbirth in pregnant women: a scoping review protocol. JBI	Research paper, scoping review protocol	To map and analyze midwife interventions to reduce fear of childbirth in pregnant women.	Antenatal<br>This scoping review will consider studies that integrated interventions led and implemented by midwives	This scoping review will consider studies that include interventions to reduce fear of childbirth in pregnant women, led and implemented by midwives, during the antenatal period, in all	The databases searched will include MEDLINE (PubMed), CINAHL Complete (EBSCO), APA PsycINFO (EBSCO), Scopus, Embase, Web of Science, SciELO, Cochrane Database of Systematic Reviews,

# in ref. list	Full citation, country	Type of paper	Aim	Context and setting	Population	Data collection
	Evid Synth. 2020;18(9):2045-2057. Global			during the antenatal period.	possible birth scenarios. Quantitative, qualitative, and mixed methods studies will be included.	Cochrane Methodology Register, Cochrane Central Register of Controlled Trials, MedicLatina (EBSCO), Academic Search Complete (EBSCO), ERIC (EBSCO), and Psychology and Behavioral Sciences Collection (EBSCO). Sources of unpublished and gray literature were Repositório Científico de Acesso Aberto de Portugal (RCAAP), ProQuest Dissertations and Theses (ProQuest), British Library EThOS, OvidSP Resource Center, Banco de Teses da CAPES, and OpenGrey.
82	Souto SPAD, de Albuquerque RS, Silva RCGD, Guerra MJ, Prata AP. Erratum to: Midwifery interventions to	Erratum	The term ‘‘midwifery intervention(s)’’ should read ‘‘midwife intervention(s).’’			

# in ref. list	Full citation, country	Type of paper	Aim	Context and setting	Population	Data collection
	reduce fear of childbirth in pregnant women: a scoping review protocol. JBI Evid Synth. 2021;19(11):3206. Erratum for: JBI Evid Synth. 2020;18(9):2045-2057. Global		The term ''midwife intervention'' indicates that the intervention is conducted specifically by a health professional with the title of ''midwife,'' which is the population and concept of interest.			
83	Souto SPAD, Silva RCGD, Prata AP, Guerra MJ, Couto C, Albuquerque RS. Midwives' interventions for reducing fear of childbirth in pregnant women: a scoping review. JBI Evid Synth. 2022;20(12):2867-2935.<br>Global	Research paper, scoping review	To identify midwives' interventions for reducing fear of childbirth in pregnant women and to examine their characteristics.	Antenatal<br>All potential midwifery practice settings were considered for inclusion: hospitals, obstetric units/ maternity units inside hospitals, units led by midwives, health centers, and women's homes. All	34 papers were included. This scoping review considered all studies that included a midwife or team of midwives who worked independently or within a multidisciplinary team.	Papers published between January 1981 (year of publication of the first paper on FOC) and October 2020 were included.<br>The databases searched were MEDLINE (PubMed), CINAHL Complete (EBSCO), APA PsycINFO (EBSCO), Scopus, Embase, Web of Science, SciELO, Cochrane Database of Systematic Reviews,

# in ref. list	Full citation, country	Type of paper	Aim	Context and setting	Population	Data collection
				geographical and cultural contexts were included.		Cochrane Methodology Register, Cochrane Central Register of Controlled Trials, MedicLatina (EBSCO), Academic Search Complete (EBSCO), ERIC (EBSCO), and Psychology and Behavioral Sciences Collection (EBSCO). Sources of unpublished and gray literature were Repositório Científico de Acesso Aberto de Portugal (RCAAP), ProQuest Dissertations and Theses (ProQuest), British Library EThOS, OvidSP Resource Center, Banco de Teses da CAPES, and OpenGrey.
84	Spindler H, Dyer J, Bagchi K, Ranjan V, Christmas A, Cohen SR, Sterling M, Shah MB, Das A, Mahapatra T, Walker D. Tracking and debriefing birth data at	Research paper, survey using questions in mobile app.	To assess changes over time in skill and knowledge related to the use of evidence-based practices	Births in primary health clinics (PHCs) in Bihar, India.	Observational data from 5,799 births was recorded by 120 NMMs in 320 PHCs.	Data was collected from January 2015 up to January 2017. Prompted questions in the App after every live

# in ref. list	Full citation, country	Type of paper	Aim	Context and setting	Population	Data collection
	scale: A mobile phone application to improve obstetric and neonatal care in Bihar, India. Nurs Open 2018;5(3):267-274. India		associated with quality of maternal and neonatal care during a nurse midwife mentoring intervention at primary health clinics.			birth witnessed. The App consisted of questions around three main themes, "What went well?", "What needed improvement?" and "What can be done differently next time?".
85	Swann L, Davies S. The role of the midwife in improving normal birth rates in obese women. British Journal of Midwifery 2012:20(1):7-12. UK	Discussion paper	-	Antenatal / natal All birthing environments	Obese women	Not applicable
86	Taylor Miller PG, Sinclair M, Gillen P, McCullough JEM, Miller PW, Farrell DP, Slater PF, Shapiro E, Klaus P. Early psychological interventions for prevention and treatment of post-traumatic stress disorder (PTSD) and post-traumatic stress	Research paper, a systematic review and meta-analysis	To investigate the effectiveness of early psychological interventions in reducing or preventing post-traumatic stress symptoms and post-traumatic stress disorder in post-partum	Midwifery or clinician led early psychological interventions administered within 72 hours following traumatic childbirth.	11 studies from Europe, North America, Australia and Iran were identified that evaluated the effectiveness of a range of early psychological interventions.	Up to 1st May 2020 searches of electronic databases were performed; Embase, PsychInfo, AMED (Allied and Complementary Medicine), MEDLINE, through the OVID database and the Global Health Library. Searches of CINAHL, Published

# in ref. list	Full citation, country	Type of paper	Aim	Context and setting	Population	Data collection
	symptoms in post-partum women: A systematic review and meta-analysis. PLoS One 2021;16(11):e0258170. Global		women within twelve weeks of a traumatic birth.			International Literature on Traumatic Stress (PILOTS) and Dissertations and Thesis through Proquest, The Lancet and The Cochrane Library were also performed. The World Health Organisation International Clinical Trials Registry Platform and ClinicalTrials.gov were included in the search and hand searched bibliographies of the included studies and for citations of papers were performed.
87	Toohill J, Callander E, Gamble J, Creedy DK, Fenwick J. A cost effectiveness analysis of midwife psycho-education for fearful pregnant women - a health system perspective for the antenatal period. BMC	Research paper, cost effectiveness analysis	To determine the cost-effectiveness of a midwife-led psycho-education intervention for women fearful of birth.	Prenatal Women in their second trimester of pregnancy at antenatal booking clinics across three maternity hospitals in	1410 pregnant women in southeast Queensland, Australia were screened for childbirth fear (W-DEQ A ≥ 66). Women with high scores (n = 339) were randomised to the BELIEF Study (Birth Emotions and Looking	No indication of data collection period. Demographic, obstetric information, birth preference and psychosocial measures were collected at recruitment and 36 weeks; with birth method and health

# in ref. list	Full citation, country	Type of paper	Aim	Context and setting	Population	Data collection
	Pregnancy Childbirth 2017;17(1):217. Australia			Queensland, Australia	to Improve Expectant Fear) to receive psycho-education (n = 170) at 24 and 34 weeks of pregnancy or to the control group (n = 169).	service use returned by participants six weeks following birth. The time frame for this economic evaluation was the duration of pregnancy, with analysis conducted from a health system perspective.
88	Toohill J, Callander E, Fox H, Lindsay D, Gamble J, Creedy D, Fenwick J. Socioeconomic differences in access to care in Australia for women fearful of birth. Aust Health Rev. 2019;43(6):639-643. Australia	Research paper, secondary analysis was conducted of data obtained during a randomised controlled	To determine whether healthcare use and access to continuity models are equal across different indicators of socioeconomic status for women who are fearful of birth.	Prenatal Women in their second trimester of pregnancy at antenatal booking clinics across three maternity hospitals in Queensland, Australia.	1410 pregnant women in southeast Queensland, Australia were screened for childbirth fear (W-DEQ A ≥ 66). Women with high scores (n = 339) were randomised to the BELIEF Study (Birth Emotions and Looking to Improve Expectant Fear) to receive psycho-education (n = 170) at 24 and 34 weeks of pregnancy or to the control group (n = 169).	No indication of data collection period. Demographic, obstetric information, birth preference and psychosocial measures were collected at recruitment and 36 weeks; with birth method and health service use returned by participants six weeks following birth. The time frame for this economic evaluation was the duration of pregnancy, with analysis conducted from a health system perspective.

# in ref. list	Full citation, country	Type of paper	Aim	Context and setting	Population	Data collection
89	Truva T, Valasoulis G, Pouliakis A, Gkorezi-Ntavela I, Pappa D, Bargiota A, Garas A, Grivea I, Daponte A. The effect of a structured individualized educational intervention on breastfeeding rates in Greek women. Int J Environ Res Public Health 2021;18(21):11359. Greece	Research paper, cohort study	To investigate the effect of a structured individualized lactation educational intervention by a midwife on increasing breastfeeding rates in women with endocrine disorders and low-risk women compared to women receiving standard care, 24 months after delivery.	Postpartum The University Hospital of Larisa.	A total population of 200 individuals (100 low-risk pregnant women and 100 pregnant women with GDM and/or hypothyroidism in pregnancy) were included to the final study.	Data collection was from March 2017 to March 2020 and included a prenatal interview, a postnatal interview and telephone interviews with questionnaires collecting quantitative data.
90	Türkmen H, Oran NT. Massage and heat application on labor pain and comfort: A quasi-randomized controlled experimental study. Explore 2021;17(5):438-445. Turkey	Research paper, quasi-randomized controlled experimental study	To determine the effects of sacral massage and heat application on the perceptions of labor pain and comfort level in pregnant women.	Intrapartum The delivery room of Public Hospital in Turkey.	98 primiparous pregnant women with vaginal delivery expectancy, term pregnancy, single healthy fetus, cephalic presentation, active labor stage (4-5cm dilation), and Oxytocin infusion.	A 3-part survey was used for data collection between June and December 2016. The questionnaires included a personal information form (PIF), a numerical rating scale (NRS) for measuring pain, and

# in ref. list	Full citation, country	Type of paper	Aim	Context and setting	Population	Data collection
						the Childbirth Comfort Questionnaire (CCQ) for evaluating comfort.
91	Türkmen H, Çetinkaya S, Kiliç H, Tuna SD, Şirvanci M, Mutlu H. The effect of ice massage applied to the sp6 point on labor pain, labor comfort, labor duration, and anxiety: a randomized clinical trial. J Midwifery Womens Health 2024. Turkey	Research paper, single-masked, randomized controlled trial	To determine the effects of ice massage applied to the SP6 acupressure point during labor on labor pain, labor comfort, labor duration, and anxiety.	The delivery room of Atatürk City Hospital in Turkey.	100 nulliparous women with a singleton pregnancy, a healthy fetus, 38 to 40 weeks' gestation, expecting a vaginal birth, cephalic presentation, being in stage 1 of childbirth, and having 4 to 5 cm of cervical dilation.	Data collection was between December 2021 and July 2022. Baseline demographic and health characteristics were collected using a Personal Information Form(PIF). The primary outcomes included severity of labor pain (VAS), state anxiety during labor (STAI), and duration of the active phase of labor (4-10 cm cervical dilatation). The secondary outcome was comfort level during labor (Childbirth Comfort questionnaire (CCQ)).
92	Turkstra E, Mihala G, Scuffham PA, Creedy DK, Gamble J, Toohill J, Fenwick J. An economic evaluation alongside a	Research paper, RCT	To test an antenatal psycho-education intervention by midwives in	The intervention was provided in addition to usual care offered by	We recruited 1410 women in their second trimester attending antenatal clinics of three hospitals in South-East	Participants were recruited from May 2012 to June 2013. The primary outcome measure was reduction in childbirth

# in ref. list	Full citation, country	Type of paper	Aim	Context and setting	Population	Data collection
	randomised controlled trial on psycho-education counselling intervention offered by midwives to address women's fear of childbirth in Australia. Sex Reprod Healthc. 2017;11:1-6. Australia		reducing women's childbirth fear.	publicly funded maternity services in Australia.	Queensland, Australia, who were able to communicate sufficiently in English, and aged 16 years or older. Three hundred and thirty-nine women (339/ 1410, 24%) reporting high childbirth fear (defined as a score of ≥66 on the WDEQ-A) were allocated to the intervention (n = 170) or control (n = 169) groups.	fear according to the Wijma Delivery Expectancy/Experience Questionnaire version A (WDEQ-A).
93	Wallace LM, Dunn OM, Alder EM, Inch S, Hills RK, Law SM. A randomised-controlled trial in England of a postnatal midwifery intervention on breast-feeding duration. Midwifery 2006;22(3):262-73. UK	Research paper, RCT	To determine whether postnatal 'hands off' care by midwives on positioning and attachment of the newborn baby improves breast-feeding duration.	Postpartum, from first feed. In eight postnatal wards of four maternity hospitals in the English Midlands.	370 primiparous mothers with term babies who intended to breast feed, and could sit out of bed to do so.	Women were recruited between May 2001 and May 2002. Allocation was initially by telephone randomisation; later randomisations used computers installed in each ward. Data on breast feeding were collected using diaries. The care given by the trial midwife was recorded by her in a bespoke intervention

# in ref. list	Full citation, country	Type of paper	Aim	Context and setting	Population	Data collection
						checklist. Semi-structured interviews were conducted in the mothers' home at 6 weeks.
94	Wang TH, Pai LW, Tzeng YL, Yeh TP, Teng YK. Effectiveness of nurses and midwives-led psychological interventions on reducing depression symptoms in the perinatal period: A systematic review and meta-analysis. Nurs Open 2021;8(5):2117-2130.<br>Global	Research paper, a systematic review and meta-analysis	To evaluate the effectiveness of nurses and midwives-led psychological interventions on the perinatal depressive symptoms.	Pre and postnatal<br>The trials were conducted mainly in the UK, Australia, Norway, Iceland, Nigeria, Spain and France. There are four papers on primary care for psychological intervention by midwives.	4,141 women participated (2,636 in the intervention group; 1,505 in the control group). The average age of the women in the studies was between 26.2–32.2 years.	All eligible studies were published between 1989–2019. We searched published literature in English as the main from PubMed, MEDLINE, Cochrane Library, EMBASE, Web of Science and CINAHL.
95	Wang X, Zhu C, Liu H, Sun L, Zhu W, Gu C. The effects of a midwife-led weight management program for pregnant women: A randomized controlled trial. Int J Nurs Stud. 2023;137:104387.	Research paper, RCT	To examine the effects of a midwife-led weight management program on improving appropriate gestational	Prenatal<br>Tertiary women's hospital in Eastern China.	426 pregnant women booking for antenatal care. The inclusion criteria were:<br>(1) women aged over 18 years old with singleton pregnancy, and 11-13+6 weeks gestation at	Women were enrolled from June to September 2020.<br>Data were collected at 3 time points, including recruitment (T1), 35–36 weeks gestation (T2), and 2–3 days postpartum (T3).

# in ref. list	Full citation, country	Type of paper	Aim	Context and setting	Population	Data collection
	Retraction in: Int J Nurs Stud. 2023;148:104558. China		weight gain, health literacy, experience of antenatal care, and maternal and neonatal outcomes among Chinese pregnant women.		recruitment; (2) able to speak, read and write in Chinese; (3) low risk at recruitment in absence of mental, medical or obstetrical complications.	T1: Pregnant women were required to complete their basic information (age, education background, occupation) and health literacy assessment. T2:Women were invited to complete the Quality of Prenatal Care Questionnaire and the Health Literary Questionnaire during 35 – 36 weeks gestation. T3: Maternal and infant outcomes were collected through electronic medical records by the researcher.
96	Warren L, Rance J, Hunter B. Eat Well Keep Active: Qualitative findings from a feasibility and acceptability study of a brief midwife led intervention to facilitate healthful dietary and physical	Research paper, qualitative interviews 6 weeks after the delivery of the intervention during pregnancy	To assess the feasibility and acceptability of the ‘Eat Well Keep Active’ intervention program designed to promote healthy eating and	Prenatal Midwife led care.	Pregnant women suitable for Midwife Led Care and therefore deemed to be ‘low risk’ from a large maternity unit in South Wales (n=20).	No indication of data collection period. Semi-structured interviews were used to evaluate the acceptability and participant perception of efficacy of the intervention between six and eight weeks

# in ref. list	Full citation, country	Type of paper	Aim	Context and setting	Population	Data collection
	activity behaviours in pregnant women. Midwifery 2017;49:117-123.<br>UK		physical activity in pregnant women. This brief midwife led intervention was based upon the Self Determination Theory (SDT) framework and utilised motivational interviewing and individualised goal setting.			after delivery of the initial session.<br>Qualitative analysis.
97	Wei D, Qian X, Hong Y, Ye R, He D. Effect of midwife intervention coupled with acupressure on the vaginal delivery rate and negative emotion in parturients with scarred uterus re-pregnancy. Am J Transl Res. 2021;13(8):9429-9436.<br>China	Research paper, experimental study	To investigate the effect of midwife intervention coupled with acupressure on the vaginal delivery rate and negative emotion in parturients with scarred uterus re-pregnancy.	Intrapartum Parturients with scarred uterus re-pregnancy who were admitted to The People's Hospital of Chizhou in China.	85 parturients with scarred uterus re-pregnancy who agreed to trial-produce vaginally after assessment. Parturients were divided into the research group (43 parturients) who received midwife intervention coupled with acupressure and the control group (42 parturients) who	Patients were recruited from April 2018 to December 2019.<br>Allocation to control or research group is not described. Data collection is not described.<br><br>The delivery mode, birth process time, postpartum hemorrhage, maternal and infant outcomes, negative emotion

# in ref. list	Full citation, country	Type of paper	Aim	Context and setting	Population	Data collection
					received routine nursing intervention.	improvement, intrapartum pain score and family care satisfaction of parturients between the two groups were compared.
98	Wilkinson EL, O'Mahen HA, Fearon P, Halligan S, King DX, Greenfield G, Dunkley-Bent J, Ericksen J, Milgrom J, Ramchandani PG. Adapting and testing a brief intervention to reduce maternal anxiety during pregnancy (ACORN): study protocol for a randomised controlled trial. Trials 2016;17:156. UK	Research paper, study protocol for RCT	To develop a brief intervention for antenatal anxiety, with a focus on embedding the delivery of the treatment within routine antenatal care.	Prenatal Participants will be recruited through UK National Health Service (NHS) antenatal scanning clinics at their 12-week scan in London and the South West of England. The intervention will be delivered to participants in these locations as a part of antenatal services.	Pregnant women who have not had any children, entering their second trimester, aged 18 and over. They score in the top quartile of scores on the Generalised Anxiety Disorder 7-item scale (GAD-7) [38] at screening.	Several time points of data collection. Diversity of outcome measures.

* Found through references in included publications

Supplement 3: Data extraction table I for the concept analysis of midwifery intervention

* Separate interventions are underlined, if a similar intervention is used in various publications the oldest mention is underlined.

# in ref. list	First author, year, country	Exact naming of the intervention*	Definition	Attributes	Antecedents	Consequences	Boundaries
18	Abhari, 2020, Iran	Midwifery-led counseling intervention	A counselling intervention to prevent and mitigate psychological birth trauma.<br><br>Based on an intervention <u>developed by Gamble et al</u>. can be adopted by obstetricians and midwives.	Midwives were involved in developing and providing the intervention. Establishing a therapeutic relationship between the midwife and the parturient woman. Acceptance of the maternal perceptions toward labour, supporting mothers in expressing their emotions. Eliminating the ambiguities for the mother. Establishing links between behaviours, emotions, and labour, reviewing the phases and	Trained midwife.	Psychological birth trauma.	It is not a psychotherapeutic counselling approach, but rather a preventive technique.

# in ref. list	First author, year, country	Exact naming of the intervention*	Definition	Attributes	Antecedents	Consequences	Boundaries
				modes of labour and delivery. Fostering social support. Facilitating maternal adaptation. Promoting positive maternal perceptions, and finding solutions.			
19	Abou Malham, 2015, Morocco	Midwifery intervention	An intervention (action plan) aiming to strengthen the midwifery professional role.	Midwives in pole position that offer skilled care.	Preconditions relate to the legal framework, social representations, and media support in the sociocultural system and the practice environment, networks and communication mechanisms, and characteristics related to the role and the readiness in the professional system.	Quality of maternity care, maternal, infant, and neonatal health outcomes.	-
20	Adams, 2017, USA	Nurse-midwife-led oral health intervention	An intervention embedded in Centering-Pregnancy to promote oral health.	Nurse-midwives participated in the development. Nurse-midwives performed the intervention.	Midwives trained for Centering-Pregnancy and for the intervention.	Oral hygiene (Plax index, bleeding, pocket depths).	-

# in ref. list	First author, year, country	Exact naming of the intervention*	Definition	Attributes	Antecedents	Consequences	Boundaries
				Intervention was embedded in Centering-Pregnancy.			
21	Ajuebor, 2019, global	Nursing and midwifery policy intervention Nursing and midwifery intervention	Intervention/ program to address the specific obstacles to optimized contributions of nurses and midwives to UHC and PHC goals.	The strength of nursing and midwifery.	Optimizing policy development, effective leadership, management and governance.	Realization of universal health coverage (UHC) and the Sustainable Development Goals (SDGs).	-
22	Alderdice, 2013, economically developed countries (OECD)	A midwifery-led intervention Midwifery interventions	Non-invasive interventions in the perinatal period that enable midwives to offer effective care to women within the area of maternal mental health and well-being.	Can be provided by a midwife. Non-invasive. Fits in models of midwifery-led care.	Not clear.	Enable midwives to offer appropriate support and co-ordination of care within the area of maternal mental health and well-being.	Non-midwife interventions and invasive interventions.
23	Allen, 2016, Australia	Complex intervention of caseload midwifery	The intervention provided caseload midwifery.	An intervention performed by a primary midwife. 24-hour telephone contact with her midwife or back-up, a booking visit in her home,	-	Preterm birth. Start of pregnancy care. Disclosure of risks, engaging in self-care activities and accepting referrals for assistance.	Standard fragmented care.

# in ref. list	First author, year, country	Exact naming of the intervention*	Definition	Attributes	Antecedents	Consequences	Boundaries
				community-based antenatal care that occurred in a group with other young pregnant women, a familiar midwife in labour (one of the four midwives in the group), and postnatal home visiting for 4–6 weeks after birth.			
24	Allen, 2017, Australia	Intervention of caseload midwifery	No clear definition. Caseload midwifery provides high-level relational continuity whereby childbearing women receive antenatal, intrapartum and postnatal care from a primary midwife and her/his back-up midwives.	Care given by a midwife who offers an Empowering and Endorphic approach. Time for conversations. Continuity of care, covering the whole perinatal period. Midwives were also involved in the development and seem the initiator of the intervention.	Availability of this type of care.	Women's experience of childbirth.	Other models of care
25	Altiner, 2019, Turkey	Multitasking midwifery/	Any task done by midwives in the	Any two or more overlapping tasks	Nurses and midwifes family health centres	Overview of interventions taking	No clear boundary between nursing and

# in ref. list	First author, year, country	Exact naming of the intervention*	Definition	Attributes	Antecedents	Consequences	Boundaries
		nurse intervention	center are regarded as midwifery intervention.	done by a midwife/nurse at one time.	(sometimes only nurses in text)	place. Emotional responses associated with workload, stress, and fatigue more strain.	midwifery interventions.
26	Asadzadeh, 2020, Iran	A midwife-led brief counseling intervention	Intervention for postpartum women experiencing PTSD symptoms. The intervention approach was based on Gamble and colleagues' protocol.	Midwives performed the intervention and were involved in the development. Midwives conduct counselling session with women. The intervention emphasized on the therapeutic relationship between the midwife and the person, accepting and working with perceptions, filling in the missing pieces, finding connections between the event and their emotions and behaviors, reviewing labor	Trained midwife	Post-traumatic stress disorder, depression, and anxiety symptoms.	-

# in ref. list	First author, year, country	Exact naming of the intervention*	Definition	Attributes	Antecedents	Consequences	Boundaries
				management, increasing social support, reinforcing positive approaches to coping, and exploring solutions.			
27	Bick, 2022, UK	A midwife-led antenatal intervention	An intervention to support women to perform pelvic floor muscle exercises in pregnancy.	Midwives were conducting the intervention.	Structured training program of midwives facilitated by midwives.	Feasibility of midwives to do the intervention as part of routine antenatal care. Postnatal urinary incontinence.	-
28	Blomgren, 2023, Sweden	A midwife-led quality improvement intervention	The intervention targets to bridge the evidence-to-practice gap within midwifery and effectively apply this across different settings.	The intervention is developed and delivered by midwives (Midwize Ambassadors), including peer-assisted learning, using existing structures, educational material, and building stakeholder relationships.	The intervention would start with more guidance from the support team consisting of researchers with midwife-led quality improvement competence and a clinical midwifery teacher and gradually transition to being entirely led by the Midwize Ambassador group. A three-day onsite train-the-trainers programme led by the support team.	Improved healthcare quality for women and newborns.	Restricted to midwives.

# in ref. list	First author, year, country	Exact naming of the intervention*	Definition	Attributes	Antecedents	Consequences	Boundaries
29	Borg Cunen, 2014, global	Midwife-led interventions	Interventions that could be implemented by a midwife to support women with post traumatic stress after childbirth.	Performed by midwives	(Specialized) training for midwives	PTSD.	Intervention not implemented by midwives.
30	Borges, 2021, UK	Caseload midwifery as a legitimate intervention	An intervention by midwives that improves health outcomes in pregnant women with cystic fibrosis.	Performed by midwives and involved in the development. Continuity of care.	Midwives working in a small team.	Health. Feasibility to invest in caseload midwifery.	Care without continuity.
31	Borneskog, 2023, Bangladesh	Life-saving midwifery interventions	No definition	Performed by midwives	(Specialized) training for midwives	Maternal and neonatal outcomes.	
32	Bryce, 2009, UK	A home-based midwifery intervention<br>A supportive midwifery intervention	A responsive smoking cessation service that would meet the particular needs of pregnant women aged 25 years and under	Performed by a specialist midwife, also involved in the development. Holistic approach seeking to meet wider health and social care needs. Social support for the pregnant smoker and her partner.	Trained midwife. Young pregnant women attending a hospital clinic.	Smoking cessation. Feasibility to implement home-based care to young pregnant smoker (women).	A group of women multiple complex needs, often from inequalities associated with poverty, gender and social exclusion only a project midwife which was a challenge during periods of absence, etc.

# in ref. list	First author, year, country	Exact naming of the intervention*	Definition	Attributes	Antecedents	Consequences	Boundaries
33	Caelli, 2002, Canada	Midwife-managed intervention	Intervention additional to routine care to support and educate high-risk pregnant women and their partners subsequent to the death of a baby in a previous pregnancy.	Initiated, developed and managed by midwives. Offered in collaboration with the client's obstetrician or general practitioner and with their preferred hospital.	Not clear.	Couples' experiences. Being informed and supported.	No additional care.
34	Coates, 2019, high-, middle- and low-income countries	Midwife-led intervention Midwife-led counseling intervention Midwife-led perinatal emotional support intervention Midwife-led mental health intervention	Intervention aiming at women at risk for perinatal mental health issues that is embedded in midwifery care and performed by midwives.	Focus on mental health care. Embedded in midwifery care. Performed by midwives.	Fragmentation of care. More easily implemented within midwifery caseload models, highlighting continuity of care by a known midwife as an enabler. Expansion of the scope of practice for midwives and enhanced service integration and collaboration between mental health and maternity services.	Mental health around childbirth.	Interventions where midwives were not involved
35	Dai, 2024, China	Midwifery-led mHealth app intervention	Mobile health (mHealth) refers to the use of mobile devices	App to reach pregnant women with midwifery information for health promotion and physiological	The mHealth apps should be user-centred (both women and care providers), focusing on users' preferences and demands.	Self-management, health and less anxiety for birth.	Conventional care with women coming to the hospital, seeing the obstetrician.

# in ref. list	First author, year, country	Exact naming of the intervention*	Definition	Attributes	Antecedents	Consequences	Boundaries
			to promote physical health and self-management, reduce anxiety, increase sources of maternal knowledge.	birth. Midwives were involved in the development.			
36	Dawson, 1999, UK	Midwifery intervention	Domiciliary midwifery support delivered by midwives in high-risk pregnancy incorporating telephonic fetal heart rate monitoring	Domiciliary fetal monitoring supported by midwives.	Special training for experienced midwives	Depression, anxiety, satisfaction and costs.	Conventional care with women coming or in the hospital.
37	De Wolff, 2019/2021, Denmark	Midwife-coordinated maternity care intervention (ChroPreg)	An intervention delivered to pregnant women with pre-existing chronic medical conditions by midwives.	Midwife-coordinated and individualized care.<br>Additional ante- and postpartum consultations.<br>Specialized known midwives.	Training for midwives.	Days of hospitalization.<br>Psychological well-being.<br>Satisfaction with care.	Care by obstetrician
10	Endqvist, 2017, Sweden	A multifaceted midwifery intervention	Intervention to reduce second-degree tears in primiparous women.	Initiated, developed and performed by midwives.<br>Consists of 1) spontaneous	Training for midwives.	Prevalence of (second-degree) tears, episiotomies.	-

# in ref. list	First author, year, country	Exact naming of the intervention*	Definition	Attributes	Antecedents	Consequences	Boundaries
				pushing, 2) all birth positions with flexibility in the sacro-iliac joints, and 3) a two-step head-to-body delivery was compared with standard care.			
38	Evans, 2020, UK	A midwife facilitated intervention	Intervention specifically designed to support pregnant women with mild to moderate anxiety.	Initiated(?) and developed and performed by midwives and midwifery support workers.	Training of midwives.	Anxiety during pregnancy.	-
39	Evans, 2022, UK	A midwife-led intervention RAPID intervention	Intervention specifically designed to support pregnant women with mild to moderate anxiety.	Performed by midwives and midwifery support workers. Midwives were involved in developing the intervention.	Training of midwives.	Anxiety during pregnancy.	Midwives without special training can feel uncomfortable as they feel lack of expertise in providing care to women with mental health problems.
40	Evans, 2022b, UK	A midwife facilitated intervention RAPID intervention	Intervention providing suitable, timely support for women with mild-moderate anxiety.	Social support, relational continuity, psychological and relaxation response theory,	Training of midwives.	Anxiety during pregnancy.	-

# in ref. list	First author, year, country	Exact naming of the intervention*	Definition	Attributes	Antecedents	Consequences	Boundaries
				midwife facilitated group discussions, one-to-one support, directed self-help materials.			
41	Fenwick, 2011, Australia	A midwife-led counselling intervention	An intervention promoting resilience in mothers emotions' (PRIME), on anxiety responses and depression in childbearing women.	Performed by midwives.	-	Understanding the source of emotional distress, facilitating a return to equilibrium. Emotional wellbeing in women reporting a traumatic birth experience.	-
42	Fenwick, 2013, Australia	A midwife-led psycho-education intervention	BELIEF: an intervention for reducing women's fear during pregnancy.	Performed by midwives. Midwives were involved in developing the intervention. Responding to women's concerns by psycho-education.	Training of midwives.	Fear of childbirth.	-
43	Fenwick, 2015, Australia	A midwife psycho-education intervention	BELIEF: an intervention for reducing women's fear during pregnancy.	Performed by midwives. Midwives were involved in	Training of midwives.	Fear of childbirth.	-

# in ref. list	First author, year, country	Exact naming of the intervention*	Definition	Attributes	Antecedents	Consequences	Boundaries
				developing the intervention.<br>See: Fenwick 2013.			
44	Fernandez, 2019, UK	Midwifery continuity of care intervention	Continuity of care is defined as delivering care that acknowledges that a woman's health needs are related to events and should be managed over time [6].	Performed by midwives.<br>Midwives were involved in the initiation and development of the intervention.<br>It allows women to develop a relationship with the same caregivers throughout pregnancy, birth and postnatal.<br>Women have a named and backup midwife.	Women see the same midwife throughout the perinatal period.	Preterm birth.	Non-continuous care
45	Firouzan, 2020, Iran	Midwife-led psycho-educational intervention	An intervention delivered by midwives to reduce fear of childbirth.	Performed by midwives.<br>Midwives were involved in developing the intervention.<br>See: Fenwick 2013.	Training of midwives.	Fear of childbirth.	-
46	Gamble, 2005, Australia	A midwife-led brief	An intervention where midwives counsel postpartum	Performed by a midwife.	Training and supervision of the midwife counselling.	Women's mental health and	Intervention requiring sophisticated

# in ref. list	First author, year, country	Exact naming of the intervention*	Definition	Attributes	Antecedents	Consequences	Boundaries
		counseling intervention	women at risk of developing psychological trauma symptom.	Midwives were involved in the initiation and development of the intervention. Elements of critical stress debriefing and issues pertinent to the childbearing context.		symptoms of trauma after birth.	psychotherapeutic skills.
47	Gamble, 2017, Australia	Midwife psycho-education intervention	An intervention delivered by midwives to reduce fear of childbirth.	Performed by midwives. Midwives were involved in developing the intervention. See: Fenwick 2013.	Training of midwives. Learning culture and positive attitude of staff. Time and fitted workload.	Fear of childbirth	-
9	George, 2018, Australia	Midwifery intervention Midwifery initiated oral health-dental service	An intervention initiated by midwives to promote oral hygiene during pregnancy.	Midwives providing oral education, oral health screening, dental referral.	Training of midwives.	Use of dental services. Women's oral health knowledge. Quality of oral health and outcomes.	Dental intervention with assessment and treatment.
48	Gonzalez-Plaza, 2021, Spain	Midwife counselling intervention	A complex digital health intervention, using a smart band and app with midwife counseling, on GWG and	Performed by a midwife. Use of information and communication technologies (ICTs).	Instruction on use of Mi Fit app and the smart band.	Gestational weight gain and total physical activity.	Interventions without use of midwife counselling.

# in ref. list	First author, year, country	Exact naming of the intervention*	Definition	Attributes	Antecedents	Consequences	Boundaries
			physical activity (PA) in women who are pregnant and have obesity.				
49	Gu, 2021, China	Midwifery-led task shifting interventions	Midwife-led services for Chinese pregnant women by shifting tasks in maternity care towards midwives.	Care performed by midwives. Midwife-led model of care based on the premise that pregnancy and childbirth are, in the main, normal life events.	Clear midwifery-led task list.	Physiological childbirth. Women's satisfaction.	Obstetric intervention
50	Heins, 1990, USA	Nurse-midwifery intervention	Care given by a nurse-midwife (rarely a nurse) to women with a high risk for low birth weight.	Nurse-midwife saw the women at risk throughout pregnancy and performed the educational intervention.	Caseload. Supervision.	Use of risk assessment. Low birth weight.	
51	Hodnett, 2008, North America and UK	Complex nursing and midwifery intervention to support normality in birth	Nursing or midwifery care or a minimum of one hour of care by a nurse or midwife trained in structured care when entering the hospital intrapartum.	Performed by nurse or midwife. A formalized approach to assessment of and interventions for maternal emotional state, pain, and fetal position.	A group of nurses and midwives at each hospital were trained in the structured approach. A list of components of structured care.	Spontaneous vaginal birth. Staff helpfulness.	

# in ref. list	First author, year, country	Exact naming of the intervention*	Definition	Attributes	Antecedents	Consequences	Boundaries
52	Homer, 2013, Australia	The intervention: midwifery continuity of care	Women have a midwife caring for them during labour and birth whom they have met before and feel that they know, and this trusting relationship increases their confidence.	Midwives performing the intervention. Midwife is known to the woman. A trusting relationship between woman and midwife.	Earlier contacts with the midwife during pregnancy.	Regional analgesia, instrumental birth, CS, intrapartum analgesia/ anaesthesia, spontaneous vaginal birth, control during labour and childbirth, initiate breastfeeding.	Care without continuity. Care with other professionals than midwives.
53	Huang, 2023, China	A midwife-led Internet + continuous midwifery service model (midwifery service intervention)	The intervention offers maternal management from prenatal to postpartum, in-hospital to out-of-hospital, and offline to online.	Performed by midwives at all stages.	Expertise of midwifery specialist nurses, maternal and infant specialist nurses, and neonatal specialist nurses.	Psychological mood and self-efficacy.	Routine obstetric management mode
54	Hulst, 2004, NL	(1) Midwife technological interventions; (2) Midwife management interventions	Not given, explained by examples: (1) midwife technological interventions: sweeping of membranes, amniotomy, and episiotomy; (2) midwife management interventions:	Conducted by a midwife without involvement of an obstetrician.	Unclear	Unclear	Obstetrician's interventions

# in ref. list	First author, year, country	Exact naming of the intervention*	Definition	Attributes	Antecedents	Consequences	Boundaries
			consultation with an obstetrician without referral, and referral due to existing or anticipated problems for care under supervision of an obstetrician.				
55	Jimenez, 2023, Spain	A low-intensity, midwife-led e-health intervention	An intervention to reduce anxiety during pregnancy.	The intervention will consist of an Immersive Virtual Reality (IVR) application with virtual reality goggles Oculus GO, and uses mindfulness techniques based on breathing, mindfulness and passive muscle relaxation. Giving support and empowerment of the women through low-intensity interventions.	Midwives within the multidisciplinary team have a fundamental role in the detection of women presenting a perinatal mental health problem.	Improved mental health improvement in pregnant women.	-

# in ref. list	First author, year, country	Exact naming of the intervention*	Definition	Attributes	Antecedents	Consequences	Boundaries
56	Khademioore, 2023, Iran	Tele-midwifery intervention	The intervention is an interactive mHealth application based on education and continuous support provided by midwives on FOC, childbirth self-efficacy, and birth mode in primiparous women.	Provided by midwives. Educational component with information and exercises to increase women's knowledge and challenge their underlying beliefs causing the FOC. Continuity of care component for support between two prenatal visits. Participants could contact the researchers (midwives and obstetricians) if they had concerns and questions.	-	Fear of childbirth, self-efficacy of women.	-
57	Khan, 2023, global	Midwifery models of care intervention	Midwifery models of care were defined by the review team as interventions with midwives, or those similarly qualified based on	Midwives providing the care and coordinating it. Continuity from the care provider, and/or care shared in a caseload.	Being trained as a midwife.	Maternal mortality. Perinatal mortality. Experiences including being informed and having information	Interdisciplinary care or community-centred care.

# in ref. list	First author, year, country	Exact naming of the intervention*	Definition	Attributes	Antecedents	Consequences	Boundaries
			the setting, as the central care providers or coordinators of care.	An interpersonal and holistic approach.		explained, having time in their appointments and access to their midwife, having trust and being treated with respect as well as family centred social support, knowing who their care provider was.	
58	Kwegyir-Afful et al., Finland 2018	A midwife-led 3-component liftless intervention	The midwife-led 3-component liftless intervention is based on the clinical guidelines of occupational lifting in pregnancy.	Provided by midwives (ultrasound by obstetricians), including health education, take-home reminder card and a shopping voucher.	All participating midwives receive specific training.	Incidence of preterm birth and low birthweight.	-
59	Lugina, 2001, Sweden	Nursing /midwifery intervention (to be developed)	First step towards an intervention that decreases worry and increases interest and confidence of mothers postpartum.	Not clear (yet), midwives seem the intended group to perform this.	Learning more from women about the topic was important from the aspect of directing nursing and midwifery intervention	Worry and confidence.	-

# in ref. list	First author, year, country	Exact naming of the intervention*	Definition	Attributes	Antecedents	Consequences	Boundaries
60	Lundgren, 2020, Sweden	MiMO (midwifery model of woman-centred care) intervention	A theoretical midwifery model of woman-centred care (MiMo) developed in a Nordic context. The development of the model was informed by women's and midwives experiences.	Initiated, developed and performed by midwives. Three central intertwined dimensions; the midwife is with the woman using grounded knowledge, forms a reciprocal relationship, and creates a birthing atmosphere. These three central dimensions are conducted by the midwife through a balancing act in a cultural context with comprises promoting and hindering norms for conducting a woman-centred care.	One-day (8 h) education day for midwives, who should work with the model in practice. One-hour meeting to introduce the model to each group of obstetricians, assistant nurses and managers. MiMo midwives as "ambassadors" and role models for the other midwives in understanding and using the model on the ward.	Women's satisfaction of birth and interventions during the birth.	-
61	Maga, 2023, global/Italy	Midwifery intervention	Midwifery interventions are defined as elements of maternity care	Provided by midwives. Aiming to improve health outcomes of	Trained midwives.	Health of women, newborns and public health of society.	Everything in maternity care that cannot be done by a midwife.

# in ref. list	First author, year, country	Exact naming of the intervention*	Definition	Attributes	Antecedents	Consequences	Boundaries
			provided by midwives to improve and optimize the health outcomes of women, newborns, and the public health of society at large	women, newborns and public health of society.			
62	Mannocci, 2022, Italy	Midwifery intervention	Intervention to increase maternal self-efficacy and reduce stress.	Performed by midwives – sometimes others: nurses, job-infant care workers and students in obstetrics. Includes educational and mindfulness training and simulations of typical events.	Training course.	Maternal self-efficacy and mood control. Confidence and stress.	
63	Maslin, 2004, global	Nursing and midwifery interventions	Interventions that demonstrates the contribution of nursing and midwifery to the provision of cost-effective, quality health care.	Nurses or midwives performing them.	-	Quality health care. Morbidity and mortality.	-

# in ref. list	First author, year, country	Exact naming of the intervention*	Definition	Attributes	Antecedents	Consequences	Boundaries
64	McGiveron, 2015, UK	A midwife-led intervention	An antenatal weight management intervention comprising a one-to-one programme involving pregnant women with specialist midwives or healthy lifestyle advisors.	One-on-one programme Initiated? And designed by two Healthy Lifestyle Midwife leads. Performed by specialist midwife (or healthy lifestyle advisor).	Not clear	Gestational weight gain.	-
65	McNeill, 2012, economically developed countries	Midwifery intervention Midwifery public health interventions	Public health interventions during pregnancy and postnatal that can be implemented by midwives. But also by care providers that similar roles.	Could be conducted by midwives.	Not clear	Overview of midwifery public health interventions and gaps.	
66	Meedya, 2010, global	Midwifery intervention A midwife-provided educational intervention	First step towards an intervention that strengthens modifying factors for breastfeeding duration up to 6 months postpartum.	Midwives seem the intended group to perform this.	Midwives with special knowledge.	Duration of breastfeeding up to 6 months or more.	
67	Meedya, 2014, Australia	A multiphased midwifery intervention called the Milky Way	An intervention to support women who breastfeeding.	Educational programme. Group sessions Take home learning activities.	Certified lactation consultant (IBCLC) and a midwife supervises the programme.	Breastfeeding duration.	-

# in ref. list	First author, year, country	Exact naming of the intervention*	Definition	Attributes	Antecedents	Consequences	Boundaries
				Postnatal consultations via telephone to support breastfeeding. Initiated, designed and performed by a midwife who is also a certified lactation consultant.			
68	Morlans, 2022, Spain	Midwife-led continuity of care interventions	The main intervention was the establishment of a midwifery consultation including four visits in the antenatal period and one in the postpartum period.	Midwife consultation (antenatal and postnatal) to inform pregnant women individually about the significance of mental wellbeing.	The project had a midwife as lead who was responsible for the promotion of the project, coordination of the team, process control and collecting data. A referral circuit from the obstetrician to the midwife. A midwife full time dedicated to the consultations. Improve knowledge regarding best practice recommendations in perinatal mental health among healthcare professionals who assist pregnant women.	Mental health outcomes in high-risk pregnant women.	-

# in ref. list	First author, year, country	Exact naming of the intervention*	Definition	Attributes	Antecedents	Consequences	Boundaries
69	Morrell, 2016, global	Midwifery-led interventions	Interventions to prevent post-natal depression: Interpersonal psychotherapy (IPT)-based intervention. Parent infant interaction. ITP- based intervention, PCA based and CBT based intervention.	The most beneficial interventions appeared to be midwifery redesigned postnatal care.	-	Depression. Parent-infant interaction.	Various definitions of the term – not related to midwifery
70	Nkowane, 2021, global	Midwifery intervention	Intervention that strengthens the workforce of midwifery or that improves the impact of the work of midwives.	Midwives providing the interventions<br>Focus on health and safe care (access to care services, health education)<br>Focus on leadership of midwives	-	Lead to improved access to health care services through the creation of links among the public, nongovernmental and private sectors to minimize barriers obstructing access to health services for vulnerable populations in urban, rural and remote areas	-
71	Ogrodniczuk, 2003, global	Midwife(-type) intervention	Interventions provided by midwives to prevent	The most effective midwife interventions did	Risk of postnatal depression	Postnatal depression.	Biological intervention.

# in ref. list	First author, year, country	Exact naming of the intervention*	Definition	Attributes	Antecedents	Consequences	Boundaries
			postnatal depression.	not appear to be highly intensive or to differ much from usual midwife care. The new midwife interventions were tailored to the individual needs of the woman and allowed women to express their emotions. An additional difference is that the most effective midwife interventions emphasized continuity of care, which meant that one midwife was responsible for attending all scheduled visits with the woman.			
72	Panda, 2014, Ireland	Midwifery interventions in early labour	Interventions performed by midwives when women present in the antenatal ward	Performed by midwives.	-	-	Not clear.

# in ref. list	First author, year, country	Exact naming of the intervention*	Definition	Attributes	Antecedents	Consequences	Boundaries
			at the start of labour.				
73	Perez-Martinez, 2019, Spain	Midwives' intervention	Puerperal health education provided to women in the hospital by midwives individually on a daily basis during the clinical rounds and at discharge.	Performed by midwives, who were involved in the initiation(?) and development of the protocol.<br>Health education provided to postpartum women.	Competent and trained midwife.<br>Low-risk postpartum women.	Use and cost-benefit of resources.<br>Empowerment of women.<br>Complications.<br>Breastfeeding.<br>Bonding with baby.	Puerperal care by obstetricians.
74	Petersen, 2011, Germany	Interventions applying midwifery care techniques	The presence of the midwife attending the woman in labour was considered an interventions as well as using midwifery care techniques such as vertical positioning.	Midwife present at birth.	-	Interventions applying midwifery care techniques such as vertical positioning preceded more invasive medical interventions during the process of childbirth.	Medical interventions.
75	Polanska, 2004, Poland	The midwife-assisted smoking cessation intervention	An intervention where midwives helped pregnant women to quit smoking.	A midwife performed the intervention.	Midwives visiting women in their home.<br>Information adapted to the cultural conditions.	Smoking<br>Birth weight of infants.	-

# in ref. list	First author, year, country	Exact naming of the intervention*	Definition	Attributes	Antecedents	Consequences	Boundaries
				Midwife gave information and motivation.			
76	Ray, 2004, USA	Midwife and traditional birth attendant-based interventions.	Interventions performed by midwife and traditional birth attendant to improve maternal mortality focus on birth.	The interventions employed traditional birth attendants and/or village midwives at a community level; that is, outside health facilities. Focus on improved management of labour and delivery.	Training of midwives and traditional birth attendants.	Decrease in maternal mortality.	-
77	Rodríguez, 2024. Spain	A midwife-led Breastfeeding Group Intervention	Group interventions during the postpartum period to prevent postpartum depression.	In-person and/or virtual group sessions with midwife in the roles of leader and moderator, including educational element and motivational and social or peer support components established within the group.	Midwives performing the intervention underwent prior training and received guidance from a research technician midwife.	Breastfeeding rates, postpartum depression and general self-efficacy.	-

# in ref. list	First author, year, country	Exact naming of the intervention*	Definition	Attributes	Antecedents	Consequences	Boundaries
78	Sigurðardóttir, 2023, Iceland	A postpartum midwifery counselling intervention	Intervention initiation(?) and developed and offered by midwives to assist women to process a negative birth experience.	The intervention consists of two components based on results from prior studies of writing (and face-to-face counselling interview with a known midwife women from antenatal care, based on active listening and cognitive behavioral approaches. Consideration for women's thoughts, expectations and deep emotions (in a writing session). Perform the intervention tailored to the individual woman ´s needs.	12 h training program (phase I). Educational material contained communication skills and counselling strategy, emotions and physiology of birth, evidence of prevalence, risk factors and consequences of negative birth experience, post-traumatic stress disorder symptoms, case studies and videos.	Well-being of women.	-
79	Simpson, 2016, global	Midwifery-led intervention	Interventions that offer emotional support from midwives during the	Midwives performing the intervention. Midwives giving emotional support.	Training midwives.	Mental health. Feelings of being cared for, safety and wishes respected.	-

# in ref. list	First author, year, country	Exact naming of the intervention*	Definition	Attributes	Antecedents	Consequences	Boundaries
			antenatal and postpartum period.	Emotionally intelligent care. Improved interaction between midwives and women.		Continuity of care.	
80	Smoke, 1988, USA	Nurse-Midwifery Intervention	Care coordinated by a USA-certified nurse-midwife.	Midwife was involved in the initiation(?) and development of the intervention and coordinating the care (with support of other expertise). Midwife doing the assessment of each antennal check-up.	-	Knowledge. Hematocrits, medication while in labor, maternal and fetal complications, blood transfusions, and infant hospital days.	Care by a staff physician and registered or licensed practical nurse.
81	Souto, 2020, global	Midwife intervention	Intervention to reduce fear of childbirth in pregnant women. Originally, it was called midwifery intervention. In an erratum (2021) this was changed to midwife intervention.	Provided by a midwife. Broadens the knowledge of midwives. Prioritize person-centered health and well-being - not just the prevention of death and morbidity - according to evidence-based		Health and well-being – not just the prevention of death and morbidity.	Not provided by midwives. A midwife is a person who has successfully completed a midwifery education programme that is based on the ICM Essential Competencies for Basic Midwifery Practice and the framework of the ICM Global Standards for Midwifery

# in ref. list	First author, year, country	Exact naming of the intervention*	Definition	Attributes	Antecedents	Consequences	Boundaries
				practice and the human rights–based approach. Highlights the concepts of positive pregnancy and childbirth experience as key aspects of women-centered health care and their families.			Education and is recognized in the country where it is located; who has acquired the requisite qualifications to be registered and/or legally licensed to practice midwifery and use the title 'midwife'; and who demonstrates competency in the practice of midwifery.
82	Souto, 2021, erratum	Midwife intervention	In an erratum (2021) this was changed to midwife intervention with the following motivation: "The term ''midwife intervention'' indicates that the intervention is conducted specifically by a health professional with the title of ''midwife,'' which is the population and concept of interest."				

# in ref. list	First author, year, country	Exact naming of the intervention*	Definition	Attributes	Antecedents	Consequences	Boundaries
83	Souto, 2022, n/a	Midwives' intervention	All interventions that included a midwife or team of midwives who worked independently or within a multidisciplinary team to reduce fear of childbirth.	Midwives involved in providing the intervention (also including others), often in a leading role. Intervention integrated in the care practice by midwives. Most interventions included consideration for emotions and easy contact with midwife.	Health professionals' training (including, but not limited to, midwives) and expertise, and the strategies for supervising/assessing the interventions.	-	Interventions provided by other than midwives. The midwife is recognized as a responsible and accountable professional who works in partnership with women to give the necessary support, care and advice during pregnancy, labour and the postpartum period, to conduct births on the midwife's own responsibility and to provide care for the newborn and the infant … has an important task in health counselling and education.
84	Spindler, 2018, India	A nurse midwife mentoring intervention	An intervention using debriefing after births to improve skills and knowledge of midwives.	Targeted at nurse midwives.		Quality of care. Skills and knowledge.	
85	Swann, 2012, UK	Midwifery intervention	Alternatives to the medicalized	Performed by midwives.	Can be advocated in all birth environments.	Rates of normal birth.	Medicalized interventions.

# in ref. list	First author, year, country	Exact naming of the intervention*	Definition	Attributes	Antecedents	Consequences	Boundaries
			interventions to promote normal birth.	Positive interventions.			
86	Taylor Miller, 2021, global	Midwifery-led intervention Midwifery led brief counselling intervention	Intervention targeting post-traumatic stress disorder performed by midwives.	Midwife performing the intervention.	-	-	Clinician-led interventions.
87	Toohill, 2017, Australia	A midwife-led psycho-education intervention	Not given. See: Fenwick, J., et al., 2013.	Midwife performing the intervention. Psycho-education.	Training of midwives.	Vaginal birth. Treatment costs. Health services costs.	-
88	Toohill, 2019, Australia	A midwife-led psycho-education intervention	Not given. See: Fenwick, J., et al., 2013.	Midwife performing the intervention. Psycho-education.	Training of midwives.	Access and use of care.	-
89	Truva, 2021, Greece	Midwifery intervention programme	A personalized prenatal and postnatal structured midwifery intervention programme on increasing breastfeeding rates provided by midwives.	Personalized approach. Educating and empowering women. Provided by a midwife.		Individualized structured prenatal and postnatal breastfeeding education by a specialty midwife, breastfeeding frequency in women.	-
90	Türkmen, 2021, Turkey	Midwifery intervention	An intervention using thermoforming and	Initiated, developed and provided by a midwife.	-	Reduced labour pain.	-

# in ref. list	First author, year, country	Exact naming of the intervention*	Definition	Attributes	Antecedents	Consequences	Boundaries
			massage given by midwives.				
91	Türkmen, 2023, Turkey	Midwifery intervention	An intervention using rotational ice massage on pregnant women given by midwives.	Provided by a midwife.	-	Reduced labour pain.	-
92	Turkstra, 2017, Australia	A brief antenatal midwifery psycho-education intervention	The intervention addressing women's expectations and feelings around fear of childbirth.	Performed by midwives. Psycho-education.	Midwives were trained.	Childbirth fear according to the Wijma Delivery Expectancy/ Experience Questionnaire version A (WDEQ-A).	-
93	Wallace, 2006, UK	Midwifery intervention	No definition The protocol required that the intervention was delivered by midwives. Midwives in the midwifery intervention group attended a 4-hr long workshop covering the rationale and skills of a 'hands off' approach to care at first feed. Verbal-only care was	This midwifery intervention should be delivered by a midwife and emphasized a 'hands off' approach that offered the mothers a chance to attach the baby herself.	Training covering the rationale and skills of a 'hands off' approach to care at first feed.	No difference in primary outcome (duration of breastfeeding) compared to other strategies.	-

# in ref. list	First author, year, country	Exact naming of the intervention*	Definition	Attributes	Antecedents	Consequences	Boundaries
			advised to ensure the mother was able to attach the baby herself. Midwives were part of the research team and involved in the development of the intervention.				
94	Wang, 2021, China	Midwives-led psychological intervention	Interventions provided by midwives on depressive symptoms in perinatal women.	Conducted by midwives.	-	Depression symptoms.	-
95	Wang, 2023, China	A midwife-led weight management program	A midwife-led weight management program that facilitates appropriate gestational weight gain for pregnant women, enhances their health literacy during pregnancy, and promotes their experience of antenatal care.	The intervention was conducted by trained midwives. It was developed with experts, including experts from the field of midwifery and others.	Midwives were experienced and specifically trained. Women had to attend midwife's antenatal clinic for 5 contacts.	Gestational weight gain. Health literacy. Experience of antenatal care.	-

# in ref. list	First author, year, country	Exact naming of the intervention*	Definition	Attributes	Antecedents	Consequences	Boundaries
96	Warren, 2017, UK	A brief midwife-led intervention	An intervention to facilitate healthful dietary and physical activity behaviours in pregnant women, the Eat Well Keep Active' intervention programme.	Initiated, developed and performed by a midwife. Relying on improvement of women's motivation, reinforcing personal responsibility and supporting self-efficacy.	Pregnant women suitable for Midwife Led Care and therefore deemed to be 'low risk'.	Overweight and obesity in women during pregnancy.	-
97	Wei, 2021, China	Midwife intervention nursing mode during birth (combined with acupressure)	An obstetric nursing mode dominated by midwives is more humanized and targeted to promote vaginal birth versus routine obstetric care.	One-to-one midwife outpatient consultation services. Introducing pregnant women, parturients and their families to the safety and feasibility of spontaneous vaginal delivery in the scarred uterus and answering all questions and doubts of pregnant and parturient women so as to improve their	Midwives should fully understand the needs of parturients, immediately reassess the condition for vaginal delivery and provide corresponding intervention, such as psychological counselling and relieving uneasy, anxious and other emotions of parturients.	Vaginal delivery rate, rates of lateral episiotomy, puerperal infection and neonatal asphyxia. Duration of first stage of labour and second stage of labour and postpartum 2 h and 24 h haemorrhage. Visual analogue scale scores for pain in the incubation period and active period and for negative emotion (Hamilton anxiety (HAMA) scale,	*The principle of "stop" was always followed during the labour process, if parturients decided to give up spontaneous vaginal delivery.*

# in ref. list	First author, year, country	Exact naming of the intervention*	Definition	Attributes	Antecedents	Consequences	Boundaries
				confidence to select spontaneous vaginal delivery; daily popularizing the significance of self-management in prenatal weight, diet and others; strengthening pregnant and parturient women to identify abnormal manifestations and fully know normal pregnant performances so as to perceive abnormal condition timely; introducing pregnant and parturient women to the way to labor during parturition and auxiliary labor methods like Lamaze breathing method and anodyne labor;		Hamilton depression (HAMD) scale and nursing satisfaction.	

# in ref. list	First author, year, country	Exact naming of the intervention*	Definition	Attributes	Antecedents	Consequences	Boundaries
				psychological counseling to establish the confidence of pregnant and parturient women in spontaneous vaginal delivery, give faith support and release negative emotions; providing corresponding assessment based on the individual condition of pregnant and parturient women and health education. Parturients were guided to use free position and birthing ball for relieving pain caused by uterine contraction and were advised to select epidural anesthesia for			

# in ref. list	First author, year, country	Exact naming of the intervention*	Definition	Attributes	Antecedents	Consequences	Boundaries
				painless labor. Indications for vaginal midwifery were relaxed properly during the second stage of labor, and abdominal pressure was forbidden. The midwife visited parturients on the first day after delivery to assess the condition of uterine involution, puerpera mentality and breast feeding, and to provide necessary psychological counseling and postpartum rehabilitation training.			
98	Wilkinson, 2016, UK	Midwife-led group intervention	An intervention tailored specifically for use with pregnant women experiencing antenatal anxiety,	Brief. Being performed by midwives. Embedded in routine antenatal care.	A trained midwife and psychology supporter.	Maternal anxiety. Maternal mood (depression). Pregnancy specific worries.	-

# in ref. list	First author, year, country	Exact naming of the intervention*	Definition	Attributes	Antecedents	Consequences	Boundaries
			based on the principles of Cognitive Behavioural Therapy (CBT), for use in the UK in a group setting led by midwives.	Tailored specifically for pregnant women experiencing antenatal anxiety.			

Supplement 4: Data extraction table II with analytical questions for concept analysis of Midwifery Intervention

By answering the analytical questions we aim to identify papers for the second part of our result section that present good examples of midwifery interventions.

- *The papers marked green are papers that present model casus relevant for further analysis in the second part of the paper:*
    - *C1: clearly described that midwives were involved in the development of the intervention*
    - *C2: clearly described that midwives coordinate/provide the delivery of the intervention*
    - *C3:The intervention relates to the ICM midwifery philosophy of care, implying in the text that it meets 7-8 of its components.*
- *The light green papers come close but are not fully meeting all criteria (related casus).*
- *The papers marked orange were assessed as less relevant for the second part of the paper.*
- *The question "How does the intervention relate to the ICM midwifery philosophy of care?" is an analytical question, where we as authors analyse the intervention as presented in the paper and decide if it meets the criteria of the midwifery philosophy of care.*
- *Systematic reviews (SR) were excluded.*

# in ref. list	First author, year, country	What is the theoretical background of the intervention?	How was the intervention developed?	What does the intervention look like and how is it used?	How is the intervention specifically connected to midwifery?	How does the intervention relate to the ICM midwifery philosophy of care?*	C1	C2	C3
18	Abhari, 2020, Iran	Treatment of postpartum psychological disorders mostly involves a combination of psychotherapy, support, and psychiatric medication therapy. Since the	The counseling strategy developed by Gamble et al. could be adopted by obstetricians and midwives. It is notable that Gamble's counseling approach is not a psychotherapeutic	In the intervention group, individual counseling based on Gamble's approach was provided by a one midwife (researcher). The strategies adopted to provide the counseling	Midwives were involved in developing and providing the intervention.	ICM philosophy: 2,3, 5-8	+	+	-

# in ref. list	First author, year, country	What is the theoretical background of the intervention?	How was the intervention developed?	What does the intervention look like and how is it used?	How is the intervention specifically connected to midwifery?	How does the intervention relate to the ICM midwifery philosophy of care?*	C1	C2	C3
		treatment is often initiated following the development of the symptoms, and as psychiatric medications could give rise to complications in breastfeeding women, nonmedicinal, preventative, prenatal interventions should be considered during pregnancy and before the development of the disorder [30,31]. Considering that psychological birth trauma is a mental process contingent upon the	counseling approach, but rather a preventive technique. See: Fenwick, 2013	intervention included the establishment of a therapeutic relationship between the midwife and parturient woman, acceptance of the maternal perceptions regarding labor, supporting the mother in expressing her emotions, resolving the ambiguities for the mothers, establishing connections between behaviors, emotions, and delivery, reviewing the phases and modes of labor,					

# in ref. list	First author, year, country	What is the theoretical background of the intervention?	How was the intervention developed?	What does the intervention look like and how is it used?	How is the intervention specifically connected to midwifery?	How does the intervention relate to the ICM midwifery philosophy of care?*	C1	C2	C3
		perceptions of women toward labor and delivery [26,32], it could be prevented by analyzing the viewpoints of pregnant women, as well as their mental support and listening to their experiences [2,33].		fostering social support, facilitating maternal adaptation, promoting positive maternal thoughts, and finding solutions. The counseling was provided in three prenatal sessions, including two 45–60-min face-to-face sessions in weeks 35 and 37, one 10–15-min phone counseling session in week 36 of gestation, and one 45–60-min face-to-face postpartum counseling session 4–12 h after labor.					

# in ref. list	First author, year, country	What is the theoretical background of the intervention?	How was the intervention developed?	What does the intervention look like and how is it used?	How is the intervention specifically connected to midwifery?	How does the intervention relate to the ICM midwifery philosophy of care?*	C1	C2	C3
19	Abou Malham, 2015, Morocco	Hatem-Asmar's conceptual model was used to delineate the context through which an intervention needed to be disseminated across the dimensions (values, actors, methods, and targets) of the three interacting systems (sociocultural, professional, and educational) in order to change a health professional's role.	Morocco has been experimenting, since 2008, with setting up a "strengthening midwifery" action plan proposed by a consultant (Hatem, unpublished data, 2008). It was implemented as part of a national health strategy's core action, which focuses on strengthening human resources competencies.	It was designed by a consultant based on a conceptual framework stipulating that three systems (sociocultural, educational, and professional) are crucial and should be targeted by an intervention aiming to change a professional role. Consequently, the conceptual foundations for designing the intervention led to an operational action plan comprising nine objectives and relevant activities. In fact, the action	Midwives were the intervention.	ICM philosophy: 3	-	+	-

# in ref. list	First author, year, country	What is the theoretical background of the intervention?	How was the intervention developed?	What does the intervention look like and how is it used?	How is the intervention specifically connected to midwifery?	How does the intervention relate to the ICM midwifery philosophy of care?*	C1	C2	C3
				plan took a wider approach and was aiming at multiple changes rather than only educational changes (eg, curriculum review and implementation of competency-based education [CBE], continuing education program) as initially requested by the human resources department of the MOH. The additional changes suggested to improve the midwifery role, following the diagnostic analysis,					

# in ref. list	First author, year, country	What is the theoretical background of the intervention?	How was the intervention developed?	What does the intervention look like and how is it used?	How is the intervention specifically connected to midwifery?	How does the intervention relate to the ICM midwifery philosophy of care?*	C1	C2	C3
				included changes to the midwifery profile and care provision, the midwifery legislation, and the professional midwifery association and community recognition, as well as changes to the organization of human resources in the Moroccan health system.					
20	Adams, 2017, USA	The physiologic changes that accompany pregnancy may result in oral health problems. Shifts in hormonal, immunologic, and vascular function	The study team, including public health dentists, a periodontist, a behavioral scientist, and Centering-Pregnancy leadership and	The intervention aligns with Centering-Pregnancy principles, and the key oral health messages reflect evidence-based professional	Midwives were the Centering-Pregnancy facilitators. Midwives were involved in developing and providing the intervention.	ICM philosophy: 8<br><br>Possibly meeting some more criteria as they are	+	+	-

# in ref. list	First author, year, country	What is the theoretical background of the intervention?	How was the intervention developed?	What does the intervention look like and how is it used?	How is the intervention specifically connected to midwifery?	How does the intervention relate to the ICM midwifery philosophy of care?*	C1	C2	C3
		can exacerbate gingival inflammation and increase susceptibility to periodontal disease. While gingival inflammation usually subsides after childbirth, if left untreated during pregnancy, gingivitis will likely persist after pregnancy. Although the literature is mixed and recent research has failed to find an association between treatment of periodontal disease and	facilitators, developed the skills-based educational intervention.	guidelines. The content was evaluated as informative and acceptable by Centering-Pregnancy participant focus groups. An earlier oral health curriculum, Centering-Smiles, developed by different researchers for Centering-Pregnancy sites in Kentucky included dental care, oral health topics in each session, and a dental hygienist instructor. Intervention facilitators		using Centering-Pregnancy, but not described.			

# in ref. list	First author, year, country	What is the theoretical background of the intervention?	How was the intervention developed?	What does the intervention look like and how is it used?	How is the intervention specifically connected to midwifery?	How does the intervention relate to the ICM midwifery philosophy of care?*	C1	C2	C3
		adverse birth outcomes, oral health is associated with general health, and maternal oral health can impact infant oral health through postpartum maternal-to-infant transmission of caries-causing bacteria, mutans streptococci, and lactobacilli.		underwent a 3-hour training on intervention content and delivery including didactic information and demonstration. Tool boxes were provided that included the learning objectives, prompts, and reminder instructions for the skills-based activities. Posttraining, facilitators conducted a practice session in a non-study Centering-Pregnancy group and received					

# in ref. list	First author, year, country	What is the theoretical background of the intervention?	How was the intervention developed?	What does the intervention look like and how is it used?	How is the intervention specifically connected to midwifery?	How does the intervention relate to the ICM midwifery philosophy of care?*	C1	C2	C3
				feedback on fidelity.					
21	Ajuebor, 2019, global	In the paper the concept of intervention is defined more widely that is to implement the Global Strategic Directions for Strengthening Nursing and Midwifery. Not explicit how the intervention is developed but theoretical background based on WHO etc.	The intervention has not been fully developed – the first step is outlined – to gain information through questionnaire and interviews.	No information.	It refers to nursing and midwifery workforce. As such not particularly midwifery oriented.	ICM philosophy: none	-	+/-	-
22	Alderdice, 2013, economically developed countries<br><br>SR of SRs								

# in ref. list	First author, year, country	What is the theoretical background of the intervention?	How was the intervention developed?	What does the intervention look like and how is it used?	How is the intervention specifically connected to midwifery?	How does the intervention relate to the ICM midwifery philosophy of care?*	C1	C2	C3
23	Allen, 2016, Australia	Caseload midwifery describes a model of maternity care that focusses on providing the woman with ‘continuity of carer’ from a known midwife through out pregnancy, labour/birth and the postpartum period. The effect of social deprivation on behaviour, health and living conditions are strongly associated with both adolescent pregnancy and PTB; the risk factors are identical.	A young women’s caseload midwifery model started in 2008. No further information.	Each woman was provided with a primary midwife, 24-hour telephone contact with her midwife or back-up, a booking visit in her home, community-based antenatal care that occurred in a group with other young pregnant women, a familiar midwife in labour (one of the four midwives in the group), and postnatal home visiting for 4–6 weeks after birth.	Midwives were providing the care and look at the woman as a whole.	ICM philosophy: 2,3,5-8	+	+	-

# in ref. list	First author, year, country	What is the theoretical background of the intervention?	How was the intervention developed?	What does the intervention look like and how is it used?	How is the intervention specifically connected to midwifery?	How does the intervention relate to the ICM midwifery philosophy of care?*	C1	C2	C3
		Modifying the risk and protective factors inherent in adolescents daily lives, especially for those who are most social-economically disadvantaged, can improve health outcomes. Indeed, programmes targeted to improve the circumstances of socially disadvantaged women can reduce PTB. A Cochrane systematic review of participants enrolled in different models of maternity care found that women							

# in ref. list	First author, year, country	What is the theoretical background of the intervention?	How was the intervention developed?	What does the intervention look like and how is it used?	How is the intervention specifically connected to midwifery?	How does the intervention relate to the ICM midwifery philosophy of care?*	C1	C2	C3
		randomised to midwife-led care, compared to standard care, were less likely to give birth preterm.							
24	Allen, 2017, Australia	Case-load midwifery	It is not fully explained: a number of interacting components that take different forms in different context, midwives were involved in the study team.	Derives from a therapeutic relationship or are relationally mediated.	Midwives were involved in developing and providing the intervention.	ICM philosophy: 2,3,5-8	+	+	-
25	Altiner, 2019, Turkey	Time and motion studies are used to explain multitasking intervention. Also, a description of the Omaha System which is explained as a comprehensive	Information about the development of the content of nurse/midwife multitasked intervention by using Omaha System.	A special TimeCaT was customized for capturing nurse/midwives' interventions.	Some connections to figures in the paper where midwifery interventions are named. Otherwise, it has no connection to midwifery.	ICM philosophy: none	-	+	-

# in ref. list	First author, year, country	What is the theoretical background of the intervention?	How was the intervention developed?	What does the intervention look like and how is it used?	How is the intervention specifically connected to midwifery?	How does the intervention relate to the ICM midwifery philosophy of care?*	C1	C2	C3
		standardized taxonomy designed to document patient care.							
26	Asadzadeh, 2020, Iran	Childbirth can be stressful for women. Researches showed that 34% [1] to 54% [2] of women have experienced childbirth as a traumatic event. This shows the necessity of considering counseling approaches for women who are at risk of developing a psychological distress after giving birth.	See: Fenwick 2015.	It consists of two sessions of counseling by midwives at 48–72 h and four to 6 weeks after giving birth. This intervention emphasizes on the therapeutic relationship, acceptance of experiences, expression of emotions, reviewing labor management, increasing social support, and problem solving [10].	Midwives were involved in developing and providing the intervention.	ICM philosophy: 2,3, 5-8	+*	+	-

# in ref. list	First author, year, country	What is the theoretical background of the intervention?	How was the intervention developed?	What does the intervention look like and how is it used?	How is the intervention specifically connected to midwifery?	How does the intervention relate to the ICM midwifery philosophy of care?*	C1	C2	C3
27	Bick, 2022, UK	The Behaviour Change Wheel theoretical framework and the Behavior Change Technique Taxonomy	The Antenatal Preventative Pelvic floor Exercises And Localisation (APPEAL) programme includes several linked work packages (WP). The Royal College of Midwives and Chartered Society of Physiotherapy published a joint statement recommending that all pregnant women should be offered evidence-based information and advice on PFME. Not clear if midwives were	Midwives introduce the programme to women at booking interview. Women receive a resource package to help them to perform PFME. Videos, apps ect.	The midwife is the one who guides the woman in using Pelvic floor intervention.	ICM philosophy: 3,8	-	+	-

# in ref. list	First author, year, country	What is the theoretical background of the intervention?	How was the intervention developed?	What does the intervention look like and how is it used?	How is the intervention specifically connected to midwifery?	How does the intervention relate to the ICM midwifery philosophy of care?*	C1	C2	C3
			involved in the development.						
28	Blomgren, 2023<br>Sweden	Integrating evidence-based midwifery practices improves healthcare quality for<br>women and newborns. Evidence-based midwifery practices include elements such as providing continuous labour support and promoting upright birthing positions, relaxation techniques and skin-to-skin contact between mothers and babies. [13,14].	Not clear -<br>A qualitative approach using the Consolidated Framework for Advancing Implementation Science (CFIR) was used to guide the co-creation of the intervention (i.e. the co-creation process). Participants who represent multiple vital sectors for creating change within clinical midwifery care according to the Midwize framework were selected,	The study, which seeks to collaboratively design a QI intervention to bridge the gap between evidence and practice in midwifery, outlines four key stages. In the first stage, the core elements of the QI intervention are defined. In the second stage, we evaluated needs and context, underscoring the importance of empowering women in childbirth decisions fostering shared	It is developed by midwives in cooperation with midwives and other (stakeholders/obstetricians)	ICM philosophy: 3,4,5,6,7	+	+	-

# in ref. list	First author, year, country	What is the theoretical background of the intervention?	How was the intervention developed?	What does the intervention look like and how is it used?	How is the intervention specifically connected to midwifery?	How does the intervention relate to the ICM midwifery philosophy of care?*	C1	C2	C3
		The Midwize framework is developed to help guide the selection of stakeholders to include in change processes' in maternal healthcare	convenience sampling was made comprising new mothers and 32 midwives employed at the hospital to participate in one of the three FGDs. Data was used to systematically explore the midwife-led QI intervention's contextual factors, needs, and determinants.	goals within the team, promoting current knowledge exchange at the department, and boosting staff motivation. In the third stage, co-creation workshops identified key priorities for the QI and in the fourth stage, the finalisation of the QI intervention design involved tailoring strategies and assigning roles to Midwize Ambassadors with the aim of transitioning full leadership to this group over time.					
29	Borg Cunen, 2014, global								

# in ref. list	First author, year, country	What is the theoretical background of the intervention?	How was the intervention developed?	What does the intervention look like and how is it used?	How is the intervention specifically connected to midwifery?	How does the intervention relate to the ICM midwifery philosophy of care?*	C1	C2	C3
	SR								
30	Borges, 2021, UK	Case-load midwifery	A discussion about connecting case-load midwifery to cystic fibrosis.	No information.	Midwives were involved in developing and providing the intervention, using midwifery caseload in the care of women with CF.	ICM philosophy: 1-8	+	+	+
31	Borneskog, 2023, Bangladesh	No information	No information	Interventions done when a life is at risk, e.g. resuscitation neonate.	Midwives provide the intervention	ICM philosophy:- 3,8	-	+	-
32	Bryce, 2009, UK	CATCH (a responsive smoking cessation service) Health promotion Motivational interviewing	Basic intervention had been developed previously. The intervention was developed further by adding a midwife with knowledge and experience in family therapy, motivational interviewing and	Consultations, leaflets, telephone calls to follow up. Special sessions for young women and another for women over 25.	Midwives were involved in developing and providing the intervention.	ICM philosophy: 2,3,5,6,8	+	+	-

# in ref. list	First author, year, country	What is the theoretical background of the intervention?	How was the intervention developed?	What does the intervention look like and how is it used?	How is the intervention specifically connected to midwifery?	How does the intervention relate to the ICM midwifery philosophy of care?*	C1	C2	C3
			withdrawal oriented therapy.						
33	Caelli, 2001, Canada and Australia	SDS is based on collaboration of obstetricians, GP and midwives, additional to routine antenatal care. Theoretical background not described in more detail.	Developed in UK and adapted in Canada and Australia by midwives.	Extra visits and group sessions. A holistic approach when providing support and care according to participants.	Midwives were involved in developing, and providing the intervention in collaboration with obstetricians and GPs.	ICM philosophy: 2,3,5-8	+	+	-
34	Coates, 2019 global<br><br>SR								
35	Dai, 2024, China	Studies have shown that maternal health mobile apps can promote physical health and self-management, reduce anxiety, increase sources of maternal	The current study is the start of the development of the intervention, gaining an understanding of women's and healthcare professionals' views on the	It will be a mobile app with information on how to increase women's knowledge about how to promote their health in the antenatal period.	It is necessary to give full play to Chinese midwives' roles and functions since early trimesters to better meet the needs of women. In this context, telemedicine is getting more attention as it can help bridge gaps in healthcare services.	ICM philosophy: 1,3,5,6,8	+	+	-

# in ref. list	First author, year, country	What is the theoretical background of the intervention?	How was the intervention developed?	What does the intervention look like and how is it used?	How is the intervention specifically connected to midwifery?	How does the intervention relate to the ICM midwifery philosophy of care?*	C1	C2	C3
		knowledge and have great potential to increase satisfaction and reduce visit times in areas such as diabetes and psychology.	development of a midwifery-led mHealth app intervention in antenatal care and their demands for app functionality.						
36	Dawson, 1999, UK	Domiciliary based community care.	The study was about assessing a new application of health technology (CTG) in high risk pregnancy. It emphasized domiciliary midwifery support but the intervention was not explained further.	Women had visits from community midwives and a telecare surveillance was used to follow up on their wellbeing..	By emphasizing home based midwifery care and community midwives taking responsibility for domiciliary monitoring.	ICM philosophy: (1,3,5,6),8	-	+	-
37	De Wolff, 2019/2021, Denmark	Care coordination, continuity of care, woman-centered	The three main components of the ChroPreg	The midwife had the role of care coordinator	Provided by a specialized midwife	ICM philosophy: (2),3,5,6,8	-	+	+

# in ref. list	First author, year, country	What is the theoretical background of the intervention?	How was the intervention developed?	What does the intervention look like and how is it used?	How is the intervention specifically connected to midwifery?	How does the intervention relate to the ICM midwifery philosophy of care?*	C1	C2	C3
		care, and specialized maternity care interventions delivered to women with high-risk pregnancies can improve patient-reported outcomes and pregnancy outcomes and be cost-effective. The ChroPreg intervention is designed on the basis of a review of the literature on complex interventions in maternity care [19–21] and is aimed to fit the needs of women with chronic disease.	intervention were: (1) Midwife-coordinated and individualized care, (2) additional ante- and postpartum consultations, and (3) specialized known midwives.	between all health care providers involved in providing maternity care for the women.					

# in ref. list	First author, year, country	What is the theoretical background of the intervention?	How was the intervention developed?	What does the intervention look like and how is it used?	How is the intervention specifically connected to midwifery?	How does the intervention relate to the ICM midwifery philosophy of care?*	C1	C2	C3
10	Edqvist, 2017, Sweden	The intervention is based on women centered care.	Based on literature review of previous studies and the components of women centered care.	1) spontaneous pushing, 2) all birth positions with flexibility in the sacro-iliac joints, and 3) a two-step head-to-body delivery	It is based on the MiMo model of midwifery care.	ICM philosophy: 1-3,5-8	+	+	+
38	Evans, 2020, UK	Two systematic reviews were completed which helped identify potentially beneficial intervention components. The theory underpinning the components was explored to consider the potential benefit for women with mild to moderate	The development followed the MRC framework for complex interventions, utilizing psychological theory, review level evidence and professional and public involvement. Two systematic reviews were completed which helped identify potentially beneficial	The intervention comprised: group discussions, one to one support and assisted self-help resources. Midwives were identified as ideally placed to facilitate the intervention supported by midwifery support workers. A bespoke training package was provided by subject experts to	Midwives provided the intervention.	ICM philosophy: 2, 3, 5, 8	+	+	-

# in ref. list	First author, year, country	What is the theoretical background of the intervention?	How was the intervention developed?	What does the intervention look like and how is it used?	How is the intervention specifically connected to midwifery?	How does the intervention relate to the ICM midwifery philosophy of care?*	C1	C2	C3
		anxiety symptoms in pregnancy. Social support theory Therapeutic relation theory Mind-body approaches Cognitive-behavioural mechanisms Multi-component approach	intervention components. The theory underpinning the components was explored to consider the potential benefit for women with mild to moderate anxiety symptoms in pregnancy. Midwives were involved in the development.	prepare the facilitators.					
39	Evans, 2022, UK	The theory underpinning RAPID includes: 1) Social support theory; 2) Therapeutic relation theory; 3) Mindbody approaches; 4) Cognitive-	Preliminary work was conducted to design and develop the intervention and a small scale study was completed 2016 in an NHS Trust in England (RAPID-1). The design and	The RAPID intervention comprises three components: 1) One-to-one pre-group introductory meeting with the midwife facilitator. 2) Facilitated group discussion sessions.	Midwives were involved in developing and providing the intervention. This was motivated from the wider midwifery care literature, which stressed the need to strengthen the role of the midwife in promoting women's mental and emotional wellbeing [12].	ICM philosophy: 1,2,3,5,6,8	+	+	-

# in ref. list	First author, year, country	What is the theoretical background of the intervention?	How was the intervention developed?	What does the intervention look like and how is it used?	How is the intervention specifically connected to midwifery?	How does the intervention relate to the ICM midwifery philosophy of care?*	C1	C2	C3
		behavioural mechanisms [10]. It is considered that the intervention will promote positive change in women's anxiety symptoms through 1. developing collaborative relationships which aim to promote women's choice and control over their care. 2. receiving support from healthcare professionals (HCPs) who both understand women's individual needs and can also help them access services; 3.	development work, including stakeholder and service user engagement, conceptual and theoretical frameworks and outcome measures has been reported in a previous paper [10].	3) A choice of self-help materials for women to access between groups. The choice of materials is based on service user preferences and relevance in a UK healthcare context (Fig. 1).	Thus, developing an intervention which could be delivered within midwives' scope of practice [13], with minimal additional resources and which could be integrated into midwifery services was of particular importance.				

# in ref. list	First author, year, country	What is the theoretical background of the intervention?	How was the intervention developed?	What does the intervention look like and how is it used?	How is the intervention specifically connected to midwifery?	How does the intervention relate to the ICM midwifery philosophy of care?*	C1	C2	C3
		accessing support and learning from other women who have experienced / are experiencing similar feelings or situations; 4. Developing strategies to help women develop an awareness of their thought processes and learn techniques to improve the way they cope with anxiety.							
40	Evans, 2022b. UK	See Evans 2020: Three components were used to develop a novel intervention, comprising midwife facilitated group discussions, one-to-	Synthesized findings from two reviews identified three components which were likely to increase the effectiveness of the intervention: (1)	Schedule of 12 weeks initial meetings and group sessions facilitated by a midwife	Intervention facilitators are: midwives who have completed preceptorship period and midwifery support workers with a minimum of 1 year's experience in maternity care.	ICM philosophy: 2,3,5,7	-	+	-

# in ref. list	First author, year, country	What is the theoretical background of the intervention?	How was the intervention developed?	What does the intervention look like and how is it used?	How is the intervention specifically connected to midwifery?	How does the intervention relate to the ICM midwifery philosophy of care?*	C1	C2	C3
		one support and directed self-help materials with a choice of cognitive or mindbody resources	social support; (2) relational continuity and (3) psychological and relaxation response theory.						
41	Fenwick, 2011, Australia	Perinatal mental health difficulties are prevalent, likely to recur, become chronic and have long-term consequences for women, their infants, and family. Research to date has primarily focused on postpartum depression with less attention given to anxiety disorders. Preventative strategies or early	No information	No information	No information	No information	-	-	-

# in ref. list	First author, year, country	What is the theoretical background of the intervention?	How was the intervention developed?	What does the intervention look like and how is it used?	How is the intervention specifically connected to midwifery?	How does the intervention relate to the ICM midwifery philosophy of care?*	C1	C2	C3
		interventions targeted towards women at risk of postpartum emotional distress are required.							
42	Fenwick, 2013, Australia	Approaches to assist women reporting childbirth fear have been pioneered in Scandinavian countries. In Sweden, many obstetric departments have established qualified teams to support women who identify as highly fearful of birth.	The counselling intervention 'Promoting Resilience in Mothers' Emotions' (PRIME) [34] will be adapted for use in this study for two reasons; the benefits of the counselling approach has applicability to the target population and it is reproducible. This counselling approach has demonstrated	A brief midwife-led counselling intervention that does not require the midwife to have advanced psychotherapeutic qualifications has been developed and tested with postnatal women and found to reduce postnatal emotional distress [33]. The intervention will occur by telephone. The intervention will be provided	Midwives were involved in developing the intervention. Trained midwives will perform the intervention. The intervention follows the midwifery philosophy of care.	ICM philosophy: 1-3,5-8	+	+	+

# in ref. list	First author, year, country	What is the theoretical background of the intervention?	How was the intervention developed?	What does the intervention look like and how is it used?	How is the intervention specifically connected to midwifery?	How does the intervention relate to the ICM midwifery philosophy of care?*	C1	C2	C3
			statistically significant sustained improvement in women's emotional health and confirmed that women value the opportunity to talk about their birth experience and to have these experiences validated [35].	between 24 and 34 weeks gestation and support women to examine the origin of childbirth fear, reconcile any impact from a previous birth experience, be informed of their birth options and develop strategies for a positive birth experience regardless of the ultimate mode of birth.<br>Education<br>information<br>Support					
43	Fenwick, 2015, Australia	See: Fenwick 2013	See: Fenwick 2013	See: Fenwick 2013	See: Fenwick 2013	See: Fenwick 2013	+	+	+

# in ref. list	First author, year, country	What is the theoretical background of the intervention?	How was the intervention developed?	What does the intervention look like and how is it used?	How is the intervention specifically connected to midwifery?	How does the intervention relate to the ICM midwifery philosophy of care?*	C1	C2	C3
44	Fernandez, 2019, UK	This trusting relationship may increase their confidence and reduce levels of psychosocial stress [18, 19]. This close relationship between women and midwives may also help women feel that they have a health care provider who knows their medical history who will be caring for them throughout labour, birth and the postnatal period [20, 21]. The trust and feeling of safety that this may afford may mean	Does not say in detail but refers to the project ´Better births'. The research [10–12] provides a basis to develop and test the impact of a novel care pathway for women at increased risk of PTB, which combines continuity of midwife care with rapid referral to a specialist obstetric clinic throughout pregnancy through to the intrapartum and postpartum periods [13]. The midwifery intervention will be provided from	Women allocated to the intervention group will receive a continuity of midwife care model during the antenatal, labour, birth and postnatal periods, predominantly from one midwife and her partner midwife (backed up by the rest of the POPPIE team). Midwives will 'follow women' and scheduled and out of hours' antenatal, intrapartum and postnatal care may be provided in hospital, community/ children's centres	Midwives were involved in developing and providing the intervention.	ICM philosophy: 3,5-8	+	+	-

# in ref. list	First author, year, country	What is the theoretical background of the intervention?	How was the intervention developed?	What does the intervention look like and how is it used?	How is the intervention specifically connected to midwifery?	How does the intervention relate to the ICM midwifery philosophy of care?*	C1	C2	C3
		that women are more willing to accept help from psychiatric services, domestic violence advocacy and other services, where required.<br>Women may be more likely to talk about lifestyle choices, such as smoking and drinking, and to trust the advice they receive [7, 22].<br>However, women may also be unhappy with a perceived intrusive surveillance.<br>It is also hypothesised that there is an improved co-	recruitment (antenatal), labour, birth and the postnatal period, in hospital and community settings and in collaboration with specialist obstetric clinic care, when required.	or at home.<br>Midwives will provide continuity of care in a multi-disciplinary network of consultation and referral with other care providers.<br>Some antenatal and/or intrapartum and/or postpartum care will be provided in consultation with medical staff, as appropriate [5].<br>Within this model, midwives work in partnership with the woman.					

# in ref. list	First author, year, country	What is the theoretical background of the intervention?	How was the intervention developed?	What does the intervention look like and how is it used?	How is the intervention specifically connected to midwifery?	How does the intervention relate to the ICM midwifery philosophy of care?*	C1	C2	C3
		ordination and collaboration between midwives working in continuity models of care and the multi-disciplinary network of support, improved access to care, improved care according to guidelines, earlier recognition of problems and improved health behaviours. Thus, the initiation of treatments to prevent or reduce PTB may occur earlier in the intervention group because the midwives have							

# in ref. list	First author, year, country	What is the theoretical background of the intervention?	How was the intervention developed?	What does the intervention look like and how is it used?	How is the intervention specifically connected to midwifery?	How does the intervention relate to the ICM midwifery philosophy of care?*	C1	C2	C3
		greater knowledge, there are lower barriers for women to contact midwives and faster access to obstetric opinion and management.							
45	Firouzan, 2020, Iran	See: Fenwick, 2013	A group of Australian researchers developed a midwife-led psycho-educational approach called birth emotions - looking to improve expectant fear (BELIEF) to target childbirth fear [19]. See: Fenwick, 2013.	BELIEF is a telephone counseling psycho-educational approach that is offered by midwives. The first author gave two face-to-face counseling sessions in the 24th and 34th weeks of pregnancy to the participants in intervention group (she is a midwife). Between these two	Midwives were involved in developing and providing the intervention.	ICM philosophy: 1-3,5,6,8	+	+	-

# in ref. list	First author, year, country	What is the theoretical background of the intervention?	How was the intervention developed?	What does the intervention look like and how is it used?	How is the intervention specifically connected to midwifery?	How does the intervention relate to the ICM midwifery philosophy of care?*	C1	C2	C3
				sessions, the intervention group received eight telephone-counseling sessions once a week. It emphasizes on expectations and emotions about childbirth fear, expression of feelings, and it helps women to identify and work through the distressing components of childbirth.					
46	Gamble, 2005, Australia	The intervention is within the scope of midwifery practice, caused no harm to participants, was perceived as	The counseling intervention was based on a theoretical perspective focus group discussions with childbearing	Women were recruited during their last trimester of pregnancy, using a variation of questionnaires; EPDS, DASS-21,	The research midwife was integrally involved in the development of the counseling protocol and received regular supervision. The intervention was performed by midwives.	ICM philosophy: 2,3,5-8	+	+	-

# in ref. list	First author, year, country	What is the theoretical background of the intervention?	How was the intervention developed?	What does the intervention look like and how is it used?	How is the intervention specifically connected to midwifery?	How does the intervention relate to the ICM midwifery philosophy of care?*	C1	C2	C3
		helpful, and enhanced women's confidence about a future pregnancy.	women and mid-wives, and reviews of the literature (16,17).	MSSS along with demographic information and reproductive history. Women were inter-viewed within 72 hours of birth to determine if they had experienced a distressing or traumatic birth. Criterion A of DSM-IV-TR for posttraumatic stress disorder was used to screen women. At 4 to 6 weeks and 3 months postpartum, participants complete a questionnaire.					

# in ref. list	First author, year, country	What is the theoretical background of the intervention?	How was the intervention developed?	What does the intervention look like and how is it used?	How is the intervention specifically connected to midwifery?	How does the intervention relate to the ICM midwifery philosophy of care?*	C1	C2	C3
47	Gamble, 2017, Australia	Childbirth fear is relatively common in developed countries. Fearful women are more likely to experience anxiety, depression, and stress as well as isolation and poor social support. Of increasing concern is the association with increased birth intervention, particularly cesarean section and poor maternal emotional well-being postpartum.	A group of Australian researchers developed a midwife led psycho-educational approach called birth emotions - looking to improve expectant fear (BELIEF) to target childbirth fear [19]. See: Fenwick 2013	The intervention for women reporting high childbirth fear included the provision of psychoeducational counseling over the telephone by trained midwives at two time points (Fenwick et al., 2013). Results	Midwives were involved in developing and providing the intervention.	ICM philosophy: 1-3, 5-8	+	+	+
9	George, 2018, Australia	Epidemiology<br>Oral health care during pregnancy is important for the	It is based on epidemiology and knowledge about oral health in pregnancy.	Oral health education.<br>Oral health screening.	Midwives were involved in providing the intervention.	ICM philosophy: 8, little information	-	+	-

# in ref. list	First author, year, country	What is the theoretical background of the intervention?	How was the intervention developed?	What does the intervention look like and how is it used?	How is the intervention specifically connected to midwifery?	How does the intervention relate to the ICM midwifery philosophy of care?*	C1	C2	C3
		health of the mother and child.		Dental referrals for pregnant women at risk of poor oral health.					
48	Gonzales-Plaza, 2021, Spain	ICTs enhance self-control, self-evaluation, self-reinforcement, and personalized feedback through monitoring devices. Thus, ICT interventions based on social cognitive theory could be useful in promoting healthy habits in women who are pregnant. Mobile health allows access to and receipt of health information, which may contribute to the	No information.	The use of a smartband and an app for receiving information and support from a midwife. Women who were pregnant were recommended to take 10,000 steps a day, equivalent to 30 minutes per day of moderate physical activity, over the week (≥5 days) according to the recommendations of the American College of	Midwives gave instructions and support.	ICM philosophy: 3,8	-	+	-

# in ref. list	First author, year, country	What is the theoretical background of the intervention?	How was the intervention developed?	What does the intervention look like and how is it used?	How is the intervention specifically connected to midwifery?	How does the intervention relate to the ICM midwifery philosophy of care?*	C1	C2	C3
		promotion of healthy lifestyles and improvement of maternal and neonatal health.		Obstetricians and Gynecologists. The smartband was linked to the Mi Fit app, which was free and available for Android and iOS systems. The midwife instructed the intervention group's participants on how to set up the step and weight goals through notifications of goals and activated alerts in the Mi Fit app. The smartband would vibrate during prolonged periods of inactivity or send prizes when goals were achieved.					

# in ref. list	First author, year, country	What is the theoretical background of the intervention?	How was the intervention developed?	What does the intervention look like and how is it used?	How is the intervention specifically connected to midwifery?	How does the intervention relate to the ICM midwifery philosophy of care?*	C1	C2	C3
49	Gu, 2021, China	The international midwife-led model of care advocates woman-centred philosophy of care and is based on the premise that pregnancy and childbirth are, in the main, normal life events. The medicalization of childbirth, however, has impacted on the midwifery profession and midwifery models of care globally.	First, a literature was conducted to draft an initial task list of midwifery services for Chinese midwives. Next, a Delphi method to obtain consensus within a panel of experts and developed the final midwifery task list.	Midwives perform task in the care of pregnant and birthing women.	Midwives perform tasks in the care of pregnant and birthing women.<br>Unclear if midwives were involved in the development of the intervention/task list.	ICM philosophy: 1-3,5,8	-	+	-
50	Heins, 1990, USA	Patient education	Does not say – but experience with two South Carolina programs described in previous	One-to-one teaching session where the risk is also estimated at the initial visit.	Women were seen by a nurse-midwife and sometimes by a nurse	ICM philosophy: 4,5,8	-	+/-	-

# in ref. list	First author, year, country	What is the theoretical background of the intervention?	How was the intervention developed?	What does the intervention look like and how is it used?	How is the intervention specifically connected to midwifery?	How does the intervention relate to the ICM midwifery philosophy of care?*	C1	C2	C3
			publications describes the development of the intervention.	Visits at 1-2 weeks interval – women had a special number to phone to if a problem raised.<br>Case load.					
51	Hodnett, et al., 2008, North America en UK	To support normality	Content and positioning, interventions for pain and assessment and interventions for emotional status was adapted from Simkin and Ancheta, 1999.	There were several components of structured care.<br>A minimum of one hour of care provided by a nurse or a midwife trained in structured care, consisting of a formalized approach to assessment of and interventions for maternal emotional state, pain and fetal position.	The care is provided by a nurse or a midwife.<br>Not clear if midwives participated in the development of the intervention.	ICM philosophy: 1,2,5,8	-	+/-	-

# in ref. list	First author, year, country	What is the theoretical background of the intervention?	How was the intervention developed?	What does the intervention look like and how is it used?	How is the intervention specifically connected to midwifery?	How does the intervention relate to the ICM midwifery philosophy of care?*	C1	C2	C3
52	Homer, 2013, Australia	Midwifery continuity of care per se has been widely studied. A systematic review in the Cochrane Library examining midwifery continuity of care included 11 trials (12,276 women). Women with a previous CS are often included in trials of midwifery continuity of care although they have not been specifically studied or targeted.	Not described	Women allocated to the intervention group will receive midwifery continuity of care from a small group of midwives.[27] The midwives provide care during the antenatal, labour and birth, and postnatal periods (to two weeks postnatal). Midwives will adhere to The National Midwifery Guidelines for Consultation & Referral [32]. All women will have an appointment with an obstetric consultant during pregnancy (to re-	Midwives are performing it. Midwives are founders of this type of care and have developed it.	ICM philosophy: 1-3,5-8	+	+	+

# in ref. list	First author, year, country	What is the theoretical background of the intervention?	How was the intervention developed?	What does the intervention look like and how is it used?	How is the intervention specifically connected to midwifery?	How does the intervention relate to the ICM midwifery philosophy of care?*	C1	C2	C3
				assess VBAC suitability and discuss the birth plan). If a woman develops complications during pregnancy and requires additional care, she will continue with the midwifery continuity of care model while also attending obstetric or other consultations (this is the same for both groups). Labour and birth care will be provided at the hospital's birth unit/labour ward and postnatal care will be provided in					

# in ref. list	First author, year, country	What is the theoretical background of the intervention?	How was the intervention developed?	What does the intervention look like and how is it used?	How is the intervention specifically connected to midwifery?	How does the intervention relate to the ICM midwifery philosophy of care?*	C1	C2	C3
				hospital or the woman's own home following discharge from hospital.					
53	Huang, 2023, China	Limited information but emphasized that Internet + continuous midwifery service models has shown to benefit the outcome of pregnancy and birth.	An Internet + continuous midwifery service model based on a traditional group, was completed in three steps: 1.Conduct expert meetings and establish a continuous midwifery service management team. 2. Establish an HRP Maternal Internet Communication Platform. 3. Carrying out Internet and	Used for women in high risk pregnancy. Consisted of Internet + continuous midwifery service mode management	The intervention is developed to enhance midwifery model of care which the authors claim that can no longer meet the needs of the new era.	ICM philosophy: 3,4,6,7,	+	(+)	-

# in ref. list	First author, year, country	What is the theoretical background of the intervention?	How was the intervention developed?	What does the intervention look like and how is it used?	How is the intervention specifically connected to midwifery?	How does the intervention relate to the ICM midwifery philosophy of care?*	C1	C2	C3
			continuous midwifery services.						
54	Hulst, 2004, NL	The most common labor-related technologies used in the birth process can be categorized as follows: (1) midwife technological interventions: sweeping of membranes, amniotomy, and episiotomy; (2) midwife management interventions: consultation with an obstetrician without referral, and referral due to existing or	The intervention is intended homebirth. As such is not described how it was developed but is provided by midwives in Dutch maternity care	In the paper they talk about midwife technological intervention: sweeping of membranes, amniotomy and episiotomy – midwife management interventions: consultations with an obstetrician without referral, and referral due to existing problems	Interventions performed by midwives without involvement of an obstetrician.	ICM philosophy: 1,3,8	-	+	-

# in ref. list	First author, year, country	What is the theoretical background of the intervention?	How was the intervention developed?	What does the intervention look like and how is it used?	How is the intervention specifically connected to midwifery?	How does the intervention relate to the ICM midwifery philosophy of care?*	C1	C2	C3
		anticipated problems for care under supervision of an obstetrician; and (3) obstetrician interventions: induction of labor, augmentation during labor, pharmaceutical pain relief, instrumental delivery (forceps/vacuum extraction), and cesarean section.							
55	Jimenez, 2023, Spain	Study protocol The result of early screening for anxiety and depression in pregnancy suggests the need for more effective health care pathways,	Current literature recommends the use of m-health interventions for the treatment of mood disorders. Also, National Institute of Clinical Excellence (NICE)	There will be a two-arm prospective, randomized, parallel controlled clinical trial. 70 women will be recruited and screened during their pregnancy.	It is aimed at midwives to use in clinical practice.	ICM philosophy: 2,3,4,5,7	-	+	-

# in ref. list	First author, year, country	What is the theoretical background of the intervention?	How was the intervention developed?	What does the intervention look like and how is it used?	How is the intervention specifically connected to midwifery?	How does the intervention relate to the ICM midwifery philosophy of care?*	C1	C2	C3
		including some early interventions that reduce the overall burden of the childbearing situation, both on the mother herself, and her child consequently, and on the health care system.	guidelines describe two levels of intervention for women with mental health problems which the authors use to underpin their protocol						
56	Khademioore, 2023, Iran	Available evidence suggests that the quality improvement of obstetric and newborn care, especially management practices during birth, could prevent a proportion of these deaths. The mobile	To accomplish this, PRONTO International (PRONTO) partnered with CARE India to integrate simulation, team training and post event debriefing after live births into the nurse-	Knowledge and skill indicators from post event debriefs of live births were collected using a mobile Application (App) as part of the nurse-mentoring programme. The mobile App was designed as a job aid to lead guided	It was aimed at midwives knowledge and skills to improve care.	ICM philosophy: 3,8	-	+	-

# in ref. list	First author, year, country	What is the theoretical background of the intervention?	How was the intervention developed?	What does the intervention look like and how is it used?	How is the intervention specifically connected to midwifery?	How does the intervention relate to the ICM midwifery philosophy of care?*	C1	C2	C3
		nurse-mentoring programme aimed to improve auxiliary nurse midwife (ANM) and general nurse midwife (GNM) clinical skill and management practices during birth.	mentoring programme.	debriefs with clinical site staff based on observations recorded during live births					
57	Khan, 2023, global<br><br>SR								
58	Kwegyir-Afful, 2018, Finland	Health education and advice-oriented interventions	Based on clinical guidelines for occupational lifting in pregnancy by MacDonald et al.	Through this study, a model for intervention to reduce physical exertion during pregnancy will be developed and its impact on reducing PTB and LBW will be ascertained.	Midwives will deliver health education based on the clinical guidelines for occupational lifting in pregnancy and research midwives will make 2 home visits (at the beginning and end of the study) with participants' consent.	ICM philosophy: 3,7	-	+	-

# in ref. list	First author, year, country	What is the theoretical background of the intervention?	How was the intervention developed?	What does the intervention look like and how is it used?	How is the intervention specifically connected to midwifery?	How does the intervention relate to the ICM midwifery philosophy of care?*	C1	C2	C3
59	Lugina, 2001, Sweden	The study is seen as a first step in guiding nursing and midwifery interventions for women who express post-partum concerns.	No information		Midwives want more knowledge about post-partum concerns to be able to develop midwifery interventions. The findings can direct nurses and midwives how to develop content and timing of such intervention.	ICM philosophy: 1,2,6,8	-	+/-	-
60	Lundgren, 2020, Sweden/Iceland	Theoretical models for midwifery care have been developed for education and practice in different countries and maternity care settings. These models have similarities by conceptualizing maternity care; by involving the women in the care (woman-	A theoretical midwifery model of woman-centred care (MiMo) has been developed in a Nordic context. The MiMo was developed based on qualitative studies on women's and midwives' experiences of childbirth, and validation through focus group	The intervention was comprised of:<br>1. One-day (8 h) education day for midwives, who should work with the model in practice.<br>2. One-hour meeting to introduce the model to each group of obstetricians, assistant nurses and managers.	The intervention is based on theoretical models of midwifery care, developed and provided by midwives.	ICM philosophy: 1-8	+	+	+

# in ref. list	First author, year, country	What is the theoretical background of the intervention?	How was the intervention developed?	What does the intervention look like and how is it used?	How is the intervention specifically connected to midwifery?	How does the intervention relate to the ICM midwifery philosophy of care?*	C1	C2	C3
		centredness), focusing on the aspects of the midwife-woman relationships, and how to support normal birth.	interviews in Sweden and Iceland. The concept midwifery as used in the MiMo is related to midwives' praxis on labour wards. The concept woman-centred means that the MiMo is based on studies focusing on the labouring and birthing women's needs and perspectives.	3. Regular reflection group meetings with midwives throughout the study period i.e. six occasions/midwife x 1.5 h.					
61	Maga, 2023, global/Italy<br><br>SR								
62	Mannocci, 2022, Italy	The theoretical background lies in a holistic view of maternal health in general post-partum. As such	HAPPY MAMA intervention which focuses on the mother-child dyad and aims to teach both the basic	The intervention aims to improve maternal self-efficacy and mood control. It focuses	It is provided by midwives but also by others who care for women post-partum, such as nurses, job-infant care workers and students in obstetrics.	ICM philosophy: 2,6,8	-	+/-	-

# in ref. list	First author, year, country	What is the theoretical background of the intervention?	How was the intervention developed?	What does the intervention look like and how is it used?	How is the intervention specifically connected to midwifery?	How does the intervention relate to the ICM midwifery philosophy of care?*	C1	C2	C3
		the post-partum period can be supported by health interventions that give support and information. Women with higher level of maternal self-efficacy experience lower levels of stress. (Bandura)	elements for effective childcare and behavioral strategies to cope with the difficulties that occur during this period. The development of the intervention is not explained further but the paper explains what it includes.	on problem solving training.					
63	Maslin, 2004, global	Editorial – arguments for the importance of nursing and midwifery care	No information	No information	Midwives but also nurses provide the intervention	ICM philosophy: 2,8	-	+/-	-
64	McGiveron, 2015, UK	Weight management intervention in pregnancy for obese women reduced the risk of adverse outcomes.	The Bumps and Beyond intervention were designed by the Healthy Lifestyle Midwife lead for Lincolnshire	Either a midwife or healthy lifestyle advisors at hospital antenatal clinics or local community 'health shops' delivered the	The Healthy Lifestyle Midwife lead developed the intervention.	ICM philosophy: 2 en 5	+	+/-	-

# in ref. list	First author, year, country	What is the theoretical background of the intervention?	How was the intervention developed?	What does the intervention look like and how is it used?	How is the intervention specifically connected to midwifery?	How does the intervention relate to the ICM midwifery philosophy of care?*	C1	C2	C3
			Community Health Services in 2009–10 and Lifestyle midwife lead for Lincolnshire United National Health Service (NHS) Trust in 2008–9.	intervention on a one-to-one basis. The full intervention comprised eight sessions, beginning when women were 16 weeks pregnant and continuing every 2–4 weeks until week 36 of pregnancy. Women were weighed at each session and encouraged to attend all of the sessions. The final session (session 8) was delivered postnatal, approximately 6 weeks after the women had given birth.					

# in ref. list	First author, year, country	What is the theoretical background of the intervention?	How was the intervention developed?	What does the intervention look like and how is it used?	How is the intervention specifically connected to midwifery?	How does the intervention relate to the ICM midwifery philosophy of care?*	C1	C2	C3
65	McNeill, 2012, economically developed countries<br><br>SR of SRs								
66	Meedya, et al. 2010, global<br><br>SR								
67	Meedya, et al. 2014, Australia	The theoretical background is informed by theory in midwifery and psychology. Birth territory, Midwifery Guardianship and Self-efficacy.	Based on data from a review about factors that influence prolonged breastfeeding.	It included three antenatal breastfeeding classes and take home learning activities followed by two postnatal lactation consultation phone calls.	Based on a theory in Midwifery.	ICM philosophy: 1,2,5,6,8	+	+	-
68	Morlans, 2022, Spain	Best practice recommended that all pregnant women should have an antenatal and PPD assessment. There is a need in clinical	Unclear	The main intervention was the establishment of a midwifery consultation including four visits in the antenatal period and one in	Midwife was the lead in the implementation project and the performer of the consultation.	ICM philosophy: 8.	+*	+	-

# in ref. list	First author, year, country	What is the theoretical background of the intervention?	How was the intervention developed?	What does the intervention look like and how is it used?	How is the intervention specifically connected to midwifery?	How does the intervention relate to the ICM midwifery philosophy of care?*	C1	C2	C3
		practice to improve care in the field of perinatal mental health, emphasizing the importance of continuity of care with a multidisciplinary approach.		the postpartum period.					
69	Morrell, 2016, global<br><br>SR								
70	Nkowane, 2016, global	Universal health coverage can help to ensure the availability of a sufficient, well-educated and motivated nursing and midwifery workforce to provide the required health services. The	Governments and relevant stakeholders should ensure that the nursing and midwifery workforce is appropriately prepared and enabled to practice to their full scope. Nursing and	Theme 1. Ensuring an educated, competent and motivated nursing and midwifery workforce within effective and responsive health systems at all levels and in different settings.	Interventions that are aiming at strengthening the midwifery workforce at every level (macro, meso, micro).	ICM philosophy: 3-8	-	+/-	-

# in ref. list	First author, year, country	What is the theoretical background of the intervention?	How was the intervention developed?	What does the intervention look like and how is it used?	How is the intervention specifically connected to midwifery?	How does the intervention relate to the ICM midwifery philosophy of care?*	C1	C2	C3
		universal health coverage approach aims to promote strong, efficient, well-run health systems through the promotion of people-centred care, while applying a broad range of interventions related to health promotion, disease prevention, rehabilitation and palliative care (16). This implies provision of a continuum of health interventions throughout the life course. Therefore, the agenda of	midwifery education and practice are taking place in an era of progressive technological advancement, and its promotion is an important element for the future. Technology advances can support transformational outcomes of safe, integrated, high-quality, knowledge-driven, evidenced-based care and educational approaches.	Theme 2. Optimizing policy development, effective leadership, management and governance. Theme 3. Working together to maximize the capacities and potentials of nurses and midwives through intra- and interprofessional collaborative partnerships, education and continuing professional development. Theme 4. Mobilizing political will to invest in building effective					

# in ref. list	First author, year, country	What is the theoretical background of the intervention?	How was the intervention developed?	What does the intervention look like and how is it used?	How is the intervention specifically connected to midwifery?	How does the intervention relate to the ICM midwifery philosophy of care?*	C1	C2	C3
		universal health coverage places the nursing and midwifery workforce at the core of the health response. It is therefore critical to invest in all areas of nursing and midwifery workforce development.		evidence-based nursing and midwifery workforce development.					
71	Ogrodnicz, 2003, global<br><br>SR								
72	Panda, 2014, Ireland	Not discussed.	Does not say	Interventions carried out for women presenting to the antenatal ward, such as vaginal examinations, administration of analgesia, reasons	Performed by midwives.	ICM philosophy: not.	-	+	-

# in ref. list	First author, year, country	What is the theoretical background of the intervention?	How was the intervention developed?	What does the intervention look like and how is it used?	How is the intervention specifically connected to midwifery?	How does the intervention relate to the ICM midwifery philosophy of care?*	C1	C2	C3
				of transferring women to labour ward, mode of birth and duration of labour are discussed.					
73	Perez-Martinez, 2019, Spain	Care throughout pregnancy, birth and low risk postpartum, should be aware of the possible advantages that care by midwives may entail for the mother and infant (Project funded by the Spanish Ministry of Health and Social Policies via the Institute of Health Management (INGESA) in the year 2009 [9982	In 2009, The Fuenlabada University Hospital in Madrid (Spain) implemented a new comprehensive project, which was developed by a multidisciplinary group (obstetricians, midwives and nurses) so that the midwives could care for the low risk postpartum women and issue the clinical report	The midwife was placed in charge of the clinical care and hospital discharge of low risk postpartum women during the hospital stay after giving birth. The duties of the midwife included the examination of the patient to ensure the correct puerperal involution and to discard signs of alarm, which could require medical	Midwives were part of the development and performed the intervention. They brought their knowledge and skill on health education into the intervention.	ICM philosophy: 1,3,5-8.	+	+	-

# in ref. list	First author, year, country	What is the theoretical background of the intervention?	How was the intervention developed?	What does the intervention look like and how is it used?	How is the intervention specifically connected to midwifery?	How does the intervention relate to the ICM midwifery philosophy of care?*	C1	C2	C3
		Royal Decree 924/2009, BOE of May 29]). In an attempt to increase patient satisfaction by maintaining optimal perinatal results.	for hospital discharge.	consultation, as well as a performing a comprehensive assessment of the patient and her environment, which comprised physiological aspects, psychological aspects and social circumstances.					
74	Petersen, 2011, Germany	Not described	Not described	Interventions are part of routine care as provided by a midwife.	Midwives and what they do are the interventions	ICM philosophy: not clear	-	+	-
75	Polanska, 2004, Poland	No theory described on why to use midwives.	Not described, unclear if midwives were involved.	It contains 4 home visits by a midwife who offers information and motivation.	Not described	ICM philosophy: not clear	-	+	-
76	Ray, 2004, Africa, Asia and Latin America								

# in ref. list	First author, year, country	What is the theoretical background of the intervention?	How was the intervention developed?	What does the intervention look like and how is it used?	How is the intervention specifically connected to midwifery?	How does the intervention relate to the ICM midwifery philosophy of care?*	C1	C2	C3
	SR								
77	Rodríguez-Gallego, 2024, Spain	There is a complex physiological relationship between breastfeeding and PPD. Group interventions during the postpartum period, during which women share a safe space of mutual acceptance and understanding, have proven effective in improving depressive symptoms and empowering women to cope with their situation.	Not described	A multicentric cluster randomised controlled trial with a control group (CG) and an intervention group (IG) and was not blinded.	Not described if developed by midwives. It was a midwife-led breastfeeding support group intervention where midwives provided the care and intervention	ICM philosophy: 3,4,5,6,7	-	+	-

# in ref. list	First author, year, country	What is the theoretical background of the intervention?	How was the intervention developed?	What does the intervention look like and how is it used?	How is the intervention specifically connected to midwifery?	How does the intervention relate to the ICM midwifery philosophy of care?*	C1	C2	C3
		Bandura´s theory.							
78	Sigurðardóttir, 2023, Iceland	Women´s birth experiences are known to have profound long-term impact on their well-being and family relationships. Interventions to assist women to process a negative birth experience have shown inconsistent effects. Some studies have shown statistical improvements on women ´s wellbeing where a mixture of counselling, debriefing and	A feasibility study, based on the revised Medical Research Council (MRC) framework for complex interventions studies was used to develop and evaluate the feasibility of the combined intervention. Midwives were involved in this.	The intervention consists of two components based on results from prior studies of writing (Di Blasio et al., 2015) and one face-to-face counselling interview (Gamble et al., 2005) with a midwife women know from antenatal care (Fenwick et al., 2013). The frame for the writing session was adapted from Di Blasio et al. (2015), where women were encouraged to write for 15–20	Midwives were involved in the development and provided the intervention.	ICM philosophy: 2, 3, 5-8.	+	+	-

# in ref. list	First author, year, country	What is the theoretical background of the intervention?	How was the intervention developed?	What does the intervention look like and how is it used?	How is the intervention specifically connected to midwifery?	How does the intervention relate to the ICM midwifery philosophy of care?*	C1	C2	C3
		education was provided and more recently studies involving writing about thoughts, expectations and deep emotions related to birth showed similar effects and were perceived positively by the women.		min about thoughts, expectations and deep emotions related to birth. It was optional to show the midwife the text when they attended the interview. The counselling framework from Gamble et al. (2005) was translated and used with minor adaptions based on the authors ´prior research findings in order to train the midwives and for them to use during the counselling interviews with women					

# in ref. list	First author, year, country	What is the theoretical background of the intervention?	How was the intervention developed?	What does the intervention look like and how is it used?	How is the intervention specifically connected to midwifery?	How does the intervention relate to the ICM midwifery philosophy of care?*	C1	C2	C3
				(Sigurðardóttir et al., 2019). The framework involved components of active listening (Rogers and Farson, 1957) and cognitive behavioural approaches (Beck, 1979) to facilitate shared understanding and awareness of the connections between events, thoughts and emotional challenges. The midwives were encouraged to tailor their approach to individual woman ´s needs while					

# in ref. list	First author, year, country	What is the theoretical background of the intervention?	How was the intervention developed?	What does the intervention look like and how is it used?	How is the intervention specifically connected to midwifery?	How does the intervention relate to the ICM midwifery philosophy of care?*	C1	C2	C3
				keeping the fidelity to the components of the framework.					
79	Simpson, 2016, global<br><br>SR								
80	Smoke, 1988, USA	The potential for specialized prenatal intervention to reduce rates of maternal and infant problems has been evaluated by various programs involving nurse-midwives.	Not described	The staff provided prenatal and postpartum care and education for adolescents. At each visit, the patients were interviewed by the registered nurse and assessed by the nurse-midwife. At most visits the nutritionist provided counseling that placed special emphasis on adequate nutrition	The professional staff of AOS was coordinated by a certified nurse-midwife, who also was involved in the development of the intervention	ICM philosophy: 2,3-6,8	+	+/-	-

# in ref. list	First author, year, country	What is the theoretical background of the intervention?	How was the intervention developed?	What does the intervention look like and how is it used?	How is the intervention specifically connected to midwifery?	How does the intervention relate to the ICM midwifery philosophy of care?*	C1	C2	C3
				for pregnant adolescents. The social worker interviewed each patient at the first visit to AOS, then provided follow-up counseling as indicated. The adolescents saw a minimum of three team members per visit. In addition, the adolescents attended an educational program of nine one-hour classes given by the professional staff. The classes included additional basic prenatal information, which					

# in ref. list	First author, year, country	What is the theoretical background of the intervention?	How was the intervention developed?	What does the intervention look like and how is it used?	How is the intervention specifically connected to midwifery?	How does the intervention relate to the ICM midwifery philosophy of care?*	C1	C2	C3
				was given at an instructional level of their understanding and emphasized the problem areas of adolescent pregnancy.<br>In a mix of formal and informal atmosphere the classes included the giving of information and use of group interaction, questioning techniques, examples, audiovisual aids, and feedback. The majority of the classes were taught by the nurse and nutritionist with					

# in ref. list	First author, year, country	What is the theoretical background of the intervention?	How was the intervention developed?	What does the intervention look like and how is it used?	How is the intervention specifically connected to midwifery?	How does the intervention relate to the ICM midwifery philosophy of care?*	C1	C2	C3
				assistance from the nurse-midwife.					
81	Souto, et al., 2020, global<br><br>Protocol for SR								
82	Souto, 2021, global<br><br>erratum	In the title, abstract, and body of the manuscript, the term ‘‘midwifery intervention(s)’’ should read ‘‘midwife intervention(s).’’ The term ‘‘midwife intervention’’ indicates that the intervention is conducted specifically by a health professional with the title of ‘‘midwife,’’ which is the population and concept of interest.							

# in ref. list	First author, year, country	What is the theoretical background of the intervention?	How was the intervention developed?	What does the intervention look like and how is it used?	How is the intervention specifically connected to midwifery?	How does the intervention relate to the ICM midwifery philosophy of care?*	C1	C2	C3
		An error was made in an earlier publication.							
83	Souto, 2022, global<br><br>SR								
84	Spindler, 2017, India	Postevent debriefing, is understood to be an effective aspect of clinical education, quality improvement and systems learning (Agency for Healthcare Research and Quality, 2016) as it provides a space for self-reflection by providers including their role and behaviour, knowledge and skills, and team	PRONTO International (PRONTO) partnered with CARE India to integrate simulation, team training and postevent debriefing after live births into the nurse-mentoring programme.	The mobile App was designed as a job aid to lead guided debriefs with clinical site staff based on observations recorded during live births.	The App was used by nurse-midwives.	ICM philosophy: 3,8.	-	+	-

# in ref. list	First author, year, country	What is the theoretical background of the intervention?	How was the intervention developed?	What does the intervention look like and how is it used?	How is the intervention specifically connected to midwifery?	How does the intervention relate to the ICM midwifery philosophy of care?*	C1	C2	C3
		operation. In this intervention, postevent debriefing of live births was defined as a "structured and guided reflection process where students actively appraised their cognitive, affective and psychomotor performance within the context of their clinical judgment skill" (Al Sabei & Lasater, 2016).							
85	Swann, & Davis, 2012, UK	Not described	Not described	Interventions performed by midwives as alternatives to more medicalized interventions.	Performed by midwives and part of the midwifery philosophy of care as focusing on improving normality.	ICM philosophy: 1-3, 5-8.	-	+	-
86	Taylor Miller, 2021, global								

# in ref. list	First author, year, country	What is the theoretical background of the intervention?	How was the intervention developed?	What does the intervention look like and how is it used?	How is the intervention specifically connected to midwifery?	How does the intervention relate to the ICM midwifery philosophy of care?*	C1	C2	C3
	SR								
87	Toohill, 2017, Australia	See Fenwick, 2013.				ICM philosophy: 1-3,5-8	+	+	+
88	Toohill, 2019, Australia	See Fenwick, 2013.				ICM philosophy: 1-3,5-8	+	+	+
89	Truva, 2021, Greece	It is well established that breastfeeding has benefits for infants, children, mothers, and public health [1–3]. Therefore, exclusive breastfeeding remains a standard recommendation by the majority of women's and children's health organizations, including the World Health	It consisted of prenatal and postnatal breastfeeding training and was based on the WHO's guidelines on breastfeeding, the 10 steps of successful breastfeeding in infant-friendly hospitals, the international code of marketing of breast milk substitutes, and the	The midwifery intervention, which included prenatal and postnatal education as well as support for breastfeeding, was carried out individually and took place in two phases.<br>First, women in the midwifery intervention subgroup were asked to attend an individual breast-	Midwives were the main professionals in the conduct of the intervention and were seen as being in a position to influence and increase breastfeeding rates.	ICM philosophy: 1-3,5,6,8	-	+	+

# in ref. list	First author, year, country	What is the theoretical background of the intervention?	How was the intervention developed?	What does the intervention look like and how is it used?	How is the intervention specifically connected to midwifery?	How does the intervention relate to the ICM midwifery philosophy of care?*	C1	C2	C3
		Organization (WHO) [10,11]. Personalized education and support are likely to increase breastfeeding initiation, exclusivity and duration rates [37–39].	scientific guidelines of the Institute of Child Health Greece [54]. No further details of the development process are described.	feeding counselling session that lasted for three hours. In a specific area of the hospital, midwifery intervention subgroups underwent individual training for breastfeeding by a specialized midwife. The midwifery intervention took place after the 34th week of gestation and was free of charge.<br>The women's partners were not present during the time of the intervention.<br>The women of the two midwifery					

# in ref. list	First author, year, country	What is the theoretical background of the intervention?	How was the intervention developed?	What does the intervention look like and how is it used?	How is the intervention specifically connected to midwifery?	How does the intervention relate to the ICM midwifery philosophy of care?*	C1	C2	C3
				intervention subgroups were further informed about the anatomy and physiology of milk production, the importance of exclusive breastfeeding, the breastfeeding on-demand and the importance of the skin-to-skin contact. Participants were asked to share their concerns with the midwife and their personal perceptions of breastfeeding were discussed in detail (Table 1). In terms of increased					

# in ref. list	First author, year, country	What is the theoretical background of the intervention?	How was the intervention developed?	What does the intervention look like and how is it used?	How is the intervention specifically connected to midwifery?	How does the intervention relate to the ICM midwifery philosophy of care?*	C1	C2	C3
				awareness, we used leaflets of the Institute of Child Health-Greece, medical animation dolls, and presentations. An illustrated breastfeeding information pamphlet (of the Institute of Child Health—Alcyone) was given to each pregnant woman after the end of the intervention, as well as the telephone number of the midwife in charge to answer any possible questions and problems related to breastfeeding. The					

# in ref. list	First author, year, country	What is the theoretical background of the intervention?	How was the intervention developed?	What does the intervention look like and how is it used?	How is the intervention specifically connected to midwifery?	How does the intervention relate to the ICM midwifery philosophy of care?*	C1	C2	C3
				second phase of the midwifery intervention consisted of a planned visit on the second to third day after childbirth during their stay in the postnatal ward, focusing on reviewing the process of breastfeeding, the attachment of the newborn, the enhancement of the mother's self-efficacy, the recognition of signs of nutritional adequacy of the new-born, the resolution of breastfeeding problems and					

# in ref. list	First author, year, country	What is the theoretical background of the intervention?	How was the intervention developed?	What does the intervention look like and how is it used?	How is the intervention specifically connected to midwifery?	How does the intervention relate to the ICM midwifery philosophy of care?*	C1	C2	C3
				direct support during breastfeeding observations. We observed the position and attitude of the mother while holding the baby, the method of supporting the breast, the infant's latching on to breast when suckling, and signs of effective suckling and gave specific instructions and advice (see Table 1 and for more details Table S1).					
90	Türkmen, 2021, Turkey	The concept of comfort is categorized in three levels: ease,	Not described.	Three research groups were formed: massage application group	Provision of comfort during labor is among midwifery responsibilities. Provided by midwives.	ICM philosophy: 1,3,8	+	+	-

# in ref. list	First author, year, country	What is the theoretical background of the intervention?	How was the intervention developed?	What does the intervention look like and how is it used?	How is the intervention specifically connected to midwifery?	How does the intervention relate to the ICM midwifery philosophy of care?*	C1	C2	C3
		relief, and transcendence. If labor pain is managed successfully, pregnant women feel relieved. Pregnant women who can cope with labor pain and do not fear childbirth show transcendence. Provision of comfort during labor is among midwifery responsibilities. Midwives are responsible for the planning and implementation of nonpharmacologic methods to reduce labor pain in		(MG), heat application group (HAG), and a control group (CG) (n=30 for each group). Sacral massage or sacral heat were applied to the intervention groups. Only standard midwifery care was given to the control group (Fig.1). Massage was applied for 10 min. (during 4-5 cm, 6-7cm and 8-9 cm cervical dilation of labor). Heat application was applied for 20 min. (during 4-5 cm, 6-7 cm and 8-9 cm	Midwives were also the research team.				

# in ref. list	First author, year, country	What is the theoretical background of the intervention?	How was the intervention developed?	What does the intervention look like and how is it used?	How is the intervention specifically connected to midwifery?	How does the intervention relate to the ICM midwifery philosophy of care?*	C1	C2	C3
		pregnant women and increase labor comfort levels.		cervical dilation of labor).<br><br>*Routine oxytocin infusion is applied to pregnant women in the hospital where this study was conducted. To all pregnant women in each group, 10 IU/ml oxytocin infusion was applied with 4 drops/min in 4-5cm cervical dilation. Oxytocin infusion was increased by 4 drops every 20min and a maximum of 40 drops/min were applied.*					
91	Türkmen, 2024, Turkey	Managing labor pain, increasing comfort, and reducing anxiety are among the basic elements of	Not described.	Ice massage was performed when the participant was at 4 to 5 cm, 6 to 7 cm, and 8 to 9 cm dilatation during 3	Provision of comfort during labor is among midwifery responsibilities. Provided by midwives. Midwives were also the research team.	ICM philosophy: 1,3,8	+	+	-

# in ref. list	First author, year, country	What is the theoretical background of the intervention?	How was the intervention developed?	What does the intervention look like and how is it used?	How is the intervention specifically connected to midwifery?	How does the intervention relate to the ICM midwifery philosophy of care?*	C1	C2	C3
		midwifery care. Pharmacologic methods that reduce labor pain have various side effects on fetal and maternal health. Therefore, non-pharmacologic methods, which usually have no side effects for the fetus and the woman and provide comfort, should be offered among the different options for pain management.		contractions. After the participant chose a comfortable position, the midwife identified the SP6 point on both legs and performed ice massage using rotational movements. The SP6 point is located 3 to 4 fingerbreadths above the posterior malleolus bone, in the ankle (Figure 2). To prevent the direct contact of ice with the skin, the ice was wrapped in gauze.					

# in ref. list	First author, year, country	What is the theoretical background of the intervention?	How was the intervention developed?	What does the intervention look like and how is it used?	How is the intervention specifically connected to midwifery?	How does the intervention relate to the ICM midwifery philosophy of care?*	C1	C2	C3
92	Turkstra, 2017, Australia	Described in Toohill 2014: A midwifery counseling framework for distressed postpartum women developed by Gamble and Creedy.	Not described	Women in the intervention group received psycho-education sessions at 24 and 34 weeks by telephone at a scheduled time convenient to them. Psycho-education sessions were around one hour duration (first session range: 22–125 minutes; second session range: 10–104 minutes) [2].	Based on a midwifery counseling framework Developed and performed by midwives	ICM philosophy: 2,3,8	+	+	-
93	Wallace, 2006, UK	Several authors advocate that midwives should provide verbal or ‘hands off’ care to achieve effective positioning of the mother and baby		The experimental group of midwives attended a 4-hr long workshop covering the rationale and skills of a ‘hands off’ approach to care at	Midwives were part of the research team.The trial protocol required that this care was delivered by a midwife, which was not required by local maternity unit policies at this time.	ICM philosophy: 1,3,6,8	+	+	-

# in ref. list	First author, year, country	What is the theoretical background of the intervention?	How was the intervention developed?	What does the intervention look like and how is it used?	How is the intervention specifically connected to midwifery?	How does the intervention relate to the ICM midwifery philosophy of care?*	C1	C2	C3
		and observation of cues that the baby is correctly attached. This is because mothers need to be able to attach their babies to their breasts themselves, and these elements achieve pain free and effective milk transfer.		first feed, including explanation of the protocol (Inch et al., 2003a,b) and measures described below. The experimental protocol included advice about baby initiation of feeding, positioning and attachment. The rationale included physiological explanation of milk synthesis, supply and removal, facilitated by correct attachment of the baby to the breast, rather than the nipple. Positioning of the mother and baby to					

# in ref. list	First author, year, country	What is the theoretical background of the intervention?	How was the intervention developed?	What does the intervention look like and how is it used?	How is the intervention specifically connected to midwifery?	How does the intervention relate to the ICM midwifery philosophy of care?*	C1	C2	C3
				achieve comfortable and effective feeding includes ensuring the mother is sitting upright and supported, her baby is supported and able to take sufficient breast tissue into the mouth, feeding is uninterrupted and feed times and duration are baby led. Verbal-only care was advised to ensure the mother was able to attach the baby herself. A leaflet explained this information and also reminded mothers that their baby needed only					

# in ref. list	First author, year, country	What is the theoretical background of the intervention?	How was the intervention developed?	What does the intervention look like and how is it used?	How is the intervention specifically connected to midwifery?	How does the intervention relate to the ICM midwifery philosophy of care?*	C1	C2	C3
				breast milk until at least 4 months post-partum.					
94	Wang, 2021, global<br><br>SR								
95	Wang, 2023, China	We drafted the weight management program based on 8 relevant clinical guidelines and 2 expert consensuses.	15 experts in the field of obstetrics, maternal and child health, midwifery, evidence-based healthcare were consulted to evaluate and finalize the draft.	Pregnant women in the intervention group participated in the midwife-led weight management program. They were required to attend the midwife's antenatal clinic for 5 contacts as follows.<br>During each visit, the midwife would provide individualized guidance for the woman according to her	Midwives were partly involved in the development and provided the interventions.	ICM philosophy: 3,8.	+	+	-

# in ref. list	First author, year, country	What is the theoretical background of the intervention?	How was the intervention developed?	What does the intervention look like and how is it used?	How is the intervention specifically connected to midwifery?	How does the intervention relate to the ICM midwifery philosophy of care?*	C1	C2	C3
				pre-pregnancy body mass index range and engage her in shared decision making on gestational weight management.					
96	Warren, 2017, UK	This brief midwife led intervention was based upon the Self Determination Theory (SDT) framework and utilised Motivational Interviewing and individualised goal setting. Pivotal to the theory is the notion that people have a natural propensity towards personality growth and development.	Interventions designed to modify lifestyle behaviours should have, and make explicit reference to, theoretical underpinning, as interventions based on theory have been found to be more effective.	1. A brief counselling session incorporating Motivational Interviewing and individual goal setting lasting between 10–15 minutes (at approx. 16 weeks gestation)<br>2. A personalised magnetic goal card sent to participants within a week<br>3. A follow-up telephone call lasting 5 minutes	The intervention was developed by midwives and given to women in midwife-led care.	ICM philosophy: 3,5-8.	+	+	-

# in ref. list	First author, year, country	What is the theoretical background of the intervention?	How was the intervention developed?	What does the intervention look like and how is it used?	How is the intervention specifically connected to midwifery?	How does the intervention relate to the ICM midwifery philosophy of care?*	C1	C2	C3
				(two weeks after initial session).					
97	Wei, 2021, China	Parturients, especially those with scarred uterus, are anxious and scared of spontaneous vaginal delivery, and thus the implementation of active nursing intervention is very important. Midwife intervention nursing mode as an obstetric nursing mode dominated by midwives is more humanized and targeted versus routine obstetric care. Midwives by virtue of professional	Not described	Midwife intervention: (1) Outpatient service: one-to-one midwife outpatient consultation services were provided for parturients at first, second and third trimester. Services mainly included: introducing pregnant women, parturients and their families to the safety and feasibility of spontaneous vaginal delivery in the scarred uterus and answering all questions and	See theoretical background	ICM philosophy: 1,3,5-8.	-	+	-

# in ref. list	First author, year, country	What is the theoretical background of the intervention?	How was the intervention developed?	What does the intervention look like and how is it used?	How is the intervention specifically connected to midwifery?	How does the intervention relate to the ICM midwifery philosophy of care?*	C1	C2	C3
		technical level provide more scientific, comprehensive and careful nursing services for parturients, ensuring smooth delivery to the maximum extent [4]. A study has confirmed that midwife intervention nursing mode can decrease the risk of adverse maternal and infant outcomes and improve the degree of anxiety and depression of parturients, and as a result more parturients agree		doubts of pregnant and parturient women so as to improve their confidence to select spontaneous vaginal delivery; daily popularizing the significance of self-management in prenatal weight, diet and others; strengthening pregnant and parturient women to identify abnormal manifestations and fully know normal pregnant performances so as to perceive abnormal condition timely; introducing pregnant and					

# in ref. list	First author, year, country	What is the theoretical background of the intervention?	How was the intervention developed?	What does the intervention look like and how is it used?	How is the intervention specifically connected to midwifery?	How does the intervention relate to the ICM midwifery philosophy of care?*	C1	C2	C3
		to trial-produce vaginally, significantly increasing the success rate of spontaneous vaginal delivery [5].		parturient women to the way to labor during parturition and auxiliary labor methods like Lamaze breathing method and anodyne labor; psychological counseling to establish the confidence of pregnant and parturient women in spontaneous vaginal delivery, give faith support and release negative emotions; providing corresponding assessment based on the individual condition of pregnant and					

# in ref. list	First author, year, country	What is the theoretical background of the intervention?	How was the intervention developed?	What does the intervention look like and how is it used?	How is the intervention specifically connected to midwifery?	How does the intervention relate to the ICM midwifery philosophy of care?*	C1	C2	C3
				parturient women and health education, and appropriately increasing outpatient consultation services based on the condition of pregnant and parturient women. (2) Intrapartum management: Conditions of parturients for spontaneous vaginal delivery were assessed sufficiently, and the reason of last cesarean section was known, taken seriously and avoided. The principle of “stop”					

# in ref. list	First author, year, country	What is the theoretical background of the intervention?	How was the intervention developed?	What does the intervention look like and how is it used?	How is the intervention specifically connected to midwifery?	How does the intervention relate to the ICM midwifery philosophy of care?*	C1	C2	C3
				was always followed during the labor process. If parturients decided to give up spontaneous vaginal delivery, midwives should fully understand the needs of parturients, immediately reassess the condition for vaginal delivery and provide corresponding intervention, such as psychological counseling and relieving uneasy, anxious and other emotions of parturients [9]. Once fetal distress,					

# in ref. list	First author, year, country	What is the theoretical background of the intervention?	How was the intervention developed?	What does the intervention look like and how is it used?	How is the intervention specifically connected to midwifery?	How does the intervention relate to the ICM midwifery philosophy of care?*	C1	C2	C3
				uterine rupture with aura and other abnormal phenomena were observed, the preparation for emergency cesarean section was completed immediately, including blood matching, informing the operating room, anesthesiology department and neonatology department, etc. Continuous fetal rate monitoring was performed during the labor process to closely monitor maternal and infant					

# in ref. list	First author, year, country	What is the theoretical background of the intervention?	How was the intervention developed?	What does the intervention look like and how is it used?	How is the intervention specifically connected to midwifery?	How does the intervention relate to the ICM midwifery philosophy of care?*	C1	C2	C3
				abnormal condition. Parturients were guided to use free position and birthing ball for relieving pain caused by uterine contraction and were advised to select epidural anesthesia for painless labor. Indications for vaginal midwifery were relaxed properly during the second stage of labor, and abdominal pressure was forbidden. A neonatologist was present before the forthcoming delivery of the baby					

# in ref. list	First author, year, country	What is the theoretical background of the intervention?	How was the intervention developed?	What does the intervention look like and how is it used?	How is the intervention specifically connected to midwifery?	How does the intervention relate to the ICM midwifery philosophy of care?*	C1	C2	C3
				to always adequately prepare for newborn rescue and resuscitation and to give timely intervention. After the delivery of the baby, the delivery of placenta was observed and the completeness of placenta was checked. Moreover, active management and intervention were carried out to prevent postpartum hemorrhage. (3) Postpartum management: Postpartum management was strengthened, and					

# in ref. list	First author, year, country	What is the theoretical background of the intervention?	How was the intervention developed?	What does the intervention look like and how is it used?	How is the intervention specifically connected to midwifery?	How does the intervention relate to the ICM midwifery philosophy of care?*	C1	C2	C3
				basic vital signs (heart rate, pulse, blood pressure, etc.) and vaginal bleeding of parturients were observed closely. Thus, abnormal condition could be disposed in time. The midwife visited parturients on the first day after delivery to assess the condition of uterine involution, puerpera mentality and breast-feeding, and to provide necessary psychological counseling and postpartum rehabilitation training.					

# in ref. list	First author, year, country	What is the theoretical background of the intervention?	How was the intervention developed?	What does the intervention look like and how is it used?	How is the intervention specifically connected to midwifery?	How does the intervention relate to the ICM midwifery philosophy of care?*	C1	C2	C3
98	Wilkinson, 2016, UK	Cognitive Behavioural Therapy (CBT)	The research will adapt and build on existing interventions utilising CBT principles, such as Towards Parenthood. The planned intervention will be developed for optimal efficacy, feasibility and acceptability with a broad population of pregnant women experiencing a range of anxiety symptoms of differing severity, with the aim of reducing the overall population burden of antenatal anxiety.	Embedding the intervention in routine antenatal care as a brief midwife-led group format with 3 sessions additional to the routine check-ups, led by a trained midwife and psychology supporter, with each session lasting approximately 90 minutes. Sessions will be held at 3-week intervals.	Midwives are conducting the intervention.	ICM philosophy: 2,3,5,6,8.	-	+	+

SR = systematic review

* ICM Philosophy of Midwifery Care

1. Pregnancy and childbearing are usually normal physiological processes.
2. Pregnancy and childbearing is a profound experience, which carries significant meaning to the woman, her family, and the community.
3. Midwives are the most appropriate care providers to attend childbearing women.
4. Midwifery care promotes, protects and supports women's human, reproductive and sexual health and rights, and respects ethnic and cultural diversity. It is based on the ethical principles of justice, equity, and respect for human dignity.
5. Midwifery care is holistic and continuous in nature, grounded in an understanding of the social, emotional, cultural, spiritual, psychological and physical experiences of women.
6. Midwifery care is emancipatory as it protects and enhances the health and social status of women and builds women's self-confidence in their ability to cope with childbirth.
7. Midwifery care takes place in partnership with women, recognising the right to self-determination, and is respectful, personalised, continuous and non-authoritarian.
8. Ethical and competent midwifery care is informed and guided by formal and continuous education, scientific research and application of evidence.

Supplement 5: Content orientation of the midwifery Interventions

# in ref. list	First author, year	Content orientation of intervention	Count of separate intervention*	Exact naming of the intervention	Definition
18	Abhari, 2020	Maternal mental health	See 30.	Midwifery-led counseling intervention	A counseling intervention to prevent and mitigate psychological birth trauma.<br>Based on an intervention developed by Gamble et al.
19	Abou Malham, 2015	Professional role	1	Midwifery intervention	An intervention (action plan) aiming to strengthen the midwifery professional role.
20	Adams, 2017	Public health	2	Nurse-midwife-led oral health intervention	An intervention embedded in Centering-Pregnancy to promote oral health.
21	Ajuebor, 2019	Professional role	3	Nursing and midwifery policy intervention<br>Nursing and midwifery intervention	Intervention/ program to address the specific obstacles to optimized contributions of nurses and midwives to universal health coverage and sustainable development goals.
22	Alderdice, 2013	Maternal mental health	SR	A midwifery-led intervention<br>Midwifery intervention	Non-invasive interventions in the perinatal period that enable midwives to offer effective care to women within the area of maternal mental health and well-being.
23	Allen, 2016	Organization of care	See 7.	Complex intervention of caseload midwifery	The intervention provided caseload midwifery.
24	Allen, 2017	Organization of care	4	Intervention of case-load midwifery	Caseload midwifery provides high-level relational continuity whereby childbearing women receive antenatal, intrapartum and postnatal care from a primary midwife and her/his back-up midwives.
25	Altiner, 2019	Professional role	5	Multitasking midwifery/ nurse intervention	Any task done by midwives in the center are regarded as midwifery intervention.
26	Asadzadeh, 2020	Maternal mental health	See 30.	A midwife-led brief counseling intervention	Intervention for postpartum women experiencing PTSD symptoms. The intervention approach was based on<br>Gamble and colleagues' protocol.
27	Bick, 2022	Public health	6	A midwife-led antenatal intervention	An intervention to support women to perform pelvic floor muscle exercises in pregnancy.

# in ref. list	First author, year	Content orientation of intervention	Count of separate intervention*	Exact naming of the intervention	Definition
28	Blomgren, 2023	Professional role	7	A midwife-led quality improvement intervention	The intervention targets to bridge the evidence-to-practice gap within midwifery and effectively apply this across different settings.
29	Borg Cunen, 2014	Maternal mental health	SR	Midwife-led intervention	Interventions that could be implemented by a midwife to support women with post traumatic stress after childbirth.
30	Borges, 2021	Organization of care	See 7.	Caseload midwifery as a legitimate intervention	An intervention by midwives that improves health outcomes in pregnant women with cystic fibrosis.
31	Borneskog, 2023	Professional role	8	Life-saving midwifery intervention	No definition
32	Bryce, 2009	Public health	9	A home-based midwifery intervention<br>A supportive midwifery intervention	A responsive smoking cessation service that would meet the particular needs of pregnant women aged 25 years and under
33	Caelli, 2002	High risk pregnancy	10	Midwife-managed intervention	Intervention additional to routine care to support and educate high-risk pregnant women and their partners subsequent to the death of a baby in a previous pregnancy.
34	Coates, 2019	Maternal mental health	SR	Midwife-led (counseling / perinatal emotional support) intervention	Intervention aiming at women at risk for perinatal mental health issues that is embedded in midwifery care and performed by midwives.
35	Dai, 2024	Public health	11	Midwifery-led mobile health app intervention	The (telemonitoring) intervention to enhance maternal health management by offering pregnant women convenient access to professional midwifery care and encouraging women's self-management throughout pregnancy.
36	Dawson, 1999	High risk pregnancy	12	Midwifery intervention	Domiciliary midwifery support delivered by midwives in high-risk pregnancy incorporating telephonic fetal heart rate monitoring
37	De Wolff, 2019/2021	High risk pregnancy	13	Midwife-coordinated maternity care intervention	An intervention delivered to pregnant women with pre-existing chronic medical conditions by midwives.
10	Endqvist, 2017	Birth	14	A multifaceted midwifery intervention	Intervention to reduce second-degree tears in primiparous women.

# in ref. list	First author, year	Content orientation of intervention	Count of separate intervention*	Exact naming of the intervention	Definition
38	Evans, 2020	Maternal mental health	15	A midwife facilitated intervention	Intervention specifically designed to support pregnant women with mild to moderate anxiety.
39	Evans, 2022	Maternal mental health	See 22	A midwife-led intervention	Intervention specifically designed to support pregnant women with mild to moderate anxiety.
40	Evans, 2022b	Maternal mental health	See 22	A midwife facilitated intervention	Intervention specifically designed to support pregnant women with mild to moderate anxiety.
41	Fenwick, 2011	Maternal mental health	See 30.	A midwife-led counselling intervention	An intervention promoting resilience in mothers emotions' (PRIME), on anxiety responses and depression in childbearing women.
42	Fenwick, 2013	Maternal mental health	See 30.	A midwife-led psycho-education intervention	BELIEF: an intervention for reducing women's fear during pregnancy.
43	Fenwick, 2015	Maternal mental health	See 30.	A midwife psycho-education intervention	BELIEF: an intervention for reducing women's fear during pregnancy.
44	Fernandez, 2019	Organization of care	See 37	Midwifery continuity of care intervention	Continuity of care is defined as delivering care that acknowledges that a woman's health needs are related to events and should be managed over time [6].
45	Firouzan, 2020	Maternal mental health	See 30.	Midwife-led psycho-educational intervention	An intervention delivered by midwives to reduce fear of childbirth.
46	Gamble, 2005	Maternal mental health	16	A midwife-led brief counseling intervention	An intervention where midwives counsel postpartum women at risk of developing psychological trauma symptom.
47	Gamble, 2017	Maternal mental health	See 30.	Midwife psycho-education intervention	An intervention delivered by midwives to reduce fear of childbirth.
9	George, 2018	Public health	17	Midwifery intervention Midwifery initiated oral health-dental service	An intervention initiated by midwives to promote oral hygiene during pregnancy.
48	Gonzalez-Plaza, 2021	Public health	18	Midwife counselling intervention	A complex digital health intervention, using a smartband and app with midwife counseling, on GWG and physical activity (PA) in women who are pregnant and have obesity.

# in ref. list	First author, year	Content orientation of intervention	Count of separate intervention*	Exact naming of the intervention	Definition
49	Gu, 2021	Professional role	19	Midwifery-led task shifting interventions	Midwife-led services for Chinese pregnant women by shifting tasks in maternity care towards midwives.
50	Heins, 1990	High risk pregnancy	20	Nurse-midwifery intervention	Care given by a nurse-midwife (rarely a nurse) to women with a high risk for low birth weight.
51	Hodnett, 2008	Birth	21	Complex nursing and midwifery intervention to support normality in birth	Nursing or midwifery care or a minimum of one hour of care by a nurse or midwife trained in structured care when entering the hospital intrapartum.
52	Homer, 2013	Organization of care	22	The intervention: midwifery continuity of care	Women have a midwife caring for them during labour and birth whom they have met before and feel that they know, and this trusting relationship increases their confidence.
53	Huang, 2023	Organization of care	See 37.	Midwifery service intervention<br>Midwifery intervention	The intervention offers maternal management from prenatal to postpartum, in-hospital to out-of-hospital, and offline to online.
54	Hulst, 2004	Professional role	23	(1) Midwife technological interventions;<br>(2) Midwife management interventions	Explained by examples: (1) midwife technological interventions: sweeping of membranes, amniotomy, and episiotomy; (2) midwife management interventions: consultation with an obstetrician without referral, and referral due to existing or anticipated problems for care under supervision of an obstetrician.
55	Jimenez, 2023	Maternal mental health	24	A midwife-led e-health intervention	An intervention to reduce anxiety during pregnancy.
56	Khademioore, 2023	Maternal mental health	25	Tele-midwifery intervention	The intervention is an interactive mHealth application based on education and continuous support provided by midwives on FOC, childbirth self-efficacy, and birth mode in primiparous women
57	Khan, 2023	Organization of care	SR	Midwifery models of care intervention	Midwifery models of care were defined as interventions with midwives, or those similarly qualified based on the setting, as the central care providers or coordinators of care.
58	Kwegyir, 2018	Public health	26	Midwife-led 3-component liftless intervention	The intervention aims to decrease lifting exposure during pregnancy among Ghanaian women.

# in ref. list	First author, year	Content orientation of intervention	Count of separate intervention*	Exact naming of the intervention	Definition
59	Lugina, 2001	Maternal mental health	27	Nursing /midwifery intervention (to be developed)	First step towards an intervention that decreases worry and increases interest and confidence of mothers postpartum.
60	Lundgren, 2020	Organization of care	28	MiMO (midwifery model of woman-centred care) intervention	A theoretical midwifery model of woman-centred care (MiMo) developed in a Nordic context.
61	Maga, 2023	Professional role	SR	Midwifery intervention	Midwifery interventions are defined as elements of maternity care provided by midwives to improve and optimize the health outcomes of women, newborns, and the public health of society at large.
62	Mannocci, 2022	Maternal mental health	29	Midwifery intervention	Intervention to increase maternal self-efficacy and reduce stress.
63	Maslin, 2004	Professional role	30	Nursing and midwifery interventions	Interventions that demonstrates the contribution of nursing and midwifery to the provision of cost-effective, quality health care.
64	McGiveron, 2015	Public health	31	A midwife-led intervention	An antenatal weight management intervention comprising a one-to-one programme involving pregnant women with specialist midwives or healthy lifestyle advisors.
65	McNeill, 2012	Public health	SR	Midwifery intervention<br>Midwifery public health interventions	Public health interventions during pregnancy and postnatal that can be implemented by midwives. But also by care providers that similar roles.
66	Meedya, 2010	Public health	SR	Midwifery intervention<br>A midwife-provided educational intervention	First step towards an intervention that strengthens modifying factors for breastfeeding duration up to 6 months postpartum.
67	Meedya, 2014	Public health	32	A multiphased midwifery intervention called the Milky Way	An intervention to support women who breastfeeding.
68	Morlans, 2022	Organization of care	See 28.	Midwife-led continuity of care interventions	The main intervention was the establishment of a midwifery consultation including four visits in the antenatal period and one in the postpartum period.

# in ref. list	First author, year	Content orientation of intervention	Count of separate intervention*	Exact naming of the intervention	Definition
69	Morrell, 2016	Maternal mental health	SR	Midwifery-led interventions	Interventions to prevent post-natal depression:<br>Interpersonal psychotherapy (IPT)-based intervention.<br>Parent infant interaction.<br>ITP- based intervention,<br>PCA based and CBT based intervention.
70	Nkowane, 2021	Professional role	Policy paper	Midwifery interventions	Interventions that strengthens the workforce of midwifery or that improves the impact of the work of midwives.
71	Ogrodniczuk, 2003	Maternal mental health	SR	Midwife(-type) intervention	Interventions provided by midwives to prevent postnatal depression.
72	Panda, 2014	Birth	33	Midwifery interventions in early labour	Interventions performed by midwives when women present in the antenatal ward at the start of labour.
73	Perez-Martinez, 2019	Professional role	34	Midwives' intervention	Puerperal health education provided to women in the hospital by midwives individually on a daily basis during the clinical rounds and at discharge.
74	Petersen, 2011	Birth	35	Interventions applying midwifery care techniques	The presence of the midwife attending the woman in labour was considered an interventions as well as using midwifery care techniques such as vertical positioning.
75	Polanska, 2004	Public health	36	The midwife-assisted smoking cessation intervention	An intervention where midwives helped pregnant women to quit smoking.
76	Ray, 2004 global SR	Birth	SR	Midwife and traditional birth attendant-based interventions.	Interventions performed by midwife and traditional birth attendant to improve maternal mortality focus on birth.
77	Rodriguez-Gallego, 2024	Public health	37	Midwife-led breastfeeding support group intervention	Group interventions during the postpartum period to prevent postpartum depression.
78	Sigurðardóttir, 2023	Maternal mental health	38	A postpartum midwifery counselling intervention	Intervention offered by midwives to assist women to process a negative birth experience.
79	Simpson, 2016	Maternal mental health	SR	Midwifery-led intervention	Interventions that offer emotional support from midwives during the antenatal and postpartum period.

# in ref. list	First author, year	Content orientation of intervention	Count of separate intervention*	Exact naming of the intervention	Definition
80	Smoke, 1988	Organization of care	39	Nurse-Midwifery Intervention	Care coordinated by a USA-certified nurse-midwife.
81	Souto, 2020	Maternal mental health	Protocol for SR	Midwife intervention	Intervention to reduce fear of childbirth in pregnant women. Originally, it was called midwifery intervention. In an erratum (2021) this was changed to midwife intervention.
82	Souto, 2021, erratum	Maternal mental health	Erratum	Midwife intervention	In an erratum (2021) this was changed to midwife intervention with the following motivation: "The term ''midwife intervention'' indicates that the intervention is conducted specifically by a health professional with the title of ''midwife,'' which is the population and concept of interest."
83	Souto, 2022	Maternal mental health	SR	Midwives' intervention	All interventions that included a midwife or team of midwives who worked independently or within a multidisciplinary team to reduce fear of childbirth.
84	Spindler, 2018	Professional role	40	A nurse midwife mentoring intervention	No clear definition → debriefing after births to improve skills and knowledge of midwives.
85	Swann, 2012	Birth	41	Midwifery intervention	No explicit definition. Alternatives to the medicalized interventions to promote normal birth.
86	Taylor Miller, 2021	Maternal mental health	SR	Midwifery-led intervention<br>Midwifery-led brief counselling intervention	Intervention targeting post-traumatic stress disorder performed by midwives.
87	Toohill, 2017	Maternal mental health	See 26/30.	A midwife-led psycho-education intervention	No definition. See: Fenwick, J., et al., 2013.
88	Toohill, 2019	Maternal mental health	See 26/30.	A midwife-led psycho-education intervention	No definition. See: Fenwick, J., et al., 2013.
89	Truva, 2021	Public health	42	Midwifery intervention programme	No definition. A personalized prenatal and postnatal structured midwifery intervention programme on increasing breastfeeding rates provided by midwives.

# in ref. list	First author, year	Content orientation of intervention	Count of separate intervention*	Exact naming of the intervention	Definition
90	Türkmen, 2021	Birth	43	Midwifery intervention	No definition. The intervention used thermoforming and massage given by midwives.
91	Türkmen, 2023	Birth	See 75.	Midwifery intervention	No definition. The intervention used rotational ice massage on pregnant women given by midwives
92	Turkstra, 2017	Maternal mental health	See 26/30.	A brief antenatal midwifery psycho-education intervention	The intervention addressing women's expectations and feelings around fear of childbirth.
93	Wallace, 2006	Public health	44	Midwifery intervention	No definition. The protocol required that the intervention a 'hands off' approach to care at first feed was delivered by midwives.
94	Wang, 2021	Maternal mental health	SR	Midwives-led psychological intervention	Interventions provided by midwives on depressive symptoms in perinatal women.
95	Wang, 2023	Public health	45	A midwife-led weight management program	A midwife-led weight management program that facilitates appropriate gestational weight gain for pregnant women, enhances their health literacy during pregnancy, and promotes their experience of antenatal care.
96	Warren, 2017	Public health	46	A brief midwife led intervention	An 'Eat Well Keep Active' intervention programme to facilitate healthful dietary and physical activity behaviours in pregnant women.
97	Wei, 2021	Birth	47	Midwife intervention nursing mode during birth (combined with acupressure).	An mode dominated by midwives is more humanized and targeted to promote vaginal birth versus routine obstetric care.
98	Wilkinson, 2016	Maternal mental health	48	Midwife-led group intervention	An intervention tailored specifically for use with pregnant women experiencing antenatal anxiety, based on the principles of Cognitive Behavioural Therapy (CBT), led by midwives.

* excluding intervention in (systematic) reviews (SR)

Supplement 6: Closer exploration of publications meeting at least 7 out of 8 ICM Philosophy of Midwifery Care components

# in ref. list	First author, year	Title of intervention	1 Pregnancy and childbearing are usually normal physiological processes.	2 Pregnancy and childbearing is a profound experience, which carries significant meaning to the woman, her family, and the community.	3 Midwives are the most appropriate care providers to attend childbearing women.	4 Midwifery care promotes, protects and supports women's human, reproductive and sexual health and rights, and respects ethnic and cultural diversity.	5 Midwifery care is holistic and continuous in nature, grounded in an understanding of the social, emotional, cultural, spiritual, psychological and physical experiences of women.	6 Midwifery care is emancipatory as it protects and enhances the health and social status of women and builds women's self-confidence in their ability to cope with childbirth.	7 Midwifery care takes place in partnership with women, recognising the right to self-determination, and is respectful, personalised, continuous and non-authoritarian.	8 Ethical and competent midwifery care is informed and guided by formal and continuous education, scientific research and application of evidence.
30	Borges, 2021	Caseload midwifery as a legitimate intervention	… to optimise physiological mechanisms of childbirth, while prioritising safe and holistic care …	… promoting cultural experiences of childbearing.	… holistic midwifery care to the diverse UK childbearing population is growing.	Arguably, all women are entitled to holistic care, support for emotional wellbeing and a sense of normalcy.	Caseload midwifery refers to a continuity model … … treated holistically …	… the woman-centred principles of midwifery.	… working in partnership with women	*Evidence is considered when deciding on this intervention for women with CF.*

10	Edqvist, 2017	A multifaceted midwifery intervention	… a multifaceted intervention consisting of 1) spontaneous pushing, 2) all birth positions with flexibility in the sacro-iliac joints, and 3) a two-step head-to-body delivery …	… giving birth is a profound experience which carries significant meaning for the woman and her family.	Settings where the midwifery care includes spontaneous pushing and letting the woman choose her position for birth have been associated with fewer perineal injuries.		The intervention is based on a theoretical framework of woman-centred care …	… care is multifaceted and takes into account the fact that women's expectations, wishes, and labours may differ, thus enabling the midwife to provide woman-centred care.	The intervention involves creating a reciprocal relationship with the woman through presence and participation during labour and birth. … letting the woman choose her position for birth ….	The protective measures supported by evidence …
42	Fenwick, 2013	A midwife-led psycho-education intervention	Intervention that supports normal birth …	Intervention that diminishes the possibility of a negative birth experience.	Intervention offered by midwives …		Connect the event with emotions and behaviours Acknowledge and validate emotions. Enhance social support.	… enhance confidence for labour …	Women's values and experiences must be acknowledged when considering birth options and psychosocial factors are significant to women's	Evidence-based information will be integrated into the counselling session that is specific to the woman's situation.

									decision making.	
46	Gamble, (2005 &) 2017	A midwife-led brief counseling intervention Midwife psycho-education intervention	Supporting women to achieve a normal birth …	Accept and work with women's perceptions of childbirth and maternity services	… pregnancy care was generally provided by midwives.<br><br>… the midwife considered best placed to provide support across multiple issues.		… addressing psychosocial issues in pregnancy was the role of maternity professionals with the midwife considered best placed to provide support across multiple issues.	… increased confidence for birth.	Develop therapeutic relationship with the woman	An evidence-based psycho-education interventio n …
52	Homer, 2013	The intervention : midwifery continuity of care	Midwifery continuity of care will increase the proportion of women who attempt VBAC, increasing the overall rate of vaginal birth and	These questionnaire s will enable women to report on their experiences with the model of care …	Midwifery continuity of care seems to increase the proportion of women who attempt VBAC, increasing the overall rate of vaginal birth and		Women have a midwife caring for them during labour and birth whom they have met before and feel that they know …	… this trusting relationship increases their confidence.	Midwifery continuity of care allows women to develop a relationship with the same caregivers …	*Evidence is considered when deciding on this interventio n for women with CS.*

			reducing the CS rate.		reducing the CS rate.					
60	Lundgren, 2020	MiMO (midwifery model of woman-centred care) intervention	… how to support normal birth.	… women's satisfaction with their labour and birth experiences …	… midwives supporting women's choices and their various ideas about childbirth … … offering a salutogenic and midwifery approach to care …	… increasing community-based services and culturally appropriate care.	A review of midwife-led continuity models of care compared to other models of care revealed fewer interventions; higher satisfaction …	… feelings of enhanced self-concept.	… involving the women in the care (woman-centredness) … … focusing on the aspects of the midwife-woman relationships …	… evidence-based elements are included in the Midwifery 2030 Pathway, which is designed to facilitate the best midwifery practice …

© 2026 Nieuwenhuijze M.J. and Gottfredsdottir H.
